# Deleterious and Adaptive Mutations in Plant Germplasm Conserved Ex Situ

**DOI:** 10.1093/molbev/msad238

**Published:** 2023-11-01

**Authors:** Yong-Bi Fu, Gregory W Peterson, Carolee Horbach

**Affiliations:** Plant Gene Resources of Canada, Saskatoon Research and Development Centre, Agriculture and Agri-Food Canada, Saskatoon, SK S7N 0X2, Canada; Plant Gene Resources of Canada, Saskatoon Research and Development Centre, Agriculture and Agri-Food Canada, Saskatoon, SK S7N 0X2, Canada; Plant Gene Resources of Canada, Saskatoon Research and Development Centre, Agriculture and Agri-Food Canada, Saskatoon, SK S7N 0X2, Canada

**Keywords:** mutation, plant germplasm conservation, RNA-Seq, genetic erosion, mutation burden

## Abstract

Conserving more than 7 million plant germplasm accessions in 1,750 genebanks worldwide raises the hope of securing the food supply for humanity for future generations. However, there is a genetic cost for such long-term germplasm conservation, which has been largely unaccounted for before. We investigated the extent and variation of deleterious and adaptive mutations in 490 individual plants representing barley, wheat, oat, soybean, maize, rapa, and sunflower collections in a seed genebank using RNA-Seq technology. These collections were found to have a range of deleterious mutations detected from 125 (maize) to 83,695 (oat) with a mean of 13,537 and of the averaged sample-wise mutation burden per deleterious locus from 0.069 to 0.357 with a mean of 0.200. Soybean and sunflower collections showed that accessions acquired earlier had increased mutation burdens. The germplasm with more years of storage in several collections carried more deleterious and fewer adaptive mutations. The samples with more cycles of germplasm regeneration revealed fewer deleterious and more adaptive mutations. These findings are significant for understanding mutational dynamics and genetic cost in conserved germplasm and have implications for long-term germplasm management and conservation.

## Introduction

The realized dangers of genetic erosion in plant genetic resources have prompted political and scientific movements around the world to conserve plant genetic resources over the last 60 yr (e.g. Harlan 1972; Pistorius 1997). More than 7 million plant germplasm accessions representing >16,500 plant species are currently conserved in 1,750 genebanks worldwide (FAO 2010). This achievement raises the hope of conserving irreplaceable germplasm and securing the food supply for humanity for future generations (Fowler 2008). However, long-term conservation of such a large volume of diverse germplasm remains a challenging mission, as genetic erosion can also occur within genebanks (Fu 2017). Genebanking represents the most cost-effective ex situ conservation strategy (Li and Pritchard 2009) and was developed for the storage of predominantly orthodox seeds under low seed moisture content and temperature. However, even following FAO standards (FAO 2014) with seed viability tests and regeneration, seeds in long-term storage will lose their viability (Walters et al. 2005) and genetic changes will occur (Roberts 1973). Thus, the risk of genetic erosion exists through genetic drift and nonrandom viability selection within genebanks (Schoen and Brown 2001; Chebotar et al. 2003; Richards et al. 2010; Krishnan et al. 2013). Mutation accumulation in regenerated seed collections was theoretically predicted to lower the viability of conserved germplasm (Schoen et al. 1998), but little is known about the extent of deleterious mutations in conserved germplasm (Dourado and Roberts 1984; Schoen and Brown 2001).

Harmful mutations are long known to harbor in the genomes of individuals (Morgan 1903; Drake et al. 1998) and accumulation of deleterious mutations in a population can lower the population fitness, increasing vulnerability (Muller 1950; Crow 1970; Charlesworth et al. 1993; Lynch et al. 1995). However, the base-substitution mutation rate in all organisms is generally low (<10^−7^ mutations per nucleotide site per generation) (Lynch et al. 2016). The point mutation rate in eukaryotes is further lowered to 1 × 10^−8^ per base pair per generation, although varied widely (Baer et al. 2007). Because of this mutation feature and others (Katju and Bergthorsson 2019), the population inferences of mutation are extremely difficult in any organism (Kondrashov and Kondrashov 2010) and extremely limited in plant species with large and complex genomes, especially using the traditional mutation accumulation approach (Mukai 1964; Charlesworth et al. 1990; Keightley and Eyre-Walker 1999; Schoen 2005; Roles and Conner 2008). Published theoretical and empirical investigations suggest that a large proportion of new mutations, particularly for those in coding portions of the genome, are likely deleterious (Ohta 1972; Gillespie 1994; Eyre-Walker and Keightley 2007) and only a small minority are beneficial (Joseph and Hall 2004), although the distributions of fitness effects for new deleterious and beneficial mutations are similar (Böndel et al. 2022). However, the extent and nature of mutations in many plant species, including crop species, are largely unknown (Schoen 2005; Ossowski et al. 2010; Mezmouk and Ross-Ibarra 2014).

Recent years have seen increased research efforts to identify and characterize deleterious variants across several plant genomes (Lu et al. 2006; Günther and Schmid 2010; Mezmouk and Ross-Ibarra 2014; Renaut and Rieseberg 2015; Kono et al. 2016; Liu et al. 2017; Ramu et al. 2017; Valluru et al. 2019), thanks to the advances in genome sequencing (e.g. wheat; IWGSC et al. 2018), genetic load estimation in the human genome (e.g. Cooper et al. 2005; Henn et al. 2015), and bioinformatics tools for predicting deleterious amino acid polymorphism (e.g. Ng and Henikoff 2003). The identification of deleterious variants across a sequenced genome was largely based on the deleterious prediction of a nonsynonymous site change alone and/or in combination with the intensity of purifying selection inferred from phylogenetic restraints on the site. These efforts have successfully produced useful scans of deleterious variants across plant genomes and informative estimation of mutation burdens in domesticated populations (Moyers et al. 2017; Ramu et al. 2017).

We conducted a large-scale mutation investigation unique to plant germplasm conserved in a seed genebank with the hope of understanding the extent and characteristics of mutation burden in conserved germplasm for better long-term germplasm conservation and utilization. Specifically, 490 individual plants representing the germplasm collections of barley, bread wheat, oat, soybean, maize, rapa (*Brassica rapa* L.), and sunflower were sequenced using RNA-Seq technology (Wang et al. 2009) (supplementary table S1, Supplementary Material online). Deleterious variants were detected across each genome based on the scores of both Sorting Intolerant From Tolerant (SIFT; Vaser et al. 2015) and Genomic Evolutionary Rate Profiling (GERP; Davydov et al. 2010) and characterized in frequency, expression, and effect for each collection and paired groups of samples with contrasting conservation features (supplementary table S2, Supplementary Material online). Such characterizations also allow for assessments of mutational changes in a germplasm collection with respect to variable conservation practices. Specifically, we asked if conserved germplasm, which was acquired earlier, stored for a longer period, had more regenerations or lower germination levels, would have more deleterious and fewer adaptive mutations.

## Results

### The Extent and Characteristics of Mutations in 7 Germplasm Collections

**Fig. 1.**
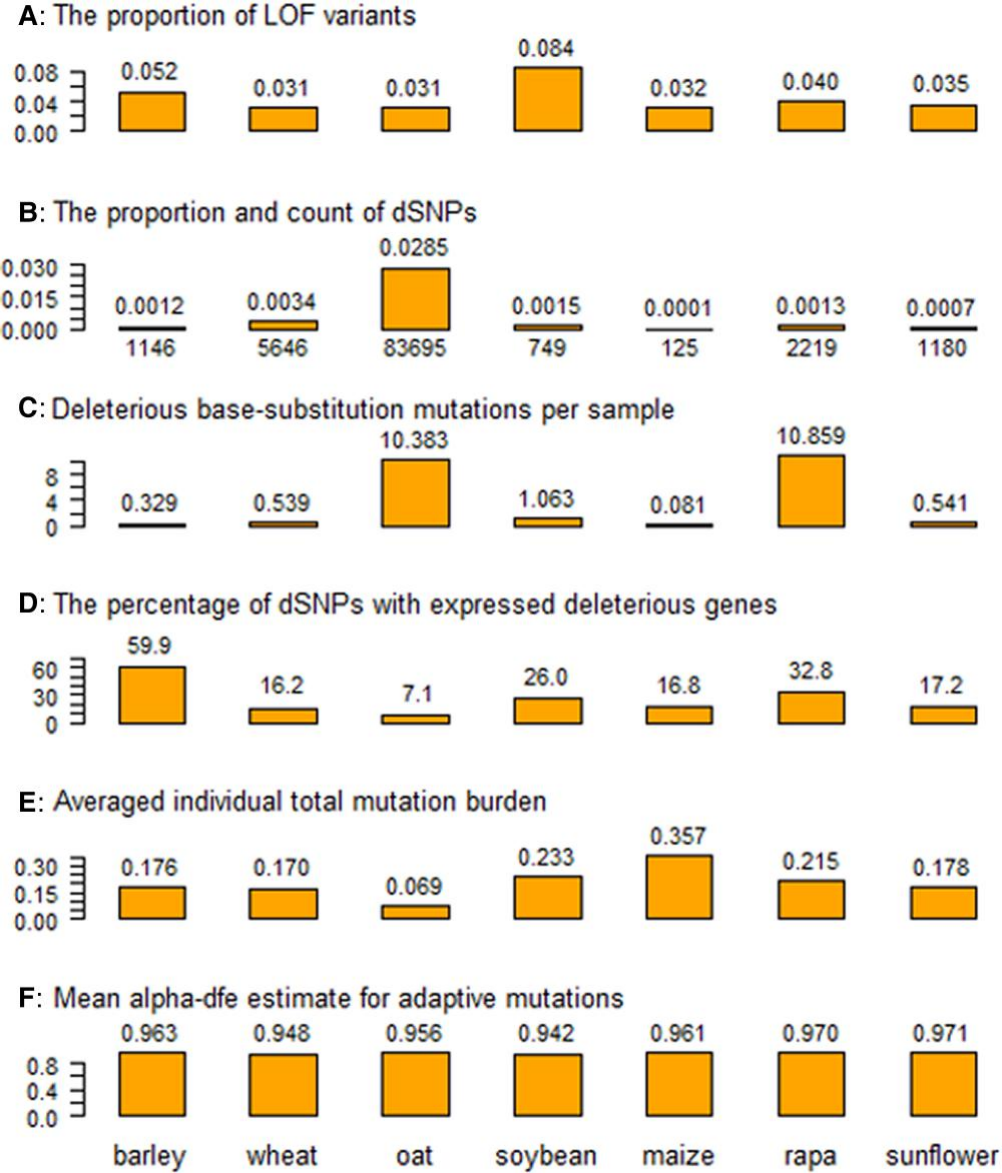
Six estimates of deleterious and adaptive mutations from the samples of the seven germplasm collections (barley, wheat, oat, soybean, maize, rapa, and sunflower). Panel A shows the proportion of loss-of-function (LOF) variants over all detected variants in a collection; panel B displays the count and proportion of deleterious SNPs (dSNPs) over all detected variants; panel C displays the estimate of deleterious base-substitution mutations per sample (×10^−8^); panel D illustrates the percentage of the dSNPs associated with expressed deleterious genes; panel E plots the estimate of averaged sample-wise total mutation burden per deleterious locus; and panel F displays the estimate of mean alpha-dfe for adaptive mutations.

We performed sequence analysis following the major steps outlined in supplementary fig. S1 to identify genomic variants and perform single nucleotide polymorphism (SNP) annotations. The analysis generated a range of 4.6 to 18.0 million mapped sequence reads (MSR) per collection sample with a mean of 11.1 million MSR (supplementary table S3, Supplementary Material online). Calling genomic variants including SNPs using ANGSD (Korneliussen et al. 2014) identified a range of 0.51 to 2.94 million SNPs for the seven collections with a mean of 1.56 million SNPs (supplementary table S4, Supplementary Material online). SNP annotation using Ensembl-Variant Effect Predictor (VEP; Naithani et al. 2017) allowed for the classification of SNPs into 17 different classes (supplementary table S4, Supplementary Material online). We also assessed the proportions of the detected variants associated with loss of function (LOF) and the proportions of LOF variants were found to vary for these collections from 0.031 (wheat and oat) to 0.084 (soybean) with a mean of 0.027 (Fig. 1A; supplementary table S4, Supplementary Material online).

We identified deleterious SNPs (dSNPs) based on both SIFT and GERP++ “rejected substitution” (RS) scores. The SIFT score presents a prediction on the impact of an amino acid substitution and can distinguish between functionally neutral and deleterious amino acid changes. An amino acid substitution with a SIFT score of 0.05 or less is considered to be deleterious. GERP++ produces an RS score to quantify the conservation of each nucleotide in a multispecies alignment (supplementary table S5a, Supplementary Material online). A positive score (RS > 0) at a substitution site means fewer substitutions than expected. Thus, a substitution occurring in a site with RS > 0 is predicted to be deleterious; the larger the RS score, the more deleterious the substitution. The overall frequency distributions of the derived RS scores across a genome are shown in supplementary table S5b for the seven species. In total, a range of dSNPs was identified from 125 (maize) to 83,695 (oat) with an average of 13,537 for the seven collections (supplementary table S4, Supplementary Material online). The dSNPs detected from each collection were located on every chromosome across a genome (supplementary table S5c, Supplementary Material online). Weighing by all the detected SNPs, the proportions of dSNPs varied from 0.00006 (maize) to 0.02849 (oat) (Fig. 1B; supplementary table S4, Supplementary Material online). Similarly, based on the frequency estimates from the 70 assayed samples, we also identified a range of fixed dSNPs from 5 (maize) to 215 (oat) with a mean of 58. To understand the variation of these dSNPs, we assessed the deleterious allele frequency distributions and found that the majority of dSNPs in each collection had low allelic frequencies and that some dSNPs were fixed in each collection (supplementary fig. S2, Supplementary Material online). For example, more than half of the detected dSNPs had allelic frequencies of 0.2 or smaller, and 37 dSNPs were fixed in the 70 barley samples.

Efforts were also made to estimate deleterious base-substitution mutations per sample (dBSMs) for the seven collections based on their estimates of dSNPs and fixed dSNPs and published genome sequence length of a species (supplementary table S6, Supplementary Material online). Considering dSNPs, the estimates of dBSMs (×10^−8^) for the seven collections varied widely from 0.081 (maize) to 10.859 (rapa) with a mean of 3.399 (Fig. 1C). For fixed dSNPs, the estimates of dBSMs (×10^−8^) for the seven collections ranged from 0.0033 (maize) to 0.2056 (rapa) with a mean of 0.0380 (supplementary table S6, Supplementary Material online). We also examined the changes in dBSMs based on the storage years within a collection (supplementary table S7, Supplementary Material online). There were four collections displaying positive changes in dBSMs for dSNPs over the storage years and five collections showing negative changes in dBSMs for fixed dSNPs.

RNA-Seq data also allowed for the inference of expressions for those deleterious genes identified by dSNPs (supplementary table S4, Supplementary Material online). The percentages of dSNPs associated with expressed deleterious genes over all the detected dSNPs ranged widely among the seven collections from 7.1% (oat) to 59.9% (barley) with a mean of 25.2% (Fig. 1D). Thus, roughly one-quarter of the identified deleterious genes were expressed in the 3-leaf stage of monocots and the true leaf stage of dicots. The mean expression levels measured in transcripts per million (TPM) for those expressed deleterious genes varied considerably for the seven collections. Weighing by gene counts and sample sizes, for example, the estimate of mean TPM/gene/sample ranged from 3.80 (oat) to 25.31 (rapa) with a mean of 10.51 (supplementary table S4, Supplementary Material online).

We also characterized biological activities expressed by deleterious genes in the paired storage year (SY) group of samples via Blast2GO (Conesa and Götz 2008) and REVIGO (Supek et al. 2011) (supplementary table S8, Supplementary Material online). The expressions of all the detected dSNPs were largely involved with protein phosphorylation, organic substance metabolism, and responses to chemical, stress, and stimulus. Some distinct biological processes such as carbohydrate derivative biosynthesis were also identified for each paired group (supplementary table S8a, Supplementary Material online). The expressions of fixed dSNPs were mainly associated with the cellular process, macromolecule metabolism, nitrogen compound metabolism, and metabolism. Some distinct biological processes such as the cellular metabolism for fixed dSNPs were also identified for each paired group (supplementary table S8b, Supplementary Material online).

To assess mutation burden, we counted deleterious heterozygotes and homozygotes for each dSNP in each individual sample to estimate sample-wise mutation burdens per deleterious locus in each collection with respect to deleterious heterozygote, deleterious homozygote, and total burdens (supplementary table S4; supplementary fig. S3, Supplementary Material online). These sample-wise mutation burdens varied within a collection (supplementary fig. S3, Supplementary Material online) and among collections (Fig. 1E). Specifically, the averaged estimates of individual total burden ranged from 0.069 (oat) to 0.357 (maize) with an overall mean of 0.200 (Fig. 1E). The estimated individual homozygous burdens varied from 0.012 (oat) to 0.194 (maize) with a mean of 0.106 (supplementary table S4, Supplementary Material online). The estimated individual heterozygous burdens ranged from 0.058 (oat) to 0.163 (maize) with a mean of 0.093 (supplementary table S4, Supplementary Material online).

We also estimated the proportion of adaptive substitutions in each collection using PolyDFE (Tataru et al. 2017) to infer the extent of adaptive mutations (Eyre-Walker and Keightley 2007). PolyDFE generates alpha-dfe statistic as the proportion of adaptive substitutions (with a selection coefficient greater than 0) from site frequency spectrum data. Such a statistic does not provide a direct count of adaptive mutations, but a higher alpha-dfe estimate suggests relatively more advantageous mutations in the group of samples. It was found that all 7 collections revealed similar extents of alpha-dfe estimates ranging from 0.942 (soybean) to 0.971 (sunflower) with an overall mean of 0.959 (Fig. 1F; supplementary table S4, Supplementary Material online).

### Evidence for Mutational Changes in Conserved Germplasm

**Fig. 2.**
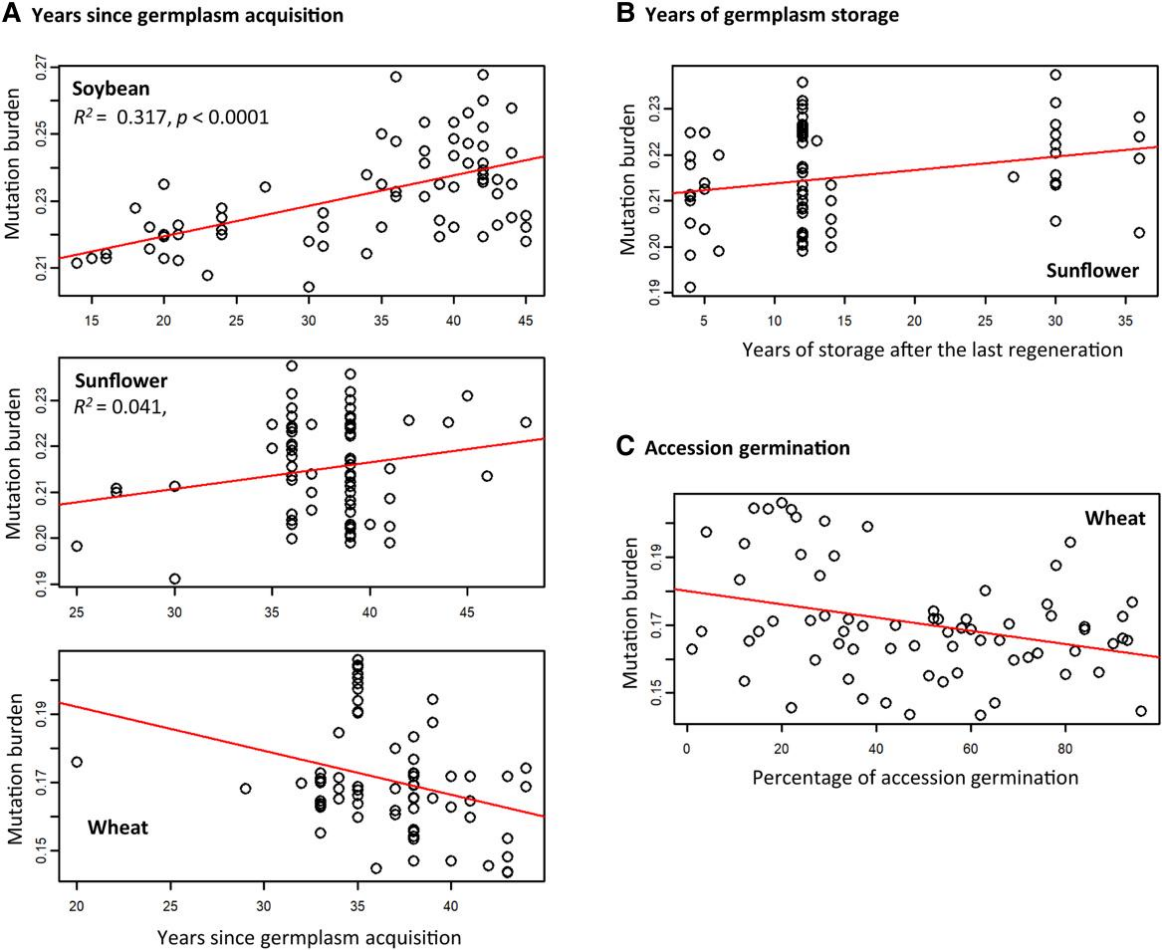
Some collections display significant associations of individual total mutation burden with the years since germplasm acquisition (A), the years of germplasm storage after the last germplasm regeneration (B), and/or the percentages of accession seed germination (C).

We assessed the associations between individual mutation burdens and accession characteristics such as the years since the accession acquisition, the storage years since the last accession regeneration, and the germination levels (supplementary fig. S4, Supplementary Material online). Several significant associations were found (Fig. 2). First, soybean and sunflower collections displayed that accessions acquired earlier had higher total mutation burdens, while the wheat collection showed a decreased total mutation burden in the accessions acquired earlier (Fig. 2A). Second, the sunflower collection revealed an increased total mutation burden in the accessions stored longer after the last regeneration (Fig. 2B). Third, the wheat collection displayed the lower mutation burden in the accessions with higher germination levels (Fig. 2C).

**Fig. 3.**
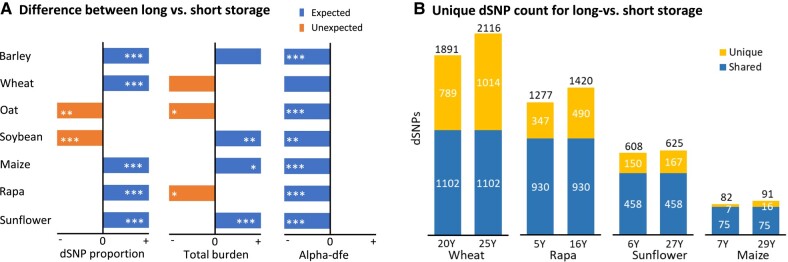
Some collections display increased proportions of dSNPs over all detected variants, high individual total mutation burden and lowered alpha-dfe values for adaptive mutations when accessions were stored over a long time (A). The expected and unexpected differences in panel A for dSNP proportion and total burden are shown in right blue and left orange boxes (but un-scaled for ease of illustration), respectively, while all the differences for alpha-dfe are expected and shown in left blue boxes. The stars within each box stand for the statistical significance (*P < 0.05; **P < 0.01; ***P < 0.001). These expectations were further supported with the increased count of unique dSNPs in the accessions with more storage years (B) in four collections. For example, the wheat collection shows the samples with 25 yr of storage or longer had 1,014 unique dSNPs, while the samples with 20 yr of storage or shorter had 789 unique dSNPs. Unique and shared dSNP counts in panel B are shown in top yellow and bottom blue bars, respectively.

We also made a comparative analysis of the extent of dSNPs, total mutation burden, and alpha-dfe estimates between samples of each collection with more or fewer years of storage since the last regeneration (supplementary table S9, Supplementary Material online). Some collections were found to display increased proportions of dSNPs over all the detected variants, high individual total mutation burden, and lowered alpha-dfe values for adaptive mutations when accessions were stored over a long time (Fig. 3A). For example, the samples with more years of storage showed an increase in the proportion of dSNPs in five (out of seven) collections and in total mutation burden in four collections and a decrease in alpha-dfe estimates for adaptive mutations in all seven collections. These findings were further supported by the increased counts of unique dSNPs in the accessions with more years of storage in some collections (Fig. 3B; supplementary fig. S5, Supplementary Material online). For example, the wheat samples with 25 or more yr of storage since the last regeneration had 1,014 unique dSNPs, while those samples with 20 yr or shorter of storage had 789 unique dSNPs. However, there were no marked differences in deleterious allelic frequency distribution with respect to the year of storage between SY1 and SY2 groups in each collection (supplementary fig. S6, Supplementary Material online).

**Fig. 4.**
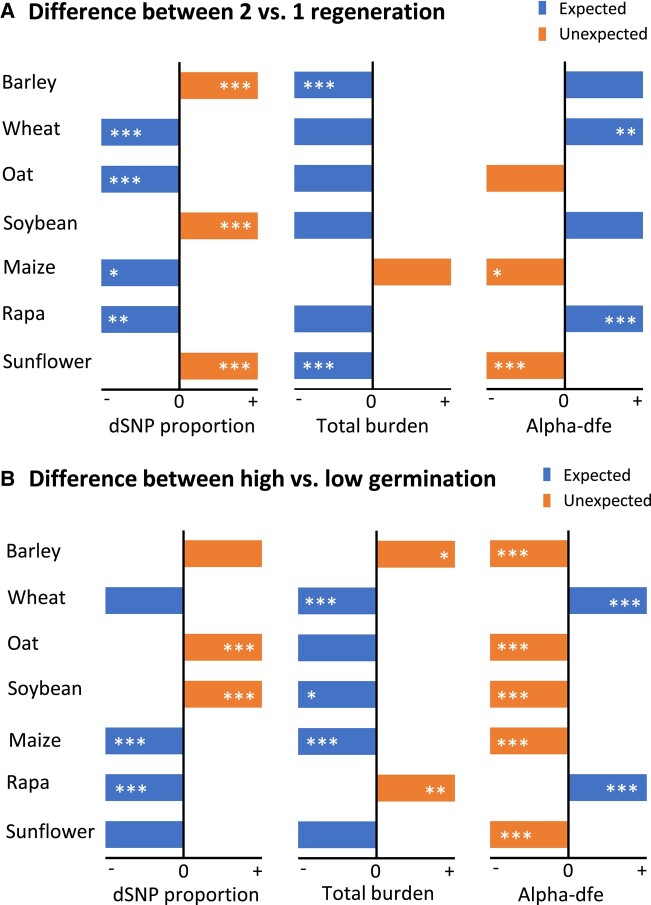
Mutational differences between two paired groups of samples [with 2 versus 1 germplasm regeneration (A); with high versus low germination levels (B)] in the proportion of dSNPs over all detected variants, individual total mutation burden and alpha-dfe values for adaptive mutations. The expected and unexpected differences for dSNP proportion and total burden are shown in left blue and right orange boxes (but un-scaled), respectively, while the expected and unexpected differences for alpha-dfe are shown in right blue and left orange boxes. The stars within each box stand for the statistical significance (*P < 0.05; **P < 0.01; ***P < 0.001).

We reasoned that germplasm regeneration may remove some deleterious mutations, reduce individual mutation burden, and increase adaptive mutations. By comparing samples of a collection with 2 versus 1 germplasm regeneration, we found that the samples with 2 cycles of germplasm regeneration displayed a reduction in the proportion of dSNPs in four collections and in total mutation burden in six collections and an increase in alpha-dfe estimates for adaptive mutations in four collections (Fig. 4A; supplementary table S10, Supplementary Material online). There were two collections (wheat and rapa) with three estimates consistent with the reasoning. Similar comparisons were also made between samples of a collection with high versus low germination levels (supplementary table S11, Supplementary Material online). It was found that the samples with high germination levels displayed a reduction in the proportion of dSNPs in four collections and in total mutation burden in five collections and an increase in alpha-dfe estimates for adaptive mutations only in two collections (Fig. 4B). Only the wheat collection showed the three estimates matched with the reasoning.

## Discussion

Our study represented a large-scale mutation investigation unique to plant germplasm conserved ex situ worldwide, generated a novel set of mutation findings that have rarely been achieved before in an ex situ germplasm collection, and identified many unique features of deleterious mutations present in conserved germplasm. We also found some evidence of mutational changes in the seven germplasm collections. For example, soybean and sunflower collections displayed that early acquired accessions had increased total mutation burdens. The germplasm with more years of storage in many collections showed higher mutation burdens and lower alpha-dfe values for adaptive mutations. Thus, there is a genetic cost of conservation present in the germplasm collections, and consequently, mitigating measures to minimize mutation burdens need to be developed for better long-term germplasm conservation and utilization.

We reasoned that deleterious mutations will accumulate in germplasm conserved ex situ over time and such mutational changes can be associated with some conservation features. Our study revealed some genomic evidence for mutational changes over time in some collections (Figs. 2 and 3). However, the patterns of mutational changes were not always consistent across the seven collections. For example, the samples with more storage years displayed an increase in the proportion of dSNPs in five collections and in total mutation burden in four collections, and a decrease in alpha-dfe estimates for adaptive mutations in all seven collections (Fig. 3A). Similarly, the samples with high germination levels displayed expected variations in total mutation burden only in five collections and in alpha-dfe estimates for adaptive mutations only in two collections (Fig. 4B). The inconsistency may have resulted from both technical and biological factors. Technical ones can include bias in sampling, sequence quality, and genomic complexity, as discussed below. It may also reflect the effects of differential viability, regeneration, and genetic drift on the assayed germplasm since acquisition (Charlesworth et al. 1993; Schoen et al. 1998), with some supportive evidence from the analysis of mutational differences with more regenerations (Fig. 4A). For example, the samples with two cycles of germplasm regeneration displayed a reduction in the proportion of dSNPs in four collections (supplementary table S10, Supplementary Material online), which clearly indicates the impact of germplasm regeneration. Further investigations on the same sets of conserved germplasm with specific conservation features would be more informative to assess the reasoning. More importantly, research is needed to elucidate the mechanisms of genetic mutations induced under long-term cold storage (e.g. see Dourado and Roberts 1984; Puig et al. 2015).

The estimates of mutation abundance in the seven collections are generally lower than those reported in a few plant species (e.g. Mezmouk and Ross-Ibarra 2014; Renaut and Rieseberg 2015; Kono et al. 2016, 2019; Liu et al. 2017; Ramu et al. 2017; Lozano et al. 2021). For example, there were 12,759 dSNPs (on average) detected in 19 domesticated sunflower accessions based on RNA-Seq data (Renaut and Rieseberg 2015), 3,855 dSNPs in 21 barley lines based on exome capture data (Kono et al. 2019), and 3,041 dSNPs in 8 soybean accessions based on the whole genome re-sequencing data (Kono et al. 2016). Such discrepancy may largely reflect the variations in the use of dSNP identification method, sample size, sequence type, and the extent of GERP++ RS sites. For example, our estimates mainly reflected the expressed or transcribed deleterious mutations specific to a developmental stage and were expected to be lower than those based on the whole genome re-sequencing (e.g. Kono et al. 2016) or exome capture (e.g. Kono et al. 2019) data. Our inferences were more conservative with the RS sites generated from 12 reference genomes, relative to those with 7 or fewer reference genomes (e.g. Ramu et al. 2017 ). However, the estimates of dBSMs in the seven collections (supplementary table S6, Supplementary Material online) are compatible with those mutation abundances inferred from the published mutation rates in other organisms (Baer et al. 2007; Ossowski et al. 2010; Katju and Bergthorsson 2019).

The variations in individual total mutation burden per deleterious locus among the seven collections were statistically significant, indicating that these collections harbored variable levels of deleterious mutation burdens. Interestingly, the collections of selfing plants (barley, wheat, oat, and soybean) generally seemed to have a lower total mutation burden (0.162 on average) than those of outcrossing crops (maize, rapa, and sunflower; 0.250) (see Fig. 1E). Similarly, there was more homozygous mutation burden and less heterozygous mutation burden in the collections of selfing than outcrossing crops (supplementary table S4, Supplementary Material online). These findings are expected as the samples of selfing crops generally have fewer heterozygous genotypes and homozygous mutations can be easily purged (Charlesworth et al. 1993). A linear regression analysis of the resulting data (in supplementary table S6, Supplementary Material online) revealed a nonsignificant increase in dSNP counts over the seven genome sizes, but two hexaploid collections (wheat and oat) displayed substantially more dSNPs than those diploid collections. Thus, the patterns of mutation burden seem to be somehow associated with some life history traits and biological features of a species (Chen et al. 2022).

The results generated from the seven germplasm collections are encouraging for mutation investigation of conserved germplasm of other plant species with sequenced genomes, as they demonstrated the effectiveness of identifying and characterizing abundant genome-wide deleterious variants. Following our RNA-Seq based approach (supplementary fig. S1, Supplementary Material online) to investigate genome-wide deleterious variants in other plant species will be fruitful. As more plant genomes have been sequenced (see Ensembl Plants at http://plants.ensembl.org/index.html), such mutation investigations will be more feasible than before. With decreasing cost of sequencing, it is possible to increase sample size using our RNA-Seq method or apply the whole genome re-sequencing for more powerful identification and characterization of deleterious mutations in many plant species. Conserved germplasm in genebanks is an excellent study system for conservation genomics to characterize temporal genomic erosion in many conserved plant species (Allendorf et al. 2010; Díez-Del-Molino et al. 2018).

Our RNA-Seq application is currently more cost-effective and feasible than the other genomic methods such as exome capture and whole genome sequencing. We focused on the detection of deleterious genes expressed in the early seedling stage representing the viable germplasm after storage. Such detection helped to minimize potential confounding of environmental effects. However, the detection considered only the expressed or transcribed deleterious mutations and the findings may be completely specific to a developmental stage. It would be useful to further identify and characterize deleterious mutations in other tissues and developmental stages. Also, our germplasm sampling was not ideal, considering that it was largely based on the availability of conserved germplasm with the records of acquisition and storage years and regeneration, and did not emphasize the purity of genetic materials and separate storage conditions (4 °C in active collections and −18 °C in base collections) for the assayed samples. Both the genetic diversity of the assayed samples and mixed storage conditions could compound the mutational inferences. For example, the ideal samples for the paired storage year groups should consist of the same germplasm that can be divided into multiple sets, each conserved over variable years under the same storage condition. Moreover, the mutation inference was dependent on the quality of sequencing data, assembled reference genomes, and sample size. The application of 2 replications in the sequencing of each collection (supplementary table S2, Supplementary Material online) allowed for better removal of some mis-alignments and other artifacts and for biological comparison of some research outputs, but we cannot fully remove all the technical errors from various bioinformatical analyses. It is not surprising to detect the smallest dSNPs in the maize samples due to the complexity of the maize genome (Mezmouk and Ross-Ibarra 2014). Thus, the bias in mutation estimation could not be fully excluded. Despite these caveats, however, this unique study should provide some starting points for further empirical investigations on mutation dynamics of conserved germplasm and crop plants (Wang et al. 2017; Gaut et al. 2018).

Our research findings have several practical implications for germplasm management and conservation. First, the estimated mutation burdens confirmed that there is a genetic cost for plant germplasm conserved in genebanks (Schoen et al. 1998), and the risk of declining fitness exists for germplasm imposed by a long-term accumulation of deleterious mutation (Charlesworth et al. 1993). Thus, it is important to consider mutation as a cost factor in genebank management practices with the goal to minimize the extent of mutation accumulation in conserved germplasm and the risk of germplasm loss (Schoen and Brown 2001). The genetic integrity of conserved germplasm can change over time. Unfortunately, effective mitigating measures have not been developed yet and are currently missing in worldwide genebank operations (FAO 2014). Second, different germplasm collections carried different levels of mutation burden, and germplasm of outcrossing species seemed to carry a higher mutation burden than those of selfing crops, implying different mitigating measures may be needed for conserving germplasm of different mating types. Third, the finding of the mutational differences between the samples of contrasting storage years implies the need for more vigorous seed viability tests to determine the optimal time (or years) of germplasm storage with respect to mutation burden. Fourth, associating mutation burden with known accession features such as regeneration frequency revealed little power to predict the extent of mutation burden in conserved germplasm, implying that mutation accumulation over regenerations is complicated. Fifth, our estimated mutation burdens were predictive in nature and research is needed on the fitness consequence of these deleterious mutations on the conserved germplasm, although phenotypic mutations induced during storage in barley and pea seeds were evident earlier (Dourado and Roberts 1984). Together, our study showed that mutation accumulation in genebanks is more complex than anticipated with regeneration alone (Schoen et al. 1998). The need is not over-emphasized for further research on the dynamics of mutation accumulation in genebanks to develop effective conservation strategies for minimizing the within-genebank genetic erosion from deleterious mutations.

## Materials and Methods

Materials used for this study and methods used for collecting samples, RNA extractions, sequencing, SNP calling and annotation, measuring mutation burden, characterizing deleterious mutations, gene ontology analysis, estimation of base-substitution mutation, and data and code availability are described in detail and available in the Supporting Information Appendix. The Supporting Information Appendix has five components: (i) Supplemental materials and methods; (ii) summary results on variant identification and annotation; (iii) references for materials and methods; (iv) Tables S1 to S11; and (v) Figs. S1 to S6.

## Supplementary Material

msad238_Supplementary_Data

## Data Availability

Acquired original RNA-Seq data (supplementary table S1, Supplementary Material online) were deposited in NCBI's SRA database. The supplemental output or meta data for each species (dSNP annotation, SIFT and GERP++RS scores) described in Section A11 of Supporting Information Appendix were deposited into Figshare (https://doi.org/10.6084/m9.figshare.12234431). Custom Perl, Shell, or related pipelines that we generated for the bioinformatics analyses of these RNA-Seq data and R scripts for the generation of all figures are available upon request to the corresponding author.
