# Correction to: Deleterious and Adaptive Mutations in Plant Germplasm Conserved Ex Situ

**DOI:** 10.1093/molbev/msaf098

**Published:** 2025-05-21

This is a correction to: Yong-Bi Fu, Gregory W Peterson, Carolee Horbach, Deleterious and Adaptive Mutations in Plant Germplasm Conserved Ex Situ, *Molecular Biology and Evolution*, Volume 40, Issue 12, December 2023, msad238, https://doi.org/10.1093/molbev/msad238

In the published version of this manuscript, Figure 2 contains incomplete information on linear regression results due to improper formatting of the figure for paper production. The sub-figure for soybean in panel A, for example, has the complete information on the regression results (R-square and P value), while the other subfigures have little or no information. The corrected Figure 2, shown below, contains all the intended information on the regression results.

Corrected Figure 2:

**Figure:**
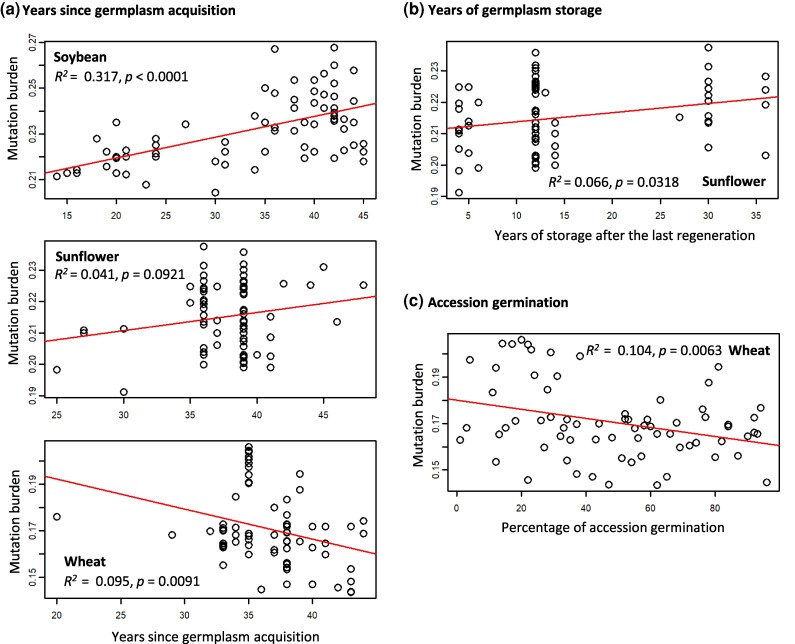


These details have been corrected only in this correction notice to preserve the published version of record.

