# Supplementary material for: Deleterious and Adaptive Mutations in Plant Germplasm Conserved Ex Situ
Source: Mol Biol Evol. 2023 Nov 1;40(12):msad238. doi: 10.1093/molbev/msad238 (PMC10724023; doi:10.1093/molbev/msad238)

Molecular Biology and Evolution https://doi.org/10.1093/molbev/msad238

Deleterious and adaptive mutations in plant germplasm conserved *ex situ*

Yong-Bi Fu, Gregory W. Peterson and Carolee Horbach

Supporting Information Appendix

A. Supplemental materials and methods
B. Summary results on variant identification and annotation
C. References for materials and methods
D. Tables S1 to S11
E. Figures S1 to S6

A. Supplemental materials and methods

A1. Study materials

We studied 490 plant samples representing 70 different accessions of variable conservation years and regenerations from each of seven plant germplasm collections held at Plant Gene Resources of Canada (PGRC), Saskatoon, SK, Canada (Tables S1 and S2). PGRC was established in 1970 as the Canadian national genebank and currently conserves 121,000 germplasm accessions representing more than 900 plant species (https://pgrc.agr.gc.ca/index_e.html). Each collection generally consists of subspecies, landraces, cultivars, and breeding lines. Seed germplasm is conserved under two controlled storage environments to maintain seed viability: 4°C with a relative humidity of 25-35% for active collections and -18°C for base collections, each with a seed moisture content of 8-10%. Germplasm management follows FAO genebank standards (FAO 2014) with seed viability tests, regeneration, evaluation, and characterization.

The seven plant germplasm collections studied were: barley (*Hordeum vulgare* L.), bread wheat (*Triticum aestivum* L.), oat (*Avena sativa* L.), soybean (*Glycine max* (L.) Merr.), maize (*Zea may*s L.), rapa (*Brassica rapa* L.) and sunflower (*Helianthus annuus* L). The relevant biological information for these seven crops was given in Table S1a. We selected these collections by considering (1) the availability of a reference genome for the species, (2) the collection with records of acquisition and storage years and regeneration frequency, (3) the collection size, and (4) the mating type of the germplasm (selfing vs. outcrossing). Accession selection of a collection was randomly made from the available germplasm with records of acquisition and storage years and regeneration frequency. The seed samples of each selected accession were acquired from Mr. Dallas Kessler, PGRC, along with their genebank inventory information, including the passport data, the year acquired and last regenerated, and the regeneration frequency (Table S2). Note that the majority of seed samples were selected from the base collection, but some seed samples were selected from the same accession conserved in the active collections due to the seed availability.

To assess the differences in mutation in the conserved samples, we formed three specific paired groups of the assayed samples in each collection: 1) storage year (SY), the samples with the highest and lowest numbers of storage years since the last regeneration; 2) regeneration frequency (RF), the samples with one versus two regenerations since the acquisition; and 3) germination difference (GD), the samples of the accessions with low versus high germination

rates. The grouped samples with contrasting accession features were randomly selected with roughly equal group sizes from the 70 assayed samples in each collection. The composition of these paired groups in each collection is shown in Table S2. For example, the barley SY group "barley 10Y" consisted of 16 samples representing the accessions conserved in the genebank for 10 or fewer years since the last regeneration. Similarly, the SY group of "barley 27Y" consisted of 16 samples representing the accessions conserved for 27 or more years since the last regeneration.

A2. Germination testing

Germination tests were conducted with four replications of 25 seeds for each assayed accession in the seven germplasm collections. Tests were performed on rolled germination paper (Anchor Paper, St. Paul, MN, USA) in a germination cabinet (Hoffman Manufacturing Inc., Albany, OR, USA) following the Association of Official Seed Analysts (AOSA 2013) guidelines for each species. The percentage of normally germinated seedlings was assessed following the AOSA Rules for Testing Seeds (2013) and the ISTA Handbook on Seedling Evaluation (2009). All germination test results were documented in Table S2.

A3. RNA extraction

Seeds for each accession were randomly selected and planted in the greenhouse at the Saskatoon Research and Development Centre, Saskatoon, SK, Canada. Seedlings were grown in a 96-cell seedling tray in a greenhouse set to 16-hour days with 22°C day temperature and 16°C night temperature. Approximately 2 cm (75-100 mg) of monocot tissue was collected at the three-leaf stage from the region just above the root collar containing the apical meristem. The tissue was placed in a 2 ml tube, frozen with liquid nitrogen, and stored at -80°C. Dicot tissue was collected when the first true leaf was open and before stem elongation. The true leaves above the cotyledons and the apex were collected, placed in a 2 ml tube, and frozen with liquid nitrogen. Tissue was stored at -80°C. RNA was extracted using a Qiagen RNeasy Plant Mini Kit (Qiagen Inc., Toronto, ON) with Buffer RLT, following the provided protocol. After extraction, all RNA samples were treated using an Invitrogen DNA-free™ DNA Removal Kit (ThermoFisher Scientific, Waltham, MA, USA). RNA was quantified using a Thermo Scientific Nanodrop 8000 and several samples were separated on a Bioanalyzer RNA 6000 Nano Chip (Agilent, Santa Clara, CA, USA) to check the RNA Integrity Number (RIN). All samples were diluted to 50 ng/µl using RNase-free BPC Grade water (Sigma-Aldrich Canada, Oakville, ON).

A4. Sequencing

RNA-Seq libraries were prepared using a SENSE mRNA-Seq Library Prep Kit V2 (Lexogen Inc., Greenland, NH, USA) following the method supplied with the kit. Briefly, 1000 ng of total RNA was poly-A selected through hybridization to selection beads. Bead-bound RNA was reverse-transcribed with Reverse Transcription and Ligation Mix RTL for long paired-end sequencing runs. Second-strand synthesis and clean-up were carried out as per the protocol. Single indexes and Illumina adapters were added during library amplification, using 10-12 cycles of PCR, followed by sample clean-up, as per the protocol. Three libraries of 24 samples were prepared for each collection. Prior to the library preparation for each of the seven collections, one RNA sample was randomly selected to act as a technical replicate in each of the three multiplexed libraries for a total of 70 unique samples. Amplified libraries were assessed for fragment size and overall yield using the Bioanalyzer 2100 High Sensitivity DNA assay (Agilent

Technologies Inc., Santa Clara, CA, USA). Equimolar amounts of each library were pooled. Prepared libraries were sequenced at the National Research Council of Canada's DNA sequencing laboratory (Saskatoon, SK) using the Illumina HiSeq 2500, Version 4 chemistry, 2x 125-cycle paired-end reads, with one library of 24 samples per HiSeq lane (Table S1).

A5. Sequence alignment and substitution scores

A5a. Generating BAM files

A pair of demultiplexed FASTQ files were generated for each sample: one in the forward and another in the reverse direction. FastQC (Babraham Bioinformatics; https://www.bioinformatics.babraham.ac.uk/projects/fastqc/) was applied to assess the overall sequencing quality for each collection sample. Sample FASTQ files were trimmed with Trimmomatic v0.32 (Bolger et al. 2014) to remove residual Illumina adapter sequence, trim low-quality sequence below an average Phred score of 24 over a 10-base window and any sequence shorter than 80 bases (Table S3). FastQC was again run to verify that the Illumina adapter sequences and low-quality reads were removed. Reference genome sequences for each species were obtained from EnsemblPlants (Table S5a) with one exception. The split-chromosome version of barley (Mascher et al. 2017) was obtained from Dr. Martin Mascher, IPK Gatersleben, Germany. The split-chromosome barley reference has the same number of bases and is similarly masked as the "160404_barley_pseudomolecules_masked.fasta" (Mascher et al. 2017) and the EnsemblPlants v40 version of the reference. However, the split-chromosome reference, rather than the full-length chromosome reference, was used since the number of bases in each chromosome FASTA entry is below the 2^{39}-1 base (≈536 Mb) limit of Samtools (Li et al. 2009, https://www.htslib.org) BAM indexing. For the same reason, each wheat and oat reference chromosome sequence (IWGSC et al. 2018; Kamal et al. 2022) was split in half using a custom Perl script. Each species was aligned against its respective genome sequences using the Burrow-Wheeler Aligner v1.7 (Li and Durbin 2010) BWA-MEM algorithm. The resulting BAM files were filtered to remove PCR duplicates with MarkDuplicates and to apply base quality score recalibration (BQSR) using BaseRecalibrator and ApplyBQSR: components of the Genome Analysis Toolkit (GATK; Van der Auwera et al. 2013) v4.0.1.0 tools. Table S3 summarizes the number of mapped sequence reads for each sample. This effort generated a BAM file for each assayed sample of the seven collections.

A5b. Sorting intolerant from tolerant (SIFT) substitution score

We applied SIFT algorithm (Vaser et al. 2015) to predict the deleterious effect of a single nucleotide polymorphism (SNP) detected with a SIFT score. The stand-alone Ensembl Variant Effect Predictor (VEP) (McLaren et al. 2016; Naithani et al. 2017) could only directly generate a SIFT score for a SNP from barley and wheat ANGSD-generated VCF files because only these VEP species caches were associated with a SIFT database. Thus, separate SIFT analyses were needed for the other five species. To do so, we first developed a SIFT prediction database for each species following the instructions of SIFT4G and then executed the SIFT4G annotator based on ANGSD-generated VCF file as shown below. Considerable effort was made to set up the SIFT prediction databases and to format some FASTA, GTF, or GFF files using custom Perl scripts or command lines, as published genome sequences in some species such as sunflower were not consistent in the format required by SIFT. Additional effort was also made with custom scripts to filter the SIFT predictions of SNPs with deleterious, deleterious with low confidence,

tolerated, and tolerated with low confidence. Note that the five SIFT databases (for oat, soybean, maize, rapa, and sunflower) are accessible as described in **A11** below.

A5c. GERP++ Rejected substitution score

We also applied GERP++ (Davydov et al. 2010) to measure the phylogenetic constraint from the substitution of a locus by generating a Rejected Substitution (RS) score. Gerpcol, specifically, estimates constraint for each column of an alignment of several genomes of increasing taxonomic distance. Thus, considerable efforts were made to acquire multiple genome sequence alignments for the seven species. Table S5a lists the related crop species used to do multiple genome sequence alignments for each of the seven studied species, along with their reference genome sequence information and citations. Note that the same reference genome sequence files were used as those for SNP identification with minor exceptions of formatting for barley. All genome sequences to be aligned were prepared by removing the plastid and mitochondrial genome sequences and any contigs and scaffolds not assembled into pseudomolecules. For barley, the unsplit chromosome version 2 from Ensembl Plants was used. For the allohexaploid wheat, the whole genome sequences were separated into three constituent genomes, A, B, and D, for their separate alignments with the genomes of other species. For oat, the whole genome sequences were separated into individual chromosomes. Multiple whole-genome alignments were conducted using the large-scale genome alignment tool, LASTZ (Harris 2007) and converted to multiple alignment files (MAF) using: axtChain, chainNet, netSyntenic, netToAxt, and axtToMaf (UCSC Genome Browser Toolkit, Anaconda distribution). For barley, wheat, and oat, the alignments were made at the chromosomal level with the genomes of other species. For sunflower, the entire reference genome sequences were aligned with the genomes of other species using Mugsy v1.2.3 (Angiuoli and Salzberg 2011).

Single_cov2.v11 from the MultiZ package (Blanchette et al. 2004) was used to remove any low-scoring alignments where there was overlap in alignments against each reference genome to generate a single coverage alignment across each reference genome. The individual MAF files representing the three separate wheat genomes compared to the reference genomes were combined into a single MAF file for each reference species once single_cov2.v11 completed. ROAST (Hou and Riemer 2008) was used to find orthologous alignments for each reference genome across all related crop genomes and to generate a single MAF file for each reference genome, or for each reference chromosome, in the case of barley, wheat, and oat. For maize, rapa, soybean, and sunflower a copy of the single MAF file was split into multiple MAF files each representing a single chromosome. The resulting individual chromosome MAF files were converted to aligned FASTA format with msa_view. Phylogenetic tree and neutral branch length (estimated from fourfold degenerate sites) analyses were made using PhastCons (msa_view and phyloFit; Siepel et al. 2005) and were used to quantify the constraint intensity at every position across a representative chromosome for each reference genome species. RS scores for each chromosome of each reference genome were calculated with Gerpcol following the same procedures used by Ramu et al. (2017), and the resulting files were concatenated and filtered to remove RS scores ≤ 0. The seven generated RS files with RS scores >0 are accessible as described in **A11** below.

A6. SNP calling and annotation

The following six steps **A6a** to **A6g** were applied to each collection and the three specific paired groups (Table S2), with some exceptions in **A6g.**

A6a. SNP calling

Single nucleotide polymorphisms (SNPs) were called based on BAM files using ANGSD (Korneliussen et al. 2014) with the following parameters: -dovcf 1 -gl 1 -dopost 1 -domajorminor 4 -domaf 1 -snp_pval 1e-6. A VCF file was generated for each collection and each group of samples. Barley split-, wheat halved-, and oat halved-, chromosome VCF files were recombined using a custom Perl script. For each VCF file, the number of SNPs was calculated.

A6b. Annotation using stand-alone Ensembl Variant Effect Predictor (VEP)

All SNPs were annotated based on an ANGSD-generated VCF file using the stand-alone Ensembl VEP (McLaren et al. 2016; Naithani et al. 2017). For barley and wheat, VEP cache databases were downloaded from the Ensembl Plants website, and for the other five species, VEP cache databases were custom-made from the related GTF or GFF files following the instructions of VEP API and software (McLaren et al. 2016). This analysis allowed for the classification of SNPs into 17 different classes, including missense and synonymous variants. We also calculated the proportions of the detected variants associated with loss of function (LOF) based on the total count of variants from seven VEP annotation classes (Splice_acceptor_variant, Splice_donor_variant, Stop_gained, Stop_lost, Start_lost, Splice_region_variant, and Stop_retained_variant).

A6c. Identifying putative deleterious SNPs (dSNPs)

Amino acid substitutions and their effects on protein function were predicted with the SIFT algorithm. Nonsynonymous mutations with SIFT scores <0.05 were defined as putative deleterious mutations. SIFT (<0.05) and GERP++ RS (>0) annotations were combined to identify the deleterious mutations in constrained portions of the genome. These identified dSNP files formed the basis for further analyses below.

A6d. Identifying fixed dSNPs

ANGSD was applied to generate allele frequency estimations (or MAF files) based on dSNP sites that were identified with SIFT and GERP++ RS scores. Based on the allelic frequency data, fixed dSNPs were also identified. Plots were made using custom R (R Core Team, 2019) scripts to assess the distributions of allele frequencies and RS scores with respect to dSNP.

A6e. Expression of identified dSNPs

An effort was made to identify deleterious genes associated with the dSNPs and fixed dSNPs. A deleterious gene in this study was defined as a gene or canonical transcript associated with one or more of the identified dSNPs and they were extracted from the VEP output file based on the identified dSNPs and fixed dSNPs. The expression of these deleterious genes at the stage of 3-leaves in monocots and of the first true leaf in dicots present in the RNA-Seq data was extracted and quantified using a custom Shell script based on StringTie (Pertea et al. 2015). The proportion of the dSNPs associated with expressed deleterious genes was estimated as the count of expressed deleterious genes over all the identified dSNPs. Such estimation represents only the lower bound, as an expressed deleterious gene may harbor more than one dSNP.

A6f. Jackknife estimation of dSNPs

To understand the variation in the detection of dSNPs and fixed dSNPs, we used a jackknife re-sampling approach. The computational effort was considerably large, given the 49 combinations of the seven collections each having seven combinations of collection and groups. Thus, only 10 jackknife sub-samples of size 69 for a collection and of size (group size minus one) for a group were generated, followed by the SNP calling and annotation from **A6a** to **A6d** to estimate dSNPs and fixed dSNPs. The mean and standard deviation of an estimate were calculated from 10 jackknife sub-samples.

A6g. Estimating deleterious base-substitution mutations

We also calculated the deleterious base-substitution mutations per sample (dBSMs) by dividing the detected dSNP count by the reference genome size (Table S5a) and sample size for each collection. This was similarly done for fixed dSNPs for each collection. To assess the variation of these dSNPs or fixed dSNPs, we performed a jackknife sub-sampling, as mentioned above. An effort was also made to calculate the dBSMs per year for the SY group of each collection and estimate the change in dBSMs per year with respect to dSNPs and fixed dSNPs, as this paired group represents the difference in storage years.

A7. Estimating mutation burden

A7a. Estimating mutation burden for each assayed sample

With identified dSNP information, ANGSD was run for genotyping and obtaining deleterious allelic frequency files with respect to each collection and its three specific paired groups. Mutation burden per deleterious locus for individual samples was calculated from sample genotype data based on the numbers of deleterious alleles present in three models: homozygous-mutation burden, heterozygous-mutation burden, and total mutation burden (Ramu et al. 2017; Wang et al. 2017). The homozygous-mutation burden per deleterious locus is the number of derived deleterious alleles in the homozygous state, divided by a product of 2 × total dSNP count. The heterozygous-mutation burden per deleterious locus is the number of derived deleterious alleles existing in the heterozygous state, divided by a product of 2 × total dSNP count. The total mutation burden per deleterious locus is the number of derived deleterious alleles existing in an accession (2 × homozygous-mutation burden + heterozygous-mutation burden), divided by a product of 2 × total dSNP count. A collection or group mutation burden was calculated based on the mean of individual total mutation burdens over all the respective samples.

A7b. Correlating estimated mutation burdens with accession features

An effort was also made to assess if the estimates of three individual mutation burdens for each sample were associated with its accession features such as the year of acquisition, the storage years since the last regeneration, and the germination levels. Linear regressions were performed between mutation burdens and accession features using basic R packages (R Core Team, 2019). Such assessments were made only for the collection samples with or without technical replicate samples. Sample-wise mutation burdens were also plotted against the storage years since the last regeneration.

A8. Inferring adaptive mutation

We applied polyDFE (Tataru et al. 2017) to infer adaptive mutations with the proportion of adaptive substitutions (i.e., those with a selection coefficient greater than zero) by generating the

alpha-dfe value as the ratio of the estimated adaptive substitutions over the observed selected divergence counts (Loewe et al. 2006). The inference to the whole collection was made with only 40 randomly selected samples, as the polyDFE version 2.0 is limited to a sample size of 49 diploid plants. It was based on site frequency spectrum (SFS) data, and such SFS data was obtained by running ANGSD based on synonymous and non-synonymous SNP sites that were annotated as above from the respective VCF file for each collection or group. The ANGSD runs were conducted with -anc option using an ancestral sequence reconstructed (ASR) with two outgroups (Table S5a). The ASR was generated following the steps of **A5c** up to ROAST and using a custom script to filter alignments shorter than 500 to 1000 bp (depending on the crop species; with 1000 bp for wheat, barley, and oat) before the application of FastML (Ashkenazy et al. 2012).

For each polyDFE analysis, the total sequence length of the selected regions (TSLs) was first estimated by the product of the non-synonymous SNP count times the density of SNPs for the species, which was roughly estimated by the total length of the whole reference genome sequences divided by the total SNPs detected from the collection samples in this study (see the step **A6a**). The total sequence length of the neutral regions was estimated following the proportionality principle with 1/3 of the TSLs (Tataru et al. 2017). Such estimation of the sequence length may be conservative with RNA-Seq data and could scale up the alpha-dfe estimation (personal communication with Dr. Thomas Bataillon). Three models (A, C, and D) using the –w option were examined and the resulting alpha-dfe estimates were compared. The final analysis was done with Model A, as it was the most stable model with convergence. Each polyDFE analysis was done with 30 bootstrapped datasets, and the standard deviation of the alpha-dfe estimate was obtained accordingly. Only 30 bootstrapped samples were generated and analyzed, because the effort required for the whole polyDFE analysis was substantial, given the 49 combinations of collection and groups with the seven collections. Also, it is worth noting that our polyDFE analyses did not consider degenerated codon positions as discussed by Bierne and Eyre-Walker (2003), as the required effort was too large for our large genome datasets (Table S5a). Thus, our estimates of synonymous and non-synonymous SNP sites were not accurate and can bias the alpha-dfe estimation.

A9. Testing significance

Efforts were made to assess the significance of the difference in an estimate between paired groups. We applied a non-parametric Kruskal-Wallis test (Kruskal and Wallis 1952) to assess the difference in median value conservatively for each paired group in each collection for the following estimates: the counts and proportions of dSNPs and fixed dSNPs, individual mutation burdens, and the 30 bootstrapped alpha-dfe estimates for adaptive mutations.

A10. Gene ontology (GO) analysis of deleterious genes

An effort was also made to do GO analysis of deleterious genes identified for the SY group pair of each collection to identify the common and unique GO features. Genes or canonical transcripts associated with the identified dSNPs were extracted from the VEP output file. The extracted genes were analyzed by Blast2GO Pro v.5.2.5 (Conesa and Götz 2008) using the Gene Ontology Annotation workflow (BLAST, mapping, and annotation) and Enrichment Analysis (Fisher Exact Test). Non-redundant GO term sets were visualized using REVIGO (Supek et al. 2011) with treemaps and tag clouds to assist biological interpretation. These GO analyses were

also conducted for genes associated with fixed dSNPs and with respect to common and unique genes in the SY group pair.

A11. Data and code availability

Custom Perl, Shell or related pipelines that we generated for the bioinformatics analyses of these RNA-Seq data and R scripts for the generation of all figures will be available upon request to the first corresponding author. Acquired original RNA-Seq data (Table S1) was deposited in NCBI's SRA database. The following sets of supplemental output or meta data for each species (dSNP annotation, SIFT and GERP++RS scores) were deposited into Figshare (https://doi.org/10.6084/m9.figshare.12234431):

1. barley-del-SNPs.txt
2. wheat-del-SNPs.txt
3. oat-del-SNPs.txt
4. soybean-del-SNPs.txt
5. maize-del-SNPs.txt
6. rapa-del-SNPs.txt
7. sunflower-del-SNPs.txt
8. barley-RS-scores.gtzero.txt.gz (12 MB)
9. wheat-RS-scores.gtzero.txt.gz (88 MB)
10. oat-RS-scores.gtzero.txt.gz (151 MB)
11. soybean-RS-scores.gtzero.txt.gz (10 MB)
12. maize-RS-scores.gtzero.txt.gz (17 MB)
13. rapa-RS-scores.gtzero.txt.gz (9 MB)
14. sunflower-RS-scores.gtzero.txt.gz (7 MB)
15. oat-siftdb.zip (5 GB)
16. soybean-siftdb.zip (3 GB)
17. maize-siftdb.zip (2 GB)
18. rapa-siftdb.zip (1.1 GB)
19. sunflower-siftdb.zip (1.6 GB)

B. Summary results on variant identification and annotation in seven collections

The RNA-Seq analysis of 490 plant samples of the seven collections (Tables S1) generated a range of 4.6 to 18.0 million mapped sequence reads (MSR) per assayed sample with a mean of 11.1 million MSR (Table S3). Generally, the percentages of mapped reads in bam files were lower in outcrossing, than selfing, crops. Calling variants including SNP using ANGSD identified a range of 0.51 to 2.94 million SNPs for the seven collections with a mean of 1.56 million SNPs (Table S4). SNP annotation using Ensembl-Variant Effect Predictor allowed for the classification of SNPs into 17 different classes (Table S4). Most SNPs were located at intergenic, upstream, and downstream genic regions. The annotation also revealed abundant intron variants, indicating the presence of substantial unspliced pre-mRNAs in the sequence reads. The proportions of missense SNPs over all detected SNPs ranged from 0.122 (sunflower) to 0.210 (rapa) with a mean of 0.159. The proportions of synonymous SNPs ranged from 0.126 (barley) to 0.401 (rapa) with an average of 0.193. More missense than synonymous variants were detected in barley, oat, and soybean collections (Table S4). We also assessed the proportions of the detected variants associated with loss of function (LOF) based on the total count of variants from seven VEP annotation classes (Splice acceptor variant, Splice donor variant, Stop gained,

Stop lost, Start lost, Splice region variant and Stop retained variant). The proportions of LOF variants varied for these collections from 0.031 (wheat and oat) to 0.084 (soybean) with a mean of 0.027 (Table S4).

C. References for materials and methods

Angiuoli SV, Salzberg SL (2011) Mugsy: fast multiple alignment of closely related whole genomes. *Bioinformatics* 27:334–342.

AOSA (2013) Association of Official Seed Analysts Rules for Testing Seeds. Moline, IL, USA

Ashkenazy H, et al. (2012) FastML: a web server for probabilistic reconstruction of ancestral sequences. *Nucleic Acids Res* 40:W580–4.

Bierne N, Eyre-Walker A (2003) The problem of counting sites in the estimation of the synonymous and nonsynonymous substitution rates: implications for the correlation between the synonymous substitution rate and codon usage bias. *Genetics* **165**:1587–1597.

Blanchette M, et al. (2004) Aligning multiple genomic sequences with the threaded blockset aligner. *Genome Res* 14:708–715.

Bolger AM, Lohse M, Usadel B (2014) Trimmomatic: A flexible trimmer for Illumina sequence data. *Bioinformatics* 30:2114-2120.

Conesa A, Götz S (2008) Blast2GO: A comprehensive suite for functional analysis in plant genomics. *Int J Plant Genomics*. vol. 2008, Article ID 619832.

Davydov EV, et al. (2010) Identifying a high fraction of the human genome to be under selective constraint using GERP++. *PLoS Comput Biol* 6:e1001025.

Dunn OJ (1964) Multiple comparisons using rank sums. *Technometrics* 6:241–252.

FAO, Genebank standards for plant genetic resources for food and agriculture. Rev. ed. (FAO, Rome, Italy, 2014).

Harris RS (2007) Improved pairwise alignment of genomic DNA. Ph.D. Thesis, The Pennsylvania State University.

Hou M, Riemer C (2008) TOAST and ROAST. http://www.bx.psu.edu/~cathy/toast-roast.tmp/README.toast-roast.html [accessed: 2023-10-19].

ISTA (2009) International Seed Testing Association Handbook on Seedling Evaluation – 3rd Edition. Bassersdorf, Switzerland.

IWGSC et al. (2018) Shifting the limits in wheat research and breeding using a fully annotated reference genome, *Science* 361:eaar7191

Kamal N, et al. (2022) The mosaic oat genome gives insights into a uniquely healthy cereal crop. *Nature* 606:113–119.

Korneliussen TS, Albrechtsen A, Nielsen R (2014) ANGSD: analysis of next generation sequencing data. *BMC Bioinformatics* 15:356.

Kruskal WH, Wallis WA (1952) Use of ranks in one-criterion variance analysis. *J Am Stat Assoc* 47:583–621.

Li H, Durbin R (2010) Fast and accurate long-read alignment with Burrows-Wheeler transform. *Bioinformatics* 26:589-95.

Li H, et al. (2009) The Sequence Alignment/Map format and SAMtools. *Bioinformatics* 16:2078-9.

Loewe L, Charlesworth B, Bartolomé C, Nöel V (2006) Estimating selection on nonsynonymous mutations. *Genetics* 172:1079–1092.

Mascher M, et al. (2017) A chromosome conformation capture ordered sequence of the barley genome. *Nature* 544:427-433.

McLaren W, et al. (2016) The Ensembl variant effect predictor. *Genome Biol* 17:122.

Naithani S, Geniza M, Jaiswal P (2017) Variant effect prediction analysis using resources available at Gramene Database. *Methods Mol Biol* 1533:279-297.

Pertea M, et al. (2015) StringTie enables improved reconstruction of a transcriptome from RNA-Seq reads. *Nature Biotech* 33:290-295.

R Core Team (2019) R: A language and environment for statistical computing. R Foundation for Statistical Computing, Vienna, Austria. ISBN 3-900051-07-0, URL http://www.R-project.org.

Ramu P, et al. (2017) Cassava haplotype map highlights fixation of deleterious mutations during clonal propagation. *Nat Genet* 49:959-963.

Siepel A, et al. (2005) Evolutionarily conserved elements in vertebrate, insect, worm, and yeast genomes. *Genome Res* 15:1034-1050.

Supek F, Bošnjak M, Škunca N, Šmuc T (2011) REVIGO summarizes and visualizes long lists of gene ontology terms. *PLoS One* 6:e21800.

Tataru P, Mollion M, Glémin S, Bataillon T (2017) Inference of distribution of fitness effects and proportion of adaptive substitutions from polymorphism data. *Genetics* 207:1103–1119.

Van der Auwera GA, et al. (2013) From FastQ data to high confidence variant calls: the Genome Analysis Toolkit best practices pipeline. *Curr Protoc Bioinformatics* 43:11.10.1–11.10.33.

Vaser R, Adusumalli S, Leng SN, Sikic M, Ng PC (2015) SIFT missense predictions for genomes. *Nat Protoc* 11:1:1-9.

Wang L, et al. (2017) The interplay of demography and selection during maize domestication and expansion. *Genome Biol* 18:215.

D: Tables S1 to S11

Table S1. Summary of all plant materials used for this study, including RNA-Seq sequencing and sequence deposition. Additional inventory and sequence information on the materials can be found in Tables S2 and S3, respectively. The assayed samples were acquired from the corresponding germplasm collections held at the Plant Gene Resources of Canada (PGRC), Saskatoon, Saskatchewan, Canada.

Collection	Collection size	Sample size	RNA-Seq	NCBI's BioProject accession number
Barley	40740	70 samples +2 technical reps	24 samples per HiSeq 2500 lane x 3 lanes	PRJNA543133
Oat	28515	70 samples +2 technical reps	24 samples per HiSeq 2500 lane x 3 lanes	PRJNA942217
Wheat	11055	70 samples +2 technical reps	24 samples per HiSeq 2500 lane x 3 lanes	PRJNA543139
Soybean	1119	70 samples +2 technical reps	24 samples per HiSeq 2500 lane x 3 lanes	PRJNA543137
Maize	615	70 samples +2 technical reps	24 samples per HiSeq 2500 lane x 3 lanes	PRJNA543135
Rapa	747	70 samples +2 technical reps	24 samples per HiSeq 2500 lane x 3 lanes	PRJNA543136
Sunflower	581	70 samples +2 technical reps	24 samples per HiSeq 2500 lane x 3 lanes	PRJNA543138

Table S1a. The relevant biological information for the seven crops studied.

Crop	Species	Taxonomic group	Mating[a]	Ploidy	Genome size[b]	Gene number[b]
Barley	*Hordeum vulgare* L.	Monocot	Selfing	2n=2x=14	5.3 Gb	81687
Oat	*Avena sativa* L.	Monocot	Selfing	2n=6x=42	11 Gb	80608
Wheat	*Triticum aestivum* L.	Monocot	Selfing	2n=6x=42	17 Gb	107,891
Soybean	*Glycine max* (L.) Merr.	Eudicot	Selfing	2n=4x=40	1.1 Gb	55897
Maize	*Zea mays* L.	Monocot	Outcrossing	2n=2x=20	4.8 Gb	39756
Rapa	*Brassica rapa* L.	Eudicot	Outcrossing	2n=2x=20	346 Mb	43129
Sunflower	*Helianthus annuus* L.	Eudicot	Outcrossing	2n=2x=34	3.6 Gb	70864

[a] These four selfing crops could have up to 3% outcrossing when growing in a field

[b] Estimates of haploid genome size and gene number were obtained from https://plants.ensembl.org/species.html (assessed on October 6 2023)

Table S2a. List of inventory and RNA-Seq information for 72 assayed barley samples representing their respective PGRC collection, along with the composition of three paired groups for group analysis. CN=PGRC accession number, Country=country of origin, YA=year acquired, YR=year last regenerated, NR=the number of field regenerations done since the acquisition, GR=germination rate, SY=grouping based on the year last regenerated, RF=grouping based on NR, GD=grouping based on GR, and SEQ-Name=sequence file name.

Barley						Group composition			Sample
CN	Country	YA	YR	NR	GR	SY	RF	GD	SEQ-Name
81789	Chile	1979	2009	1	88	10	1		CN081789_S1
58854	Mongolia	1924	2000	2	81		2		CN058854_S2
74861	Bosnia and Herzeg.	1972	2009	1	91	10	1	91	CN074861_S3
74403	Switzerland	1971	2008	2	97		2	91	CN074403_S4
75883	Macedonia	1973	2009	1	89	10	1		CN075883_S5
69054	Romania	1968	2009	1	88	10	1		CN069054_S6
69156	Uruguay	1968	2009	1	75		1		CN069156_S7
43596	China	1986	1995	4	62			68	CN043596_S8
83382	Brazil	1982	2009	1	85	10	1		CN083382_S9
74798	Lithuania	1972	2009	1	84	10	1		CN074798_S10
33367	Canada	1976	1995	1	68		1	68	CN033367_S11
88403	Algeria	1923	2000	2	82				CN088403_S12
59701	Egypt	1940	2002	2	81		2		CN059701_S13
31049	USA	1976	1994	1	34		1	68	CN031049_S14
231	Germany	1974	1992	3	85	27			CN000231_S15
172	Canada	1973	1987	4	63	27		68	CN000172_S16
12988	Canada	1983	1990	2	74	27	2		CN012988_S17
290	USA	1974	1985	5	79	27			CN000290_S18
39732	Canada	1982	1982	0	12	27		68	CN039732_S19
89110	Eritrea	1961	2005	2	91		2		CN089110_S20
89156	Italy	1962	2005	2	91		2		CN089156_S21
252	USA	1974	1992	5	84	27			CN000252_S22
90066	Bosnia and Herzeg.	1969	2006	2	85				CN090066_S23
58689	Egypt	1923	2000	2	88		2		CN058689_S24
58963	Venezuela	1925	2000	2	64		2	68	CN058963_S25
59461	Argentina	1936	2000	2	90		2		CN059461_S26
59577	Portugal	1938	2002	2	96		2	91	CN059577_S27
60340	Ethiopia	1930	2004	2	86		2		CN060340_S28
83473	Bolivia	1983	2009	1	77	10	1		CN083473_S29
296	USA	1974	1983	3	91	27		91	CN000296_S30
78926	Ecuador	1974	2009	1	89	10	1		CN078926_S31
80762	Bolivia	1976	2009	1	77	10	1		CN080762_S32
89775	Tajikistan	1968	2006	2	84		2		CN089775_S33
279	USA	1974	1983	3	88	27			CN000279_S34
77017	Ethiopia	1973	2008	2	66			68	CN077017_S35
199	USA	1973	1983	5	71	27			CN000199_S36
38783	Germany	1981	1994	1	27		1	68	CN038783_S37
89912	Ukraine	1968	2006	2	93		2	91	CN089912_S38
3843	Korea, South	1980	1989	3	5	27		68	CN003843_S39
58516	Venezuela	1917	1999	2	86		2		CN058516_S40
60784	Germany	1962	2005	2	95		2	91	CN060784_S41
44439	Canada	1987	1987	0	46	27		68	CN044439_S42
4072	Canada	1980	1994	3	77				CN004072_S43
39283	USA	1982	1995	1	78		1		CN039283_S44
83925	New Zealand	1985	2011	2	97	10	2	91	CN083925_S45
29180	USA	1989	1994	2	40			68	CN029180_S46
189	Canada	1973	1994	8	84				CN000189_S47
39732_R1	Canada	1982	1982	0	12				CN039732X1_S48
12	Canada	1972	1984	3	80	27			CN000012_S49
43386	Canada	1985	1985	0	39	27		68	CN043386_S50
80492	Pakistan	1976	2009	1	92	10	1	91	CN080492_S51
68434	Italy	1968	2009	2	94		2	91	CN068434_S52
74807	Kyrgyzstan	1972	2009	1	96	10	1	91	CN074807_S53
58544	Italy	1922	1999	2	92		2	91	CN058544_S54
35142	India	1977	1994	1	92		1	91	CN035142_S55
81927	Spain	1980	2009	1	73	10	1		CN081927_S56
82033	Spain	1980	2009	1	94	10	1	91	CN082033_S57
60487	South Africa	1955	2004	2	80		2		CN060487_S58
39017	Canada	1982	1992	1	41	27	1	68	CN039017_S59
80080	Belarus	1975	2010	1	81	10	1		CN080080_S60
45934	China	1988	1994	1	96		1	91	CN045934_S61
69320	Korea, North	1968	2010	2	86	10	2		CN069320_S62
39337	Canada	1982	1982	0	40	27		68	CN039337_S63
38780	Germany	1981	1994	1	4		1	68	CN038780_S64
38782	Germany	1981	1994	1	7		1	68	CN038782_S65
2	Canada	1972	1985	3	61	27		68	CN000002_S66
88430	India	1923	2000	2	92		2	91	CN088430_S67
89697	Slovenia	1967	2006	2	99		2	91	CN089697_S68
89814	Ukraine	1968	2006	2	91				CN089814_S69
90068	Bosnia and Herzeg.	1969	2006	2	91		2		CN090068_S70
90767	Croatia	1967	2006	2	99		2	91	CN090767_S71
39732_R2	Canada	1982	1982	0	12				CN039732X2_S72

Table S2b. List of inventory and RNA-Seq information for 72 assayed wheat samples representing their respective PGRC collection, along with the composition of three paired groups for group analysis. CN=PGRC accession number, Country=country of origin, YA=year acquired, YR=year last regenerated, NR=the number of field regenerations done since the acquisition, GR=germination rate, SY=grouping based on the year last regenerated, RF=grouping based on NR, GD=grouping based on GR, and SEQ-Name=sequence file name. Note NA is not available.

Wheat						Group composition			Sample
CN	Country	YA	YR	NR	GR	SY	RF	GD	SEQ-Name
1686	UK	1977	1995	2	90		2	72	CN001686_S1
1693	Canada	1977	1995	2	27		2		CN001693_S2
1694	Canada	1977	1995	2	53		2		CN001694_S3
2657	Greece	1979	1996	4	78			72	CN002657_S4
2698	Portugal	1979	1995	2	81		2	72	CN002698_S5
2707	USA	1979	1996	3	93			72	CN002707_S6
5231	Canada	1980	1995	3	87			72	CN005231_S7
5262	Iran	1980	1996	5	65				CN005262_S8
5815	Turkey	1980	1999	5	82	20		72	CN005815_S9
5823	Canada	1980	1998	3	80			72	CN005823_S10
5945	Iran	1978	1997	5	42				CN005945_S11
6124	Ethiopia	1981	1996	2	74		2	72	CN006124_S12
6869	Russia	1981	1995	2	63		2		CN006869_S13
7189	Russia	1981	1995	1	72			72	CN007189_S14
7228	Russia	1981	1995	2	33		2		CN007228_S15
9783	Australia	1980	1993	1	58	25	1		CN009783_S16
9821	Hungary	1980	1993	1	84	25		72	CN009821_S17
10157	Israel	1980	1993	1	34	25	1		CN010157_S18
10960	Bolivia	1980	1993	1	57	25			CN010960_S19
11045	Algeria	1980	1993	1	62	25	1		CN011045_S20
11061	Canada	1980	2008	3	66	20			CN011061_S21
11150	Russia	1980	1993	1	77	25		72	CN011150_S22
11189	Canada	1980	2008	2	94	20	2	72	CN011189_S23
11993	Canada	1980	1995	1	59		1		CN011993_S24
11995	Canada	1980	1993	1	92			72	CN011995_S25
12124	India	1980	1993	1	11	25	1	26	CN012124_S26
12349	Peru	1980	1995	1	54				CN012349_S27
29758	China	1974	1995	2	52		2		CN029758_S28
29767	China	1974	1996	3	84			72	CN029767_S29
30240	Pakistan	1975	1995	6	12			26	CN030240_S30
30279	Pakistan	1975	1995	4	34				CN030279_S31
30294	Pakistan	1975	1995	5	47				CN030294_S32
30302	Pakistan	1975	2000	6	62	20			CN030302_S33
30303	Pakistan	1975	2000	5	37	20			CN030303_S34
30614	Pakistan	1976	1995	3	22			26	CN030614_S35
32480	Australia	1978	1996	3	52				CN032480_S36
32569	Russia	1978	1981	1	1	25	1	26	CN032569_S37
39669	Russia	1982	2006	2	96	20	2	72	CN039669_S38
40652	Kenya	1983	2000	2	55	20	2		CN040652_S39
40693	Kenya	1983	2000	2	69	20	2		CN040693_S40
40719	Kenya	1983	2000	2	56	20	2		CN040719_S41
40736	Kenya	1983	2000	2	92	20	2	72	CN040736_S42
40791	Kenya	1983	2000	2	60	20	2		CN040791_S43
40828	Kenya	1983	1995	1	17			26	CN040828_S44
40829	Kenya	1983	1995	1	23		1	26	CN040829_S45
40830	Kenya	1983	1995	1	31				CN040830_S46
40831	Kenya	1983	1995	1	29		1		CN040831_S47
001694_R1	Canada	1977	1995	2	53		2		CN001694X1_S48
40855	Kenya	1983	1995	1	22			26	CN040855_S49
40857	Kenya	1983	1995	1	20		1	26	CN040857_S50
40864	Kenya	1983	1995	1	12		1	26	CN040864_S51
40865	Kenya	1983	1995	1	24			26	CN040865_S52
40868	Kenya	1983	1995	1	4		1	26	CN040868_S53
40874	Kenya	1983	1995	1	14			26	CN040874_S54
40906	Kenya	1983	1995	1	38		1		CN040906_S55
42510	Brazil	1984	1995	1	13			26	CN042510_S56
42511	Brazil	1984	1995	1	15			26	CN042511_S57
42519	Brazil	1984	1995	1	26		1	26	CN042519_S58
42528	Canada	1984	1995	1	28		1		CN042528_S59
43843	Canada	1986	1994	1	37				CN043843_S60
43980	Canada	1985	1995	1	44				CN043980_S61
44047	Australia	1985	1994	1	48	25	1		CN044047_S62
44051	Australia	1985	1996	2	51		2		CN044051_S63
44392	USA	1985	1994	1	18	25		26	CN044392_S64
44409	USA	1985	1994	1	43	25	1		CN044409_S65
44427	USA	1985	1994	1	29				CN044427_S66
45577	Mexico	1985	1994	1	68		1		CN045577_S67
45594	Mexico	1985	1994	1	35				CN045594_S68
45604	Mexico	1985	1994	1	32	25			CN045604_S69
51260	Australia	1989	1995	1	3		1	26	CN051260_S70
106318	Canada	1998	2012	NA	76	20		72	CN106318_S71
001694_R2	Canada	1977	1995	2	53		2		CN001694X2_S72

Table S2c. List of inventory and RNA-Seq information for 72 assayed oat samples representing their respective PGRC collection, along with the composition of three paired groups for group analysis. CN=PGRC accession number, Country=country of origin, YA=year acquired, YR=year last regenerated, NR=the number of field regenerations done since the acquisition, GR=germination rate, SY=grouping based on the year last regenerated, RF=grouping based on NR, GD=grouping based on GR, and SEQ-Name=sequence file name.

Oat						Group composition			Sample
CN	Country	YA	YR	NR	GR	SY	RF	GD	SEQ-Name
3161	Libya	1980	2001	7	100			96	CN003161_S1
4395	Russia	1980	1993	5	90	21			CN004395_S2
4873	Georgia	1976	1999	9	84			84	CN004873_S3
5187	Canada	1980	1998	4	88	21			CN005187_S4
21346	Turkey	1990	2016	5	96	16		96	CN021346_S5
21953	Ethiopia	1990	2016	4	92	16			CN021953_S6
25369	Spain	1990	2000	4	86				CN025369_S7
43863	Canada	1986	1999	2	80			84	CN043863_S8
51320	Bulgaria	1990	2002	1	30		1	84	CN051320_S9
53565	Sweden	1989	1989	1	86	21	1		CN053565_S10
53617	China	1989	2015	3	94	16			CN053617_S11
53840	United States	1989	1989	1	64	21	1	84	CN053840_S12
54228	Germany	1938	1999	2	74		2	84	CN054228_S13
54851	United Kingdom	1989	2000	2	86				CN054851_S14
63442	Netherlands	1989	2001	2	86				CN063442_S15
63751	Yugoslavia	1989	2001	2	90				CN063751_S16
63914	Ireland	1989	2001	2	84		2	84	CN063914_S17
63916	Ethiopia	1951	2001	2	96			96	CN063916_S18
63969	Norway	1989	2007	3	96	16		96	CN063969_S19
64317	Mongolia	1989	2015	3	96	16		96	CN064317_S20
64431	Estonia	1989	2001	2	98				CN064431_S21
64654	Pakistan	1960	2001	2	88				CN064654_S22
64947	Nepal	1963	2001	2	86				CN064947_S23
65015	Greece	1963	2001	2	70		2	84	CN065015_S24
65073	Portugal	1963	2001	2	90				CN065073_S25
65157	Albania	1963	2001	2	100			96	CN065157_S26
67324	Slovenia	1967	2001	2	96				CN067324_S27
72565	Brazil	1970	2001	2	88				CN072565_S28
74849	Bosnia and Herzeg.	1972	2001	2	100			96	CN074849_S29
79297	Ecuador	1975	2001	2	82			84	CN079297_S30
79327	France	1975	2001	2	98				CN079327_S31
79332	Poland	1975	2015	3	96	16			CN079332_S32
80895	Macedonia	1977	2003	4	96	16		96	CN080895_S33
81704	Chile	1979	2002	3	54			84	CN081704_S34
81756	Chile	1979	2014	3	98	16		96	CN081756_S35
88377	Kenya	1989	1999	2	78		2	84	CN088377_S36
88392	Tunisia	1989	1999	2	46		2	84	CN088392_S37
88434	New Zealand	1989	1999	2	36			84	CN088434_S38
88457	Uzbekistan	1989	1989	1	92	21	1		CN088457_S39
88459	Kyrgyzstan	1989	1989	1	88	21	1		CN088459_S40
88611	Hungary	1989	1999	2	96		2	96	CN088611_S41
88662	Uruguay	1989	1999	2	90				CN088662_S42
88678	France	1989	1999	2	98			96	CN088678_S43
88707	Peru	1936	1990	1	92	21	1		CN088707_S44
88739	Sweden	1989	1999	2	100			96	CN088739_S45
88747	Slovakia	1989	1990	1	98	21	1	96	CN088747_S46
88757	South Africa	1989	1999	2	94				CN088757_S47
63442_R1	Netherlands	1989	2001	2	86				CN063442X1_S48
88758	Finland	1989	1999	2	98		2		CN088758_S49
88767	Latvia	1939	1999	2	82		2	84	CN088767_S50
88828	Israel	1989	2000	2	80			84	CN088828_S51
88832	Cyprus	1989	2000	2	86				CN088832_S52
88842	Mongolia	1989	2000	2	92				CN088842_S53
88844	Ukraine	1989	2000	2	88				CN088844_S54
88854	Iran	1989	2000	2	94				CN088854_S55
88917	Argentina	1948	2000	2	94				CN088917_S56
88968	Turkey	1989	2000	2	92				CN088968_S57
90177	Poland	1989	1999	2	82			84	CN090177_S58
90178	Algeria	1989	1999	2	96			96	CN090178_S59
90182	China	1989	2003	4	94	16			CN090182_S60
90190	Australia	1989	1999	2	88				CN090190_S61
90205	Spain	1989	1989	1	24	21	1	84	CN090205_S62
90299	Zimbabwe	1989	1999	2	90				CN090299_S63
90336	Korea, North	1989	1999	2	94				CN090336_S64
90364	United Kingdom	1932	2009	3	98	16			CN090364_S65
90395	Japan	1989	1990	1	94	21	1		CN090395_S66
90447	Mexico	1947	2000	2	98		2	96	CN090447_S67
90469	Netherlands	1989	2000	2	96			96	CN090469_S68
90470	Denmark	1948	2000	2	98				CN090470_S69
90477	India	1989	2000	2	88				CN090477_S70
99001	United States	1989	2001	3	92				CN099001_S71
63442_R2	Netherlands	1989	2001	2	86				CN063442X2_S72

Table S2d. List of inventory and RNA-Seq information for 72 assayed soybean samples representing their respective PGRC collection, along with the composition of three paired groups for group analysis. CN=PGRC accession number, Country=country of origin, YA=year acquired, YR=year last regenerated, NR=the number of field regenerations done since the acquisition, GR=germination rate, SY=grouping based on the year last regenerated, RF=grouping based on NR, GD=grouping based on GR, and SEQ-Name=sequence file name.

Soybean						Group composition			Sample
CN	Country	YA	YR	NR	GR	SY	RF	GD	SEQ-Name
29747	China	1974	2001	3	85				CN029747_S1
29793	China	1974	2001	3	55				CN029793_S2
30316	China	1975	2001	3	79				CN030316_S3
30318	China	1975	2001	3	58				CN030318_S4
31719	Netherlands	1977	2001	3	49			49	CN031719_S5
31984	Norway	1977	2016	4	98	10		86	CN031984_S6
32354	Hungary	1978	2000	2	44	20	2	49	CN032354_S7
32634	UK	1978	2006	2	76				CN032634_S8
32766	Poland	1978	2006	2	86		2	86	CN032766_S9
33248	Canada	1973	2008	6	80	10			CN033248_S10
33253	Canada	1973	2011	3	94	10		86	CN033253_S11
33255	Canada	1973	2014	3	93	10		86	CN033255_S12
33257	Canada	1974	2010	3	83	10			CN033257_S13
33259	Canada	1974	2001	3	80				CN033259_S14
33267	Canada	1975	2001	2	54		2		CN033267_S15
33279	Canada	1977	2000	2	41	20		49	CN033279_S16
33908	China	1978	2013	2	87	10	2	86	CN033908_S17
35756	Russia	1979	2006	1	53		1		CN035756_S18
35920	Russia	1979	2005	2	85		2		CN035920_S19
36009	China	1979	2001	1	40		1	49	CN036009_S20
36215	China	1980	2006	2	57				CN036215_S21
36333	Russia	1980	2006	2	72				CN036333_S22
36337	Japan	1980	2006	2	68		2		CN036337_S23
36342	Germany	1980	2005	1	77		1		CN036342_S24
39077	Canada	1982	2000	3	91	20		86	CN039077_S25
39150	Canada	1982	2000	1	77	20			CN039150_S26
39153	Canada	1982	2000	2	69	20	2		CN039153_S27
39178	Canada	1982	2001	2	14			49	CN039178_S28
39193	Canada	1982	2001	2	53		2		CN039193_S29
42220	Canada	1983	2014	1	97	10	1	86	CN042220_S30
42387	Canada	1983	2014	1	98	10		86	CN042387_S31
42388	Canada	1983	2014	1	94	10	1	86	CN042388_S32
42536	China	1984	2003	1	19		1	49	CN042536_S33
42928	USA	1984	2014	1	95	10		86	CN042928_S34
45087	Ukraine	1987	2003	1	29		1	49	CN045087_S35
45092	France	1987	2006	2	65				CN045092_S36
45107	Russia	1987	2006	5	92			86	CN045107_S37
51375	Japan	1991	2014	1	85	10			CN051375_S38
51844	Canada	1988	2013	2	93	10	2	86	CN051844_S39
51845	Canada	1988	2014	1	96	10	1	86	CN051845_S40
52635	Russia	1994	2002	1	58		1		CN052635_S41
52644	Russia	1994	2002	1	18			49	CN052644_S42
52648	Russia	1994	2015	1	95	10	1	86	CN052648_S43
52653	Russia	1994	2015	1	77	10			CN052653_S44
52740	Canada	1995	2002	1	19		1	49	CN052740_S45
52873	Romania	1978	2006	2	55		2		CN052873_S46
107352	Unknown	1997	1997	0	34	20		49	CN107352_S47
32634_R1	UK	1978	2006	2	76				CN032634X1_S48
107360	Unknown	1997	1997	0	59	20			CN107360_S49
107370	Unknown	1997	1997	0	61	20			CN107370_S50
107373	Canada	1998	1998	0	56	20			CN107373_S51
107385	Canada	1998	1998	0	38	20		49	CN107385_S52
107406	Canada	1998	1998	0	76	20			CN107406_S53
107407	Canada	1998	1998	0	67	20			CN107407_S54
107420	Canada	2002	2002	0	19			49	CN107420_S55
107421	Canada	1999	1999	0	35	20		49	CN107421_S56
107422	Unknown	1999	1999	0	44	20		49	CN107422_S57
107436	Canada	2000	2000	0	75	20			CN107436_S58
107449	Japan	2002	2002	0	69				CN107449_S59
107450	Canada	2003	2003	0	51				CN107450_S60
107459	Canada	2004	2004	0	55				CN107459_S61
107472	Netherlands	1976	2004	2	69		2		CN107472_S62
107515	France	1976	2004	2	60		2		CN107515_S63
107563	Hungary	1976	2004	2	78				CN107563_S64
107570	China	1976	2003	1	73		1		CN107570_S65
107574	Japan	1976	2003	1	72				CN107574_S66
107614	Germany	1976	2003	1	73		1		CN107614_S67
107652	Japan	1976	2004	2	89		2	86	CN107652_S68
107844	Belgium	1976	2005	2	52				CN107844_S69
107865	Serbia	1976	2005	2	67				CN107865_S70
107880	Korea	1976	2005	1	23		1	49	CN107880_S71
32634_R2	UK	1978	2006	2	76		2		CN032634X2_S72

Table S2e. List of inventory and RNA-Seq information for 72 assayed maize samples representing their respective PGRC collection, along with the composition of three paired groups for group analysis. CN=PGRC accession number, Country=country of origin, YA=year acquired, YR=year last regenerated, NR=the number of field regenerations done since the acquisition, GR=germination rate, SY=grouping based on the year last regenerated, RF=grouping based on NR, GD=grouping based on GR, and SEQ-Name=sequence file name.

Maize						Group composition			Sample
CN	Country	YA	YR	NR	GR	SY	RF	GD	SEQ-Name
18928	Canada	1995	1995	0	96			94	CN18928_S1
29710	China	1974	1989	3	86	29			CN29710_S2
29717	China	1974	NA	3	89				CN29717_S3
30557	Russia	1975	1991	2	85		2		CN30557_S4
33926	China	1978	2011	3	96			94	CN33926_S5
35616	Canada	1979	2011	6	84				CN35616_S6
35617	Canada	1979	2011	5	99			94	CN35617_S7
35622	Canada	1979	1994	5	78				CN35622_S8
35623	Canada	1979	1994	5	86				CN35623_S9
35866	Canada	1979	2011	2	86		2		CN35866_S10
35947	Canada	1979	1994	5	85				CN35947_S11
36013	China	1979	1987	1	88	29	1		CN36013_S12
36237	Canada	1980	2012	9	96	7		94	CN36237_S13
36260	Canada	1980	1987	1	69	29	1	70	CN36260_S14
36262	Canada	1980	1987	1	87	29	1		CN36262_S15
36283	Canada	1980	1992	2	84				CN36283_S16
36286	Canada	1980	1992	2	70		2	70	CN36286_S17
36295	Canada	1980	2012	1	40	7	1	70	CN36295i101_S18
36800	Russia	1980	2014	2	77	7			CN36800_S19
36804	USA	1980	2014	2	98	7	2	94	CN36804_S20
36825	Canada	1980	2014	2	93	7	2		CN36825_S21
36853	Turkey	1980	2015	2	91	7			CN36853_S22
36885	Canada	1980	2015	2	80	7	2		CN36885_S23
36886	Russia	1980	2015	2	70	7	2	70	CN36886_S24
36889	USA	1980	2015	3	90	7			CN36889_S25
36890	USA	1980	2015	2	89	7			CN36890_S26
36931	USA	1980	2016	2	78	7	2		CN36931_S27
36934	USA	1980	2016	3	80	7			CN36934_S28
36945	Austria	1980	2016	2	95	7		94	CN36945_S29
36952	Hungary	1980	2016	2	99	7	2	94	CN36952_S30
36971	Turkey	1980	2017	2	70	7	2	70	CN36971_S31
36973	USA	1980	2017	2	82	7	2		CN36973_S32
36982	Israel	1980	2017	2	86	7			CN36982_S33
36997	Czech Rep.	1980	2017	6	94	7		94	CN36997_S34
37003	Czech Rep.	1980	2009	5	86				CN37003_S35
37038	France	1980	1980	0	23	29		70	CN37038_S36
37040	Canada	1980	1984	1	31	29	1	70	CN37040_S37
37054	Germany	1980	1984	1	67	29	1	70	CN37054_S38
37063	Germany	1980	2009	2	86		2		CN37063_S39
37065	Germany	1980	2009	2	80				CN37065_S40
37071	Netherlands	1980	2009	2	85		2		CN37071_S41
37080	Hungary	1980	2009	2	90				CN37080_S42
37089	France	1980	1985	1	26	29	1	70	CN37089_S43
37100	Canada	1980	1986	1	81	29	1		CN37100_S44
37115	Germany	1980	2009	2	80		2		CN37115_S45
37117	Germany	1980	2009	2	100			94	CN37117_S46
37134	Czech Rep.	1980	1987	1	20	29	1	70	CN37134_S47
36295_R1	Canada	1980	2012	1	40		1		CN36295i101x1_S48
37488	France	1981	2009	2	98		2	94	CN37488_S49
38968	Canada	1981	2009	7	92				CN38968_S50-QD
38981	Canada	1981	2009	4	96			94	CN38981_S51
38998	Canada	1981	2009	5	92				CN38998_S52
40135	Canada	1983	1989	1	66	29	1	70	CN40135_S53
40137	Canada	1983	1994	3	87				CN40137_S54
40140	Canada	1983	1994	3	90				CN40140_S55
40162	Canada	1983	2010	3	100			94	CN40162_S56
40466	Canada	1983	2010	2	98		2	94	CN40466_S57
40485	France	1983	2010	3	84				CN40485_S58
41083	France	1983	2010	3	92				CN41083_S59
42430	Russia	1984	1989	1	90	29	1		CN42430_S60
42446	Russia	1984	1990	2	88	29			CN42446_S61
42449	Russia	1984	1990	2	92	29	2		CN42449_S62
42451	Russia	1984	2010	3	88				CN42451_S63
46339	Canada	1991	2010	3	94			94	CN46339_S64
52040	China	1993	2010	1	96		1	94	CN52040_S65
52051	China	1993	2010	1	92		1		CN52051_S66
52061	China	1993	2010	1	90		1		CN52061_S67
33815	Canada	1971	1987	2	58	29	2	70	CN33815i102_S68
36285	Canada	1980	1990	1	44	29	1	70	CN36285i101_S69
36882	USA	1980	1990	4	46	29		70	CN36882i104_S70
36990	USA	1980	1985	1	56	29	1	70	CN36990i101_S71
36295_R2	Canada	1980	2012	1	40		1		CN36295i101x2_S72

Table S2f. List of inventory and RNA-Seq information for 72 assayed rapa samples representing their respective PGRC collection, along with the composition of three paired groups for group analysis. CN=PGRC accession number, Country=country of origin, YA=year acquired, YR=year last regenerated, NR=the number of field regenerations done since the acquisition, GR=germination rate, SY=grouping based on the year last regenerated, RF=grouping based on NR, GD=grouping based on GR, and SEQ-Name=sequence file name.

Rapa						Group composition			Sample
CN	Country	YA	YR	NR	GR	SY	RF	GD	SEQ-Name
101878	Pakistan	2000	2016	2	95	5	2		CN101878_S1
101886	Pakistan	2000	2000	1	98	16	1	96	CN101886_S2
101888	Pakistan	2000	2000	1	93	16			CN101888_S3
101892	Pakistan	2000	2000	1	90	16			CN101892_S4
101908	Pakistan	2000	2016	2	94	5	2		CN101908_S5
101915	Pakistan	2000	2000	1	97	16	1	96	CN101915_S6
101917	Pakistan	2000	2000	1	77	16			CN101917_S7
101941	Pakistan	2000	2000	1	88	16			CN101941_S8
105404	China	1993	2015	1	97	5	1	96	CN105404_S9
105412	China	1993	2001	1	59	16		69	CN105412_S10
107713	Germany	1983	2003	1	86	16			CN107713_S11
107744	India	1969	2003	1	92	16	1		CN107744_S12
107748	India	1969	2015	1	95	5			CN107748_S13
107765	Finland	1993	2015	1	87	5			CN107765_S14
107778	China	1993	2003	1	55	16	1	69	CN107778_S15
110921	Nepal	1976	2016	2	91	5	2		CN110921_S16
110930	Nepal	1976	2004	1	80				CN110930_S17
110934	Nepal	1976	2003	1	88	16			CN110934_S18
110946	Nepal	1976	2016	2	96	5	2	96	CN110946_S19
110956	Unknown	1976	2005	1	93		1		CN110956_S20
110957	USA	1976	2016	2	96	5	2	96	CN110957_S21
110958	Nepal	1976	2004	1	83				CN110958_S22
110962	Nepal	1976	2005	1	87				CN110962_S23
110964	Nepal	1976	2016	2	92	5	2		CN110964_S24
110971	Nepal	1976	2003	1	93	16	1		CN110971_S25
110972	Nepal	1976	2003	1	92	16			CN110972_S26
110974	Canada	1976	2003	1	84	16			CN110974_S27
110999	Pakistan	1980	2003	1	85	16			CN110999_S28
111012	Germany	1983	2003	1	78	16			CN111012_S29
111017	India	1990	2005	1	71				CN111017_S30
111023	Pakistan	1985	2003	1	93	16			CN111023_S31
111036	Pakistan	1985	2016	2	96	5	2	96	CN111036_S32
111038	Pakistan	1985	2005	1	92		1		CN111038_S33
111042	Pakistan	1985	2004	1	93				CN111042_S34
111046	Pakistan	1985	2004	1	97		1	96	CN111046_S35
111048	Pakistan	1985	2004	1	98			96	CN111048_S36
111059	Pakistan	1985	2004	1	82				CN111059_S37
111084	Pakistan	1985	2016	2	99	5	2	96	CN111084_S38
111097	India	1985	2004	1	82				CN111097_S39
111107	India	1985	2004	1	62			69	CN111107_S40
111111	India	1985	2004	1	89		1		CN111111_S41
111116	India	1985	2016	2	100	5	2	96	CN111116_S42
111139	Turkey	1985	2004	1	83				CN111139_S43
111157	Afghanistan	1985	2016	2	84	5	2		CN111157_S44
111173	India	1985	2004	1	71				CN111173_S45
111177	India	1985	2016	2	80	5	2		CN111177_S46
111187	India	1985	2016	2	87	5	2		CN111187_S47
107778_R1	China	1993	2003	1	55		1		CN107778x1_S48
111190	India	1985	2004	1	84				CN111190_S49
111195	India	1985	2004	1	72		1		CN111195_S50
111228	India	1985	2004	1	74				CN111228_S51
111229	India	1985	2004	1	82				CN111229_S52
111232	India	1985	2016	2	77	5	2		CN111232_S53
111242	Afghanistan	1985	2004	1	90				CN111242_S54
111267	Unknown	1985	2004	1	52			69	CN111267_S55
111272	Unknown	1985	2004	1	92				CN111272_S56
111293	Unknown	1985	2004	1	32			69	CN111293_S57
111324	Canada	2002	2004	1	8			69	CN111324_S58
112919	Unknown	1987	2005	1	68			69	CN112919_S59
112921	Canada	1994	2005	1	69			69	CN112921_S60
112924	Canada	1994	2005	1	79				CN112924_S61
113058	Unknown	1985	2006	1	83		1		CN113058_S62
115107	China	1993	2005	1	54			69	CN115107_S63
115245	Russia	2008	2010	1	88		1		CN115245_S64
19060	Canada	2001	2015	2	73	5	2		CN19060_S65
35815	Russia	1979	1979	0	1	16		69	CN35815_S66
35956	Japan	1979	2016	3	98	5		96	CN35956_S67
36094	China	1979	2016	2	98	5	2	96	CN36094_S68
44103	Pakistan	1986	2014	1	95	5			CN44103_S69
31402	Canada	1971	1978	1	2	16		69	CN31402_S70
44108	China	1986	1986	0	4	16		69	CN44108_S71
107778_R2	China	1993	2003	1	55		1		CN107778x2_S72

Table S2g. List of inventory and RNA-Seq information for 72 assayed sunflower samples representing their respective PGRC collection, along with the composition of three paired groups for group analysis. CN=PGRC accession number, Country=country of origin, YA=year acquired, YR=year last regenerated, NR=the number of field regenerations done since the acquisition, GR=germination rate, SY=grouping based on the year last regenerated, RF=grouping based on NR, GD=grouping based on GR, and SEQ-Name=sequence file name.

Sunflower						Group composition			Sample
CN	Country	YA	YR	NR	GR	SY	RF	GD	SEQ-Name
29474	Russia	1973	2005	1	78		1		CN29474_S1
29665	China	1974	2007	1	72		1		CN29665_S2
31851	Germany	1977	2007	2	80		2		CN31851_S3
32248	France	1978	2007	2	58		2		CN32248_S4
32252	France	1978	2007	5	60				CN32252_S5
32627	Russia	1978	2007	4	88			86	CN32627_S6
33282	Canada	1971	2007	4	32			50	CN33282_S7
33285	Canada	1975	2007	1	50		1	50	CN33285_S8
33963	Russia	1978	1992	1	70	27	1		CN33963_S9
35597	USA	1979	2007	3	53				CN35597_S10
36523	Canada	1980	2007	2	70		2		CN36523_S11
36542	Canada	1980	2007	2	62		2		CN36542_S12
36543	Canada	1980	2007	2	50			50	CN36543_S13
36554	Canada	1980	2007	2	92		2	86	CN36554_S14
36555	Canada	1980	2007	1	83		1		CN36555_S15
36564	Canada	1980	2007	2	56		2		CN36564_S16
36566	Canada	1980	2007	2	80		2		CN36566_S17
36573	Canada	1980	2007	2	94		2	86	CN36573_S18
36574	Canada	1980	2007	2	90		2	86	CN36574_S19
36575	Canada	1980	2007	2	63		2		CN36575_S20
36582	Canada	1980	2007	2	77				CN36582_S21
36602	Canada	1980	2007	2	80		2		CN36602_S22
36603	Canada	1980	2007	2	42		2	50	CN36603_S23
36609	Canada	1980	2007	2	75		2		CN36609_S24
36613	Canada	1980	2007	2	90		2	86	CN36613_S25
36615	Canada	1980	2007	2	76				CN36615_S26
36622	Canada	1980	2007	2	76		2		CN36622_S27
36641	Canada	1980	2007	2	91		2	86	CN36641_S28
36651	Canada	1980	2007	2	93		2	86	CN36651_S29
36652	Canada	1980	2007	2	75				CN36652_S30
36676	Canada	1980	2007	2	78		2		CN36676_S31
36677	Canada	1980	2007	2	32		2	50	CN36677_S32
36679	Canada	1980	2013	3	68	6			CN36679_S33
36686	Canada	1980	2007	2	77		2		CN36686_S34
36689	Canada	1980	2007	2	49			50	CN36689_S35
36701	Canada	1980	2007	2	78		2		CN36701_S36
36703	Canada	1980	1989	1	89	27	1	86	CN36703_S37
36707	Canada	1980	1989	1	48	27	1	50	CN36707_S38
36711	Canada	1980	1989	1	44	27	1	50	CN36711_S39
36719	Canada	1980	2005	3	87			86	CN36719_S40
39651	Canada	1982	2005	2	20		2	50	CN39651_S41
39656	Canada	1982	2005	1	29		1	50	CN39656_S42
39658	Canada	1982	2014	1	70	6	1		CN39658_S43
39666	Serbia	1982	2014	2	81	6	2		CN39666_S44
40468	France	1983	2006	4	94			86	CN40468_S45
40469	France	1983	2013	5	93			86	CN40469_S46
42228	Canada	1983	2005	2	53		2		CN42228_S47
33285_R1	Canada	1975	2007	1	50		1		CN33285x1_S48
42238	Canada	1983	1983	0	21	27		50	CN42238_S49
42239	Canada	1983	2014	2	74	6			CN42239_S50
42248	France	1983	1983	0	26	27		50	CN42248_S51
42249	France	1983	1989	1	52	27	1		CN42249_S52
42253	Canada	1983	1989	1	74	27	1		CN42253_S53
42259	Canada	1983	1983	0	25	27		50	CN42259_S54
42273	Romania	1983	1989	1	73	27	1		CN42273_S55
42279	Canada	1983	1989	1	63	27	1		CN42279_S56
42280	South Africa	1983	1989	1	84	27	1		CN42280_S57
42282	Canada	1983	1989	1	81	27	1		CN42282_S58
42284	Canada	1983	1983	0	62	27			CN42284_S59
42285	Canada	1983	1989	1	75	27	1		CN42285_S60
42307	USA	1983	2014	1	90	6	1	86	CN42307_S61
42333	Canada	1983	2015	2	81	6	2		CN42333_S62
42335	USA	1983	2015	1	88	6	1	86	CN42335_S63
42342	USA	1983	2015	1	61	6	1		CN42342_S64
42754	Canada	1984	2015	1	59	6	1		CN42754_S65
42765	Canada	1984	2015	1	48	6	1	50	CN42765_S66
51712	Canada	1989	2015	2	86	6	2	86	CN51712_S67
51717	Canada	1989	2015	2	72	6	2		CN51717_S68
51816	Canada	1992	2015	1	78	6	1		CN51816_S69
51817	Canada	1992	2015	1	73	6	1		CN51817_S70
52559	Canada	1994	2015	1	67	6	1		CN52559_S71
33285_R2	Canada	1975	2007	1	50		1		CN33285x2_S72

Table S3a. Summary of RNA-Seq sequence reads before trimming, after trimming, and after alignment for the samples of the barley and wheat collections.

Sample	Original fastq paired reads	Trimmed fastq paired reads	Percent trimmed paired reads	Total reads in bam file	Total mapped reads in bam file	Percent of mapped reads in bam file	Sample	Original fastq paired reads	Trimmed fastq paired reads	Percent trimmed paired reads	Total reads in bam file	Total mapped reads in bam file	Percent of mapped reads in bam file
**Barley**							**Wheat**						
CN081789_S1	10,660,962	9,383,309	88.02	15,199,886	12,138,625	79.86	CN001686_S1	8,569,644	7,525,152	87.81	15,027,153	11,454,817	76.23
CN058854_S2	9,115,662	7,952,425	87.24	13,262,062	10,669,283	80.45	CN001693_S2	6,310,804	5,619,426	89.04	11,504,887	8,788,245	76.39
CN074861_S3	7,096,587	6,175,925	87.03	10,773,492	8,650,867	80.30	CN001694_S3	9,121,387	8,120,680	89.03	15,848,974	11,867,899	74.88
CN074403_S4	8,835,771	7,654,319	86.63	12,933,462	10,326,576	79.84	CN002657_S4	8,041,065	7,086,681	88.13	13,689,431	10,459,722	76.41
CN075883_S5	10,468,457	9,255,249	88.41	15,121,692	12,208,743	80.74	CN002698_S5	9,942,252	8,891,429	89.43	17,066,812	12,798,046	74.99
CN069054_S6	8,688,664	7,595,676	87.42	12,358,141	9,955,820	80.56	CN002707_S6	9,801,836	8,613,156	87.87	16,987,742	12,585,262	74.08
CN069156_S7	10,430,993	9,135,773	87.58	13,340,708	10,812,110	81.05	CN005231_S7	10,255,438	8,995,691	87.72	17,573,510	13,470,955	76.65
CN043596_S8	9,510,238	8,288,499	87.15	13,393,066	10,674,519	79.70	CN005262_S8	7,539,519	6,561,534	87.03	13,260,335	10,179,848	76.77
CN083382_S9	8,608,209	7,431,124	86.33	12,624,358	10,216,896	80.93	CN005815_S9	10,881,719	9,564,898	87.90	18,732,576	13,927,595	74.35
CN074798_S10	10,044,246	8,849,109	88.10	15,027,281	12,033,124	80.08	CN005823_S10	10,195,864	9,052,103	88.78	17,801,229	13,209,323	74.20
CN033367_S11	8,135,277	7,268,768	89.35	11,639,252	9,368,175	80.49	CN005945_S11	9,465,426	8,407,913	88.83	16,354,116	12,405,272	75.85
CN088403_S12	9,256,802	8,254,641	89.17	13,725,620	11,075,873	80.69	CN006124_S12	12,986,147	11,542,415	88.88	21,888,100	16,427,272	75.05
CN059701_S13	7,400,118	6,483,532	87.61	11,014,606	8,866,837	80.50	CN006869_S13	14,895,972	13,041,753	87.55	23,937,624	18,001,482	75.20
CN031049_S14	7,868,205	6,979,374	88.70	11,770,030	9,435,314	80.16	CN007189_S14	12,442,168	11,043,660	88.76	20,879,858	15,772,235	75.54
CN000231_S15	8,909,029	7,781,712	87.35	12,379,593	9,971,392	80.55	CN007228_S15	11,322,366	9,900,576	87.44	18,813,226	14,360,832	76.33
CN000172_S16	10,059,825	8,802,170	87.50	13,584,191	10,899,658	80.24	CN009783_S16	9,901,547	8,800,080	88.88	17,350,376	13,266,793	76.46
CN012988_S17	11,644,044	10,110,082	86.83	15,006,731	12,134,838	80.86	CN009821_S17	9,066,446	7,990,860	88.14	15,924,683	12,085,114	75.89
CN000290_S18	10,215,448	8,933,839	87.45	13,628,718	10,978,438	80.55	CN010157_S18	8,868,397	7,781,674	87.75	15,337,603	11,609,131	75.69
CN039732_S19	11,147,129	9,767,607	87.62	15,227,593	12,217,498	80.23	CN010960_S19	7,516,013	6,602,745	87.85	13,109,383	9,900,364	75.52
CN089110_S20	10,613,652	9,097,869	85.72	13,496,192	10,941,942	81.07	CN011045_S20	10,332,179	9,116,177	88.23	17,999,036	13,496,838	74.99
CN089156_S21	10,132,498	8,833,467	87.18	14,644,538	11,727,578	80.08	CN011061_S21	11,427,285	10,110,547	88.48	19,380,416	14,424,829	74.43
CN000252_S22	11,078,677	9,668,967	87.28	15,264,683	12,255,311	80.29	CN011150_S22	12,529,532	10,958,551	87.46	21,159,927	15,943,842	75.35
CN090066_S23	10,535,508	9,176,809	87.10	14,768,704	11,900,989	80.58	CN011189_S23	10,592,882	9,406,810	88.80	18,484,825	14,035,612	75.93
CN058689_S24	10,184,314	8,843,549	86.83	14,390,737	11,568,001	80.39	CN011993_S24	8,901,010	7,830,693	87.98	14,933,742	11,359,835	76.07
CN058963_S25	8,606,897	7,652,032	88.91	12,552,227	10,119,998	80.62	CN011995_S25	14,365,038	12,456,773	86.72	24,067,591	17,540,339	72.88
CN059461_S26	7,052,009	6,245,057	88.56	9,834,428	7,945,850	80.80	CN012124_S26	11,213,687	9,795,475	87.35	18,062,555	13,518,271	74.84
CN059577_S27	6,319,803	5,630,772	89.10	9,810,107	7,892,431	80.45	CN012349_S27	9,284,831	8,066,484	86.88	15,814,705	11,872,637	75.07
CN060340_S28	9,424,817	8,382,828	88.94	11,930,800	9,706,669	81.36	CN029758_S28	12,058,626	10,447,379	86.64	20,331,049	15,344,378	75.47
CN083473_S29	8,505,690	7,540,331	88.65	12,140,491	9,797,137	80.70	CN029767_S29	11,837,836	10,376,546	87.66	19,795,822	15,021,021	75.88
CN000296_S30	8,686,116	7,704,639	88.70	12,470,889	9,975,021	79.99	CN030240_S30	12,021,759	10,566,403	87.89	20,048,351	15,112,477	75.38
CN078926_S31	9,835,187	8,807,157	89.55	12,768,241	10,340,577	80.99	CN030279_S31	10,702,333	9,364,933	87.50	18,063,034	13,427,233	74.34
CN080762_S32	9,135,646	8,194,555	89.70	13,165,117	10,608,856	80.58	CN030294_S32	12,425,607	10,822,609	87.10	20,294,521	15,351,208	75.64
CN089775_S33	6,842,774	6,128,019	89.55	10,679,864	8,518,276	79.76	CN030302_S33	10,298,583	9,004,663	87.44	17,809,611	13,424,978	75.38
CN000279_S34	9,668,579	8,624,792	89.20	14,092,775	11,397,945	80.88	CN030303_S34	10,434,384	9,062,044	86.85	17,243,270	13,065,240	75.77
CN077017_S35	9,453,440	8,428,016	89.15	13,347,693	10,737,863	80.45	CN030614_S35	8,458,473	7,375,695	87.20	14,122,208	10,789,190	76.40
CN000199_S36	8,274,533	7,328,819	88.57	11,376,986	9,188,002	80.76	CN032480_S36	10,754,449	9,320,201	86.66	17,592,010	13,434,687	76.37
CN038783_S37	5,969,355	4,413,730	73.94	6,038,460	4,985,744	82.57	CN032569_S37	10,400,869	9,011,052	86.64	17,372,912	13,352,875	76.86
CN089912_S38	7,833,050	6,959,585	88.85	11,715,934	9,431,565	80.50	CN039669_S38	8,365,496	7,213,704	86.23	13,910,915	10,646,409	76.53
CN003843_S39	9,287,466	8,256,672	88.90	13,097,627	10,558,875	80.62	CN040652_S39	10,147,009	8,731,625	86.05	16,978,760	13,213,451	77.82
CN058516_S40	7,208,372	6,362,607	88.27	10,992,876	8,860,712	80.60	CN040693_S40	11,077,327	9,645,087	87.07	18,007,540	13,746,145	76.34
CN060784_S41	6,442,009	5,754,346	89.33	9,575,121	7,804,834	81.51	CN040719_S41	8,155,303	7,117,214	87.27	14,071,599	10,730,427	76.26
CN044439_S42	10,965,070	9,863,115	89.95	14,485,158	11,801,860	81.48	CN040736_S42	8,940,943	7,841,852	87.71	15,672,180	11,775,419	75.14
CN004072_S43	7,187,744	6,433,461	89.51	10,446,029	8,570,573	82.05	CN040791_S43	11,900,097	10,439,516	87.73	19,043,289	14,474,303	76.01
CN039283_S44	8,918,878	8,092,349	90.73	12,453,299	10,187,133	81.80	CN040828_S44	9,143,337	8,045,078	87.99	15,434,272	11,818,063	76.57
CN083925_S45	8,337,877	7,490,342	89.84	11,324,763	9,225,480	81.46	CN040829_S45	8,437,748	7,338,916	86.98	14,072,833	10,594,104	75.28
CN029180_S46	7,813,145	7,029,364	89.97	11,397,634	9,385,907	82.35	CN040830_S46	6,658,662	5,793,820	87.01	11,397,367	8,615,128	75.59
CN000189_S47	9,593,052	8,062,708	84.05	11,166,238	9,275,159	83.06	CN040831_S47	9,658,665	8,334,303	86.29	15,644,388	11,980,444	76.58
CN039732X1_S48	8,975,489	7,726,234	86.08	11,765,477	9,590,326	81.51	CN001694X1_S48	11,047,437	9,698,438	87.79	18,560,328	13,848,093	74.61
CN000012_S49	9,099,093	8,071,690	88.71	12,918,668	10,508,875	81.35	CN040855_S49	9,676,978	8,310,423	85.88	15,531,241	11,988,719	77.19
CN043386_S50	8,337,062	7,357,027	88.24	11,736,076	9,601,008	81.81	CN040857_S50	10,050,861	8,625,896	85.82	15,985,738	12,243,698	76.59
CN080492_S51	9,042,085	7,795,303	86.21	12,301,037	10,078,032	81.93	CN040864_S51	10,439,845	8,977,702	85.99	16,758,609	12,657,520	75.53
CN068434_S52	12,422,107	10,314,158	83.03	13,531,006	10,983,464	81.17	CN040865_S52	11,317,318	9,782,027	86.43	18,186,373	13,608,447	74.83
CN074807_S53	7,972,593	6,868,189	86.15	12,199,990	9,684,367	79.38	CN040868_S53	9,012,717	7,758,697	86.09	14,615,113	11,123,328	76.11
CN058544_S54	7,549,423	6,678,019	88.46	11,260,727	9,028,807	80.18	CN040874_S54	10,610,837	9,135,524	86.10	16,866,634	12,921,787	76.61
CN035142_S55	7,996,235	6,944,278	86.84	11,777,660	9,453,197	80.26	CN040906_S55	9,380,897	8,008,570	85.37	14,912,067	11,263,357	75.53
CN081927_S56	9,957,941	8,758,909	87.96	14,476,323	11,594,421	80.09	CN042510_S56	8,449,329	7,029,633	83.20	13,843,616	10,646,079	76.90
CN082033_S57	12,424,010	10,858,054	87.40	16,044,592	12,932,512	80.60	CN042511_S57	11,666,213	9,893,940	84.81	17,862,254	13,616,906	76.23
CN060487_S58	11,127,736	9,829,966	88.34	15,078,492	12,157,427	80.63	CN042519_S58	12,363,879	10,411,600	84.21	19,405,958	14,554,165	75.00
CN039017_S59	7,561,966	6,755,159	89.33	10,576,699	8,555,286	80.89	CN042528_S59	13,107,403	11,130,931	84.92	20,560,842	15,927,783	77.47
CN080080_S60	11,296,306	9,426,671	83.45	12,228,707	10,022,577	81.96	CN043843_S60	12,681,806	10,766,851	84.90	20,610,950	15,644,680	75.90
CN045934_S61	7,555,688	6,741,415	89.22	11,025,654	8,786,764	79.69	CN043980_S61	10,646,031	9,095,653	85.44	17,461,828	13,001,063	74.45
CN069320_S62	10,510,705	9,259,384	88.09	13,495,115	10,916,281	80.89	CN044047_S62	12,714,249	10,776,694	84.76	20,486,242	15,519,981	75.76
CN039337_S63	7,991,128	7,068,494	88.45	12,015,742	9,668,129	80.46	CN044051_S63	10,520,193	9,056,552	86.09	17,626,451	13,137,839	74.53
CN038780_S64	9,334,275	8,052,379	86.27	12,840,075	10,340,753	80.53	CN044392_S64	14,850,252	12,683,278	85.41	22,643,968	16,966,072	74.93
CN038782_S65	9,100,340	8,016,490	88.09	13,143,773	10,556,495	80.32	CN044409_S65	9,482,591	8,128,965	85.73	15,721,273	12,180,653	77.48
CN000002_S66	10,280,470	9,058,669	88.12	15,070,578	12,164,168	80.71	CN044427_S66	10,744,327	9,192,614	85.56	17,754,021	13,637,100	76.81
CN088430_S67	8,167,379	7,257,461	88.86	11,772,297	9,538,903	81.03	CN045577_S67	8,954,837	7,634,776	85.26	15,182,705	11,368,707	74.88
CN089697_S68	8,219,011	7,363,730	89.59	11,626,934	9,430,258	81.11	CN045594_S68	9,414,739	8,083,759	85.86	15,800,267	11,942,939	75.59
CN089814_S69	11,349,320	10,137,263	89.32	15,628,788	12,635,449	80.85	CN045604_S69	9,630,231	8,193,287	85.08	15,875,513	12,111,630	76.29
CN090068_S70	10,643,700	9,518,807	89.43	15,211,826	12,278,164	80.71	CN051260_S70	11,077,479	9,512,475	85.87	18,383,201	14,054,938	76.46
CN090767_S71	8,957,947	7,903,157	88.23	12,902,183	10,432,174	80.86	CN106318_S71	12,239,544	10,511,045	85.88	19,590,721	14,918,011	76.15
CN039732X2_S72	11,152,139	9,877,692	88.57	16,107,441	12,896,697	80.07	CN001694X2_S72	11,617,789	9,951,964	85.66	18,914,004	14,179,261	74.97

Table S3b. Summary of RNA-Seq sequence reads before trimming, after trimming, and after alignment for the samples of the oat and soybean and collections.

Sample	Original fastq paired reads	Trimmed fastq paired reads	Percent trimmed paired reads	Total reads in bam file	Total mapped reads in bam file	Percent of mapped reads in bam file
**Oat**						
CN003161_S1	7,817,536	6,971,127	89.17	13,634,588	10,978,439	80.52
CN004395_S2	9,002,081	8,062,992	89.57	15,017,350	12,195,201	81.21
CN004873_S3	8,213,543	7,350,916	89.50	14,403,748	11,664,676	80.98
CN005187_S4	9,541,894	8,553,607	89.64	15,554,484	12,466,567	80.15
CN021346_S5	8,228,777	7,285,381	88.54	14,015,072	11,304,647	80.66
CN021953_S6	10,282,477	9,026,537	87.79	16,314,900	13,242,839	81.17
CN025369_S7	8,678,021	7,540,464	86.89	14,311,913	11,533,591	80.59
CN043863_S8	9,785,075	7,951,195	81.26	14,513,992	11,767,598	81.08
CN051320_S9	6,848,984	6,108,065	89.18	12,317,796	9,907,990	80.44
CN053565_S10	7,316,668	6,552,490	89.56	13,063,490	10,593,639	81.09
CN053617_S11	9,011,884	7,982,536	88.58	15,361,136	12,393,155	80.68
CN053840_S12	8,802,642	7,911,209	89.87	15,002,747	12,057,341	80.37
CN054228_S13	6,881,842	6,077,972	88.32	12,172,581	9,799,432	80.50
CN054851_S14	9,021,476	8,161,636	90.47	15,848,054	12,705,284	80.17
CN063442_S15	8,402,376	7,554,963	89.91	15,092,313	12,046,377	79.82
CN063751_S16	10,271,988	9,343,840	90.96	17,759,319	14,185,693	79.88
CN063914_S17	8,246,257	7,394,424	89.67	14,626,771	11,806,094	80.72
CN063916_S18	11,188,920	10,035,237	89.69	18,714,903	15,069,550	80.52
CN063969_S19	7,089,233	6,369,649	89.85	12,793,692	10,302,529	80.53
CN064317_S20	6,440,057	5,775,023	89.67	11,793,726	9,448,347	80.11
CN064431_S21	7,348,363	6,546,128	89.08	13,210,969	10,616,717	80.36
CN064654_S22	7,455,316	6,697,334	89.83	13,391,700	10,779,958	80.50
CN064947_S23	8,922,805	7,986,686	89.51	14,587,358	11,866,574	81.35
CN065015_S24	9,207,499	8,191,650	88.97	15,517,879	12,570,012	81.00
CN065073_S25	10,951,037	9,644,002	88.06	17,850,169	14,567,981	81.61
CN065157_S26	10,099,776	8,913,656	88.26	16,850,580	13,702,528	81.32
CN067324_S27	8,850,521	7,857,342	88.78	15,153,625	12,234,808	80.74
CN072565_S28	8,111,763	7,164,770	88.33	14,152,254	11,487,714	81.17
CN074849_S29	12,435,034	11,000,461	88.46	20,548,969	16,628,454	80.92
CN079297_S30	12,187,626	10,784,630	88.49	20,671,522	16,684,904	80.71
CN079327_S31	8,900,745	7,881,840	88.55	15,695,954	12,580,392	80.15
CN079332_S32	12,411,571	10,907,852	87.88	20,187,416	16,328,659	80.89
CN080895_S33	7,829,510	6,867,520	87.71	13,781,851	11,212,995	81.36
CN081704_S34	9,280,525	8,156,247	87.89	16,054,861	12,885,748	80.26
CN081756_S35	6,960,365	6,136,513	88.16	12,631,278	10,158,471	80.42
CN088377_S36	8,908,337	7,814,109	87.72	15,767,052	12,639,848	80.17
CN088392_S37	11,554,111	10,159,266	87.93	19,358,219	15,493,856	80.04
CN088434_S38	12,526,737	10,874,495	86.81	19,770,381	15,990,985	80.88
CN088457_S39	9,596,696	8,480,270	88.37	16,840,090	13,518,763	80.28
CN088459_S40	11,160,640	9,856,765	88.32	19,280,226	15,377,376	79.76
CN088611_S41	10,969,682	9,430,412	85.97	17,450,111	14,109,484	80.86
CN088662_S42	8,738,597	7,546,287	86.36	14,083,161	11,291,514	80.18
CN088678_S43	9,405,392	8,236,997	87.58	16,084,177	12,794,623	79.55
CN088707_S44	9,308,595	8,208,642	88.18	15,976,401	12,813,160	80.20
CN088739_S45	8,235,867	7,309,540	88.75	14,687,420	11,814,745	80.44
CN088747_S46	12,760,365	11,306,758	88.61	21,996,667	17,597,535	80.00
CN088757_S47	8,210,798	7,204,902	87.75	14,826,003	11,873,945	80.09
CN063442X1_S48	8,824,545	7,496,093	84.95	14,928,666	12,043,672	80.67
CN088758_S49	9,187,424	7,487,369	81.50	15,265,665	12,234,675	80.15
CN088767_S50	8,523,363	7,346,360	86.19	14,809,935	11,860,159	80.08
CN088828_S51	9,764,079	8,310,703	85.12	16,147,630	12,923,700	80.03
CN088832_S52	9,626,858	8,175,067	84.92	15,706,892	12,677,587	80.71
CN088842_S53	9,450,168	8,155,609	86.30	16,041,929	12,836,658	80.02
CN088844_S54	7,040,064	5,932,780	84.27	12,222,299	9,735,643	79.65
CN088854_S55	8,766,262	7,464,511	85.15	14,762,815	11,778,183	79.78
CN088917_S56	9,312,976	7,938,471	85.24	15,493,645	12,500,989	80.68
CN088968_S57	13,336,239	11,077,189	83.06	20,640,872	16,545,934	80.16
CN090177_S58	11,011,257	9,223,478	83.76	17,729,494	14,287,571	80.59
CN090178_S59	8,827,606	7,627,044	86.40	14,919,314	12,042,600	80.72
CN090182_S60	13,156,713	11,501,330	87.42	21,234,551	17,055,259	80.32
CN090190_S61	9,975,631	8,609,355	86.30	16,980,958	13,523,187	79.64
CN090205_S62	12,141,037	10,421,890	85.84	18,685,445	15,066,699	80.63
CN090299_S63	12,299,047	10,544,998	85.74	20,241,344	16,090,783	79.49
CN090336_S64	13,031,420	11,192,200	85.89	21,143,208	16,865,502	79.77
CN090364_S65	13,824,435	11,053,178	79.95	20,944,563	16,721,356	79.84
CN090395_S66	11,722,077	10,164,619	86.71	20,067,465	15,967,979	79.57
CN090447_S67	13,764,905	11,870,942	86.24	22,513,942	17,984,257	79.88
CN090469_S68	12,336,184	10,683,111	86.60	20,843,367	16,671,488	79.98
CN090470_S69	12,403,164	10,833,487	87.34	20,529,756	16,413,379	79.95
CN090477_S70	11,921,884	10,350,215	86.82	20,381,048	16,295,739	79.96
CN099001_S71	8,165,575	7,061,591	86.48	14,003,651	11,250,580	80.34
CN063442X2_S72	11,877,964	10,194,508	85.83	19,496,264	15,725,999	80.66

Sample	Original fastq paired reads	Trimmed fastq paired reads	Percent trimmed paired reads	Total reads in bam file	Total mapped reads in bam file	Percent of mapped reads in bam file
**Soybean**						
CN029747_S1	9,578,353	8,323,230	86.90	13,668,541	9,570,748	70.02
CN029793_S2	8,249,317	7,214,745	87.46	11,726,269	8,203,575	69.96
CN030316_S3	7,116,266	6,189,917	86.98	10,295,379	7,331,417	71.21
CN030318_S4	8,883,623	7,689,462	86.56	13,383,485	9,419,766	70.38
CN031719_S5	9,626,195	8,329,107	86.53	13,615,275	10,070,755	73.97
CN031984_S6	10,986,491	9,528,589	86.73	15,805,717	11,207,424	70.91
CN032354_S7	8,988,981	7,845,935	87.28	12,781,456	9,436,092	73.83
CN032634_S8	10,482,630	9,083,677	86.65	15,150,409	10,876,684	71.79
CN032766_S9	12,007,400	10,422,454	86.80	15,981,848	11,303,307	70.73
CN033248_S10	12,550,901	10,230,078	81.51	16,433,300	11,689,426	71.13
CN033253_S11	11,828,191	10,341,755	87.43	17,482,194	11,500,464	65.78
CN033255_S12	10,300,792	8,919,550	86.59	14,896,787	10,470,278	70.29
CN033257_S13	13,498,946	11,801,265	87.42	18,864,610	13,212,185	70.04
CN033259_S14	16,526,877	12,916,445	78.15	17,747,177	12,727,942	71.72
CN033267_S15	12,111,774	10,040,181	82.90	14,693,474	10,741,333	73.10
CN033279_S16	14,148,025	12,296,398	86.91	18,993,545	13,343,858	70.25
CN033908_S17	11,041,498	9,443,816	85.53	12,090,669	8,518,340	70.45
CN035756_S18	9,195,745	8,045,741	87.49	12,464,164	9,107,337	73.07
CN035920_S19	10,483,042	9,135,677	87.15	13,608,213	9,496,350	69.78
CN036009_S20	11,317,033	9,657,482	85.34	14,623,028	10,568,441	72.27
CN036215_S21	11,360,678	9,986,487	87.90	15,347,229	10,657,674	69.44
CN036333_S22	9,393,404	8,146,426	86.72	12,991,112	9,193,736	70.77
CN036337_S23	9,122,878	7,892,029	86.51	11,129,526	8,340,860	74.94
CN036342_S24	11,610,983	9,965,326	85.83	13,695,178	9,800,406	71.56
CN039077_S25	9,880,220	8,336,715	84.38	13,261,801	9,691,018	73.07
CN039150_S26	7,048,706	5,915,960	83.93	9,426,717	6,656,959	70.62
CN039153_S27	11,013,340	9,551,029	86.72	14,787,646	10,082,514	68.18
CN039178_S28	12,742,126	11,105,435	87.16	17,426,365	12,144,417	69.69
CN039193_S29	10,320,818	8,985,446	87.06	14,555,734	9,802,405	67.34
CN042220_S30	9,589,015	8,272,579	86.27	14,076,927	9,727,025	69.10
CN042387_S31	10,412,326	9,054,870	86.96	15,547,261	10,939,917	70.37
CN042388_S32	10,673,784	9,281,547	86.96	15,453,799	10,720,341	69.37
CN042536_S33	11,643,060	10,123,376	86.95	15,489,201	11,196,114	72.28
CN042928_S34	9,654,519	8,307,380	86.05	12,112,521	8,342,875	68.88
CN045087_S35	11,577,350	10,161,034	87.77	16,437,118	11,622,380	70.71
CN045092_S36	10,705,676	9,332,574	87.17	15,532,396	10,829,710	69.72
CN045107_S37	11,424,450	9,944,498	87.05	16,709,260	11,376,522	68.09
CN051375_S38	14,553,647	12,753,813	87.63	19,928,519	13,697,627	68.73
CN051844_S39	11,285,265	9,823,659	87.05	15,979,052	10,745,736	67.25
CN051845_S40	10,872,059	9,612,674	88.42	15,016,413	10,732,510	71.47
CN052635_S41	9,092,210	7,912,520	87.03	13,664,243	9,311,328	68.14
CN052644_S42	9,912,251	8,567,745	86.44	14,550,971	10,452,960	71.84
CN052648_S43	9,760,424	8,471,786	86.80	14,313,484	10,220,543	71.40
CN052653_S44	10,895,812	9,466,268	86.88	15,614,885	10,816,777	69.27
CN052740_S45	10,707,915	9,328,843	87.12	15,380,569	10,856,274	70.58
CN052873_S46	10,238,396	8,888,875	86.82	14,684,724	10,603,880	72.21
CN107352_S47	10,885,043	9,526,351	87.52	15,264,574	10,594,809	69.41
CN032634X1_S48	9,533,203	8,286,996	86.93	14,049,568	9,975,649	71.00
CN107360_S49	10,422,112	9,032,122	86.66	14,394,569	9,778,248	67.93
CN107370_S50	9,573,933	8,334,407	87.05	13,607,102	9,451,254	69.46
CN107373_S51	10,768,277	9,377,429	87.08	15,991,393	7,500,387	46.90
CN107385_S52	9,525,401	8,211,305	86.20	13,575,440	9,836,527	72.46
CN107406_S53	10,725,820	9,346,705	87.14	15,787,522	11,183,914	70.84
CN107407_S54	8,948,363	7,692,401	85.96	13,209,201	9,432,067	71.41
CN107420_S55	10,282,066	8,961,021	87.15	14,674,170	10,379,896	70.74
CN107421_S56	10,431,819	9,004,415	86.32	15,319,408	10,959,073	71.54
CN107422_S57	12,263,354	10,701,610	87.26	17,443,255	12,083,800	69.27
CN107436_S58	13,680,235	10,007,759	73.15	12,266,283	8,756,146	71.38
CN107449_S59	10,560,335	9,307,010	88.13	15,797,373	10,880,696	68.88
CN107450_S60	10,202,160	8,898,160	87.22	15,012,514	10,482,451	69.82
CN107459_S61	13,611,671	11,549,538	84.85	16,092,139	11,353,077	70.55
CN107472_S62	14,687,513	12,841,944	87.43	19,217,163	13,125,269	68.30
CN107515_S63	10,906,314	9,525,785	87.34	14,002,564	10,062,342	71.86
CN107563_S64	11,188,459	9,741,547	87.07	16,125,263	11,551,410	71.64
CN107570_S65	9,737,116	8,396,055	86.23	14,100,495	10,143,547	71.94
CN107574_S66	8,643,594	7,416,113	85.80	12,684,554	8,932,256	70.42
CN107614_S67	9,234,618	7,854,515	85.06	13,206,897	9,541,098	72.24
CN107652_S68	9,868,705	8,381,702	84.93	14,184,729	9,960,279	70.22
CN107844_S69	10,908,866	9,413,332	86.29	15,593,952	10,859,283	69.64
CN107865_S70	10,898,448	9,263,149	85.00	15,345,254	10,919,501	71.16
CN107880_S71	9,584,049	8,144,974	84.98	13,492,227	9,552,331	70.80
CN032634X2_S72	8,584,581	7,369,611	85.85	12,121,970	8,851,597	73.02

Table S3c. Summary of RNA-Seq sequence reads before trimming, after trimming, and after alignment for the samples of the maize and rapa collections.

Sample	Original fastq paired reads	Trimmed fastq paired reads	Percent trimmed paired reads	Total reads in bam file	Total mapped reads in bam file	Percent of mapped reads in bam file
**Maize**						
CN18928_S1	11,526,978	10,233,115	88.78	18,094,255	12,493,571	69.05
CN29710_S2	12,595,144	11,182,567	88.78	18,749,144	12,828,886	68.42
CN29717_S3	11,229,016	9,901,795	88.18	16,835,600	11,512,652	68.38
CN30557_S4	11,224,147	9,979,862	88.91	17,689,675	12,001,761	67.85
CN33926_S5	14,704,874	13,095,615	89.06	22,616,045	15,497,279	68.52
CN35616_S6	15,103,264	13,404,991	88.76	22,429,365	15,367,190	68.51
CN35617_S7	6,021,769	5,323,019	88.40	10,249,116	6,976,682	68.07
CN35622_S8	7,706,456	6,753,930	87.64	12,737,610	8,632,839	67.77
CN35623_S9	13,469,298	11,810,467	87.68	20,312,417	13,764,884	67.77
CN35866_S10	13,387,540	11,975,765	89.45	20,305,440	14,113,395	69.51
CN35947_S11	15,786,581	14,077,402	89.17	23,425,827	15,595,469	66.57
CN36013_S12	5,279,165	4,654,867	88.17	8,960,734	6,097,593	68.05
CN36237_S13	14,316,976	12,782,391	89.28	21,826,425	14,555,684	66.69
CN36260_S14	10,647,980	9,377,341	88.07	16,367,394	11,094,861	67.79
CN36262_S15	8,077,546	7,139,988	88.39	13,285,119	9,095,482	68.46
CN36283_S16	11,090,245	9,659,339	87.10	17,170,560	11,854,290	69.04
CN36286_S17	6,688,203	5,871,283	87.79	10,633,807	7,326,846	68.90
CN36295i101_S18	6,589,851	5,890,205	89.38	11,106,339	7,566,085	68.12
CN36800_S19	6,881,000	6,103,052	88.69	11,492,553	7,958,370	69.25
CN36804_S20	6,314,298	5,633,427	89.22	10,388,454	7,152,366	68.85
CN36825_S21	8,273,156	7,340,688	88.73	12,878,989	8,783,817	68.20
CN36853_S22	13,169,514	11,782,369	89.47	20,305,966	13,897,117	68.44
CN36885_S23	5,419,083	4,822,072	88.98	9,158,275	6,344,987	69.28
CN36886_S24	6,612,276	5,856,063	88.56	10,775,087	7,262,910	67.40
CN36889_S25	10,200,583	9,020,815	88.43	15,378,692	10,421,795	67.77
CN36890_S26	10,357,796	9,144,479	88.29	15,535,240	10,716,243	68.98
CN36931_S27	8,489,627	7,415,342	87.35	13,509,311	9,144,399	67.69
CN36934_S28	10,613,727	9,274,595	87.38	15,805,297	10,906,518	69.01
CN36945_S29	10,516,471	9,246,555	87.92	16,397,465	10,814,702	65.95
CN36952_S30	10,656,953	9,256,748	86.86	16,393,862	11,128,607	67.88
CN36971_S31	10,449,034	9,188,280	87.93	15,987,121	10,667,549	66.73
CN36973_S32	9,533,819	8,232,108	86.35	14,859,778	10,242,847	68.93
CN36982_S33	9,943,401	8,736,913	87.87	15,311,747	10,529,213	68.77
CN36997_S34	9,270,225	8,251,706	89.01	15,469,401	10,411,299	67.30
CN37003_S35	7,519,978	6,667,920	88.67	12,280,279	8,307,011	67.65
CN37038_S36	11,334,406	10,014,784	88.36	17,053,937	11,351,768	66.56
CN37040_S37	8,069,862	7,015,390	86.93	12,811,596	8,859,141	69.15
CN37054_S38	8,853,034	7,819,012	88.32	14,092,001	9,614,648	68.23
CN37063_S39	9,963,490	8,817,353	88.50	15,405,310	10,538,373	68.41
CN37065_S40	11,442,399	10,159,144	88.79	17,346,350	11,777,658	67.90
CN37071_S41	14,571,185	12,333,977	84.65	18,605,615	12,411,190	66.71
CN37080_S42	9,952,679	8,743,709	87.85	15,675,873	10,758,250	68.63
CN37089_S43	13,084,354	11,410,651	87.21	18,347,903	12,442,399	67.81
CN37100_S44	10,705,059	9,463,094	88.40	16,634,573	11,239,222	67.57
CN37115_S45	11,523,976	10,185,001	88.38	17,684,072	11,994,015	67.82
CN37117_S46	11,442,087	10,124,856	88.49	17,511,209	12,013,366	68.60
CN37134_S47	11,264,091	9,987,851	88.67	17,834,436	11,717,492	65.70
CN36295i101x1_S4	10,976,230	9,702,895	88.40	17,243,227	11,625,342	67.42
CN37488_S49	12,187,805	10,742,923	88.14	18,691,822	12,298,841	65.80
CN38968_S50	8,676,697	7,743,412	89.24	13,932,586	9,568,728	68.68
CN38981_S51	9,172,658	8,056,586	87.83	14,018,968	9,340,238	66.63
CN38998_S52	10,442,233	9,221,725	88.31	16,241,547	11,168,884	68.77
CN40135_S53	12,053,427	10,691,544	88.70	18,663,134	12,674,338	67.91
CN40137_S54	12,740,358	11,316,860	88.83	19,133,097	12,955,945	67.71
CN40140_S55	12,538,751	11,046,696	88.10	18,732,838	12,629,726	67.42
CN40162_S56	11,213,984	9,892,821	88.22	17,555,844	12,108,299	68.97
CN40466_S57	10,321,477	9,124,392	88.40	16,383,047	11,277,125	68.83
CN40485_S58	12,299,906	10,778,072	87.63	18,941,649	12,509,662	66.04
CN41083_S59	11,722,247	10,434,757	89.02	17,989,501	12,348,108	68.64
CN42430_S60	8,827,041	7,808,404	88.46	13,996,051	9,618,445	68.72
CN42446_S61	9,625,095	8,481,488	88.12	15,465,710	10,538,229	68.14
CN42449_S62	10,929,910	9,672,357	88.49	17,168,399	11,639,822	67.80
CN42451_S63	11,023,602	9,696,346	87.96	16,853,861	11,553,882	68.55
CN46339_S64	8,528,984	7,573,677	88.80	14,121,622	9,467,475	67.04
CN52040_S65	8,332,730	7,346,216	88.16	13,186,980	9,121,749	69.17
CN52051_S66	13,460,190	11,896,056	88.38	19,988,363	13,621,530	68.15
CN52061_S67	10,427,131	9,292,342	89.12	16,085,633	10,989,239	68.32
CN33815i102_S68	6,877,571	6,138,029	89.25	11,074,556	7,587,895	68.52
CN36285i101_S69	9,401,975	8,318,898	88.48	15,069,403	10,277,396	68.20
CN36882i104_S70	8,768,377	7,779,206	88.72	13,979,908	9,434,641	67.49
CN36990i101_S71	9,409,143	8,306,636	88.28	14,623,622	9,936,785	67.95
CN36295i101x2_S7	10,734,651	9,548,766	88.95	16,951,449	11,455,723	67.58

Sample	Original fastq paired reads	Trimmed fastq paired reads	Percent trimmed paired reads	Total reads in bam file	Total mapped reads in bam file	Percent of mapped reads in bam file
**Rapa**						
CN101878_S1	12,825,015	9,896,078	77.16	14,038,554	9,477,123	67.51
CN101886_S2	11,991,737	9,167,931	76.45	13,689,902	9,000,652	65.75
CN101888_S3	10,476,696	8,094,066	77.26	11,991,968	8,056,468	67.18
CN101892_S4	10,333,480	7,852,870	75.99	11,584,186	7,457,145	64.37
CN101908_S5	10,492,489	7,991,026	76.16	12,399,079	8,392,654	67.69
CN101915_S6	11,206,634	8,651,156	77.20	13,032,111	8,380,539	64.31
CN101917_S7	12,763,973	9,820,974	76.94	12,637,984	8,028,881	63.53
CN101941_S8	11,345,257	8,717,298	76.84	12,575,146	8,153,624	64.84
CN105404_S9	12,084,563	9,409,479	77.86	14,210,733	8,806,470	61.97
CN105412_S10	12,184,907	9,429,976	77.39	13,002,683	8,512,641	65.47
CN107713_S11	7,026,567	5,407,472	76.96	8,451,829	4,999,311	59.15
CN107744_S12	12,580,549	9,756,764	77.55	13,894,456	8,889,453	63.98
CN107748_S13	7,239,005	5,535,257	76.46	9,524,842	6,621,053	69.51
CN107765_S14	10,841,790	8,358,815	77.10	12,093,364	7,967,110	65.88
CN107778_S15	9,103,009	6,983,491	76.72	11,108,145	7,101,765	63.93
CN110921_S16	9,270,859	7,019,841	75.72	10,650,791	6,875,566	64.55
CN110930_S17	10,214,584	7,805,635	76.42	10,196,129	7,189,479	70.51
CN110934_S18	11,397,226	8,854,865	77.69	13,336,768	8,145,777	61.08
CN110946_S19	11,931,464	9,261,483	77.62	11,695,414	7,651,685	65.42
CN110956_S20	7,701,681	5,923,213	76.91	9,134,347	5,684,260	62.23
CN110957_S21	12,581,906	9,715,424	77.22	12,704,696	8,522,239	67.08
CN110958_S22	11,015,945	8,424,010	76.47	12,467,828	8,427,340	67.59
CN110962_S23	9,249,056	7,093,022	76.69	10,575,802	6,966,082	65.87
CN110964_S24	12,142,726	9,394,788	77.37	13,785,799	8,526,685	61.85
CN110971_S25	12,976,356	10,409,690	80.22	14,310,175	9,456,665	66.08
CN110972_S26	12,724,475	10,084,561	79.25	14,065,456	9,250,476	65.77
CN110974_S27	11,912,848	9,517,948	79.90	13,630,926	9,453,274	69.35
CN110999_S28	10,760,718	8,533,970	79.31	12,180,808	8,152,207	66.93
CN111012_S29	14,792,039	11,970,411	80.92	16,842,433	10,937,904	64.94
CN111017_S30	14,112,186	11,241,279	79.66	17,146,280	11,037,532	64.37
CN111023_S31	6,951,561	5,549,157	79.83	9,685,566	6,101,378	62.99
CN111036_S32	8,679,117	6,857,404	79.01	11,750,867	7,283,917	61.99
CN111038_S33	13,458,811	10,706,944	79.55	15,929,380	10,632,625	66.75
CN111042_S34	14,135,010	11,424,475	80.82	15,417,550	10,749,003	69.72
CN111046_S35	14,258,487	11,475,948	80.49	17,422,499	10,518,083	60.37
CN111048_S36	5,540,851	4,438,325	80.10	7,634,247	4,551,748	59.62
CN111059_S37	14,783,512	11,874,909	80.33	16,561,110	11,039,504	66.66
CN111084_S38	8,870,354	6,998,892	78.90	11,282,003	7,844,571	69.53
CN111097_S39	7,257,517	5,679,105	78.25	8,653,098	5,831,418	67.39
CN111107_S40	10,249,960	8,228,349	80.28	11,846,951	8,236,841	69.53
CN111111_S41	6,340,609	4,927,064	77.71	7,195,226	5,055,865	70.27
CN111116_S42	5,838,266	4,626,085	79.24	6,783,179	4,620,718	68.12
CN111139_S43	6,768,405	5,336,037	78.84	8,032,431	5,433,204	67.64
CN111157_S44	6,069,440	4,898,909	80.71	7,815,448	5,266,145	67.38
CN111173_S45	8,592,908	6,876,687	80.03	10,985,961	6,555,209	59.67
CN111177_S46	11,858,840	9,552,634	80.55	13,793,808	9,315,026	67.53
CN111187_S47	5,380,115	4,226,308	78.55	7,597,084	5,174,410	68.11
CN107778x1_S48	7,395,770	5,854,572	79.16	9,593,344	6,309,238	65.77
CN111190_S49	11,413,855	9,194,712	80.56	15,110,218	10,524,528	69.65
CN111195_S50	9,873,379	8,092,682	81.96	12,350,470	7,599,571	61.53
CN111228_S51	10,615,600	8,670,584	81.68	13,587,871	8,328,689	61.30
CN111229_S52	11,840,422	9,634,152	81.37	13,665,338	8,959,737	65.57
CN111232_S53	11,797,364	9,687,769	82.12	14,675,650	8,187,200	55.79
CN111242_S54	11,966,976	9,706,572	81.11	13,896,332	9,005,474	64.80
CN111267_S55	11,446,408	9,340,281	81.60	13,952,431	8,853,536	63.46
CN111272_S56	11,694,310	9,442,099	80.74	13,579,417	8,674,818	63.88
CN111293_S57	10,687,931	8,623,106	80.68	12,043,086	7,632,227	63.37
CN111324_S58	9,728,114	7,843,453	80.63	11,493,829	7,203,961	62.68
CN112919_S59	7,625,941	6,148,220	80.62	9,701,921	5,958,272	61.41
CN112921_S60	10,650,153	8,690,263	81.60	12,612,144	7,426,542	58.88
CN112924_S61	7,446,227	5,959,001	80.03	8,843,774	5,512,964	62.34
CN113058_S62	8,233,292	6,646,069	80.72	9,879,956	6,006,796	60.80
CN115107_S63	8,722,987	6,940,903	79.57	10,421,816	6,389,550	61.31
CN115245_S64	9,829,108	7,836,372	79.73	12,089,314	7,849,462	64.93
CN19060_S65	9,594,833	7,720,672	80.47	12,356,921	7,674,571	62.11
CN35815_S66	9,186,248	7,295,064	79.41	10,163,568	6,572,708	64.67
CN35956_S67	9,490,935	7,656,904	80.68	12,075,827	7,409,089	61.35
CN36094_S68	9,050,121	7,273,207	80.37	11,329,465	7,328,208	64.68
CN44103_S69	10,257,433	8,365,021	81.55	12,840,339	8,532,253	66.45
CN31402_S70	10,466,486	8,432,365	80.57	12,485,000	7,543,454	60.42
CN44108_S71	9,924,344	7,972,163	80.33	11,649,167	6,940,213	59.58
CN107778x2_S72	10,222,132	8,126,817	79.50	12,511,931	7,522,671	60.12

Table S3d. Summary of RNA-Seq sequence reads before trimming, after trimming, and after alignment for the samples of the sunflower collection.

Sample	Original fastq paired reads	Trimmed fastq paired reads	Percent trimmed paired reads	Total reads in bam file	Total mapped reads in bam file	Percent of mapped reads in bam file
**Sunflower**						
CN29474_S1	11,000,195	8,456,981	76.88	11,847,688	8,809,859	74.36
CN29665_S2	11,573,193	9,064,236	78.32	11,715,211	8,827,360	75.35
CN31851_S3	11,577,650	9,008,924	77.81	11,626,875	8,752,062	75.27
CN32248_S4	12,241,265	8,512,717	69.54	9,832,064	7,599,747	77.30
CN32252_S5	11,618,269	9,301,555	80.06	11,827,974	8,632,535	72.98
CN32627_S6	12,257,584	9,388,961	76.60	12,466,332	9,436,733	75.70
CN33282_S7	12,858,877	9,965,577	77.50	12,653,286	9,625,514	76.07
CN33285_S8	12,626,617	9,619,596	76.19	12,790,310	9,657,587	75.51
CN33963_S9	13,432,038	10,532,984	78.42	13,224,500	10,070,124	76.15
CN35597_S10	11,567,473	9,121,607	78.86	11,504,100	8,710,566	75.72
CN36523_S11	11,335,025	8,430,075	74.37	11,428,906	8,630,579	75.52
CN36542_S12	13,310,378	9,722,809	73.05	11,151,328	8,543,063	76.61
CN36543_S13	11,533,841	8,914,633	77.29	11,454,276	8,746,705	76.36
CN36554_S14	11,996,992	9,126,818	76.08	11,468,980	8,664,431	75.55
CN36555_S15	11,821,876	9,084,533	76.85	11,320,651	8,680,010	76.67
CN36564_S16	14,059,829	9,361,386	66.58	9,684,442	7,172,480	74.06
CN36566_S17	8,868,225	7,765,649	87.57	12,070,727	8,650,906	71.67
CN36573_S18	7,363,497	6,311,226	85.71	9,886,342	7,429,641	75.15
CN36574_S19	7,888,256	6,889,828	87.34	11,292,350	8,462,615	74.94
CN36575_S20	7,488,561	6,544,100	87.39	10,253,341	7,748,041	75.57
CN36582_S21	8,789,602	7,762,253	88.31	11,439,214	8,450,336	73.87
CN36602_S22	9,674,536	8,527,276	88.14	12,640,090	9,474,952	74.96
CN36603_S23	9,296,800	8,016,898	86.23	10,753,789	7,949,090	73.92
CN36609_S24	7,622,598	6,601,076	86.60	10,976,545	8,247,246	75.14
CN36613_S25	10,869,119	9,657,494	88.85	14,172,729	10,179,886	71.83
CN36615_S26	9,839,156	8,731,572	88.74	12,009,415	8,766,474	73.00
CN36622_S27	9,007,377	8,056,630	89.44	10,107,733	7,358,060	72.80
CN36641_S28	11,109,980	9,911,157	89.21	13,650,909	10,180,642	74.58
CN36651_S29	10,503,402	9,348,054	89.00	12,980,518	9,679,659	74.57
CN36652_S30	12,579,145	11,274,932	89.63	16,058,059	11,787,164	73.40
CN36676_S31	10,756,930	9,584,720	89.10	13,776,736	10,104,378	73.34
CN36677_S32	11,341,661	10,084,990	88.92	14,571,469	10,825,873	74.30
CN36679_S33	8,550,898	7,601,924	88.90	11,549,304	8,385,272	72.60
CN36686_S34	11,311,508	10,115,487	89.43	14,871,901	11,087,704	74.55
CN36689_S35	9,715,240	8,736,206	89.92	13,057,508	9,695,307	74.25
CN36701_S36	8,696,578	7,768,228	89.33	12,147,509	8,884,392	73.14
CN36703_S37	10,484,903	9,367,589	89.34	13,686,791	10,162,929	74.25
CN36707_S38	9,863,673	8,793,694	89.15	13,163,490	9,669,966	73.46
CN36711_S39	12,567,199	11,172,023	88.90	15,361,653	11,565,574	75.29
CN36719_S40	9,211,393	8,206,218	89.09	12,733,728	9,336,021	73.32
CN39651_S41	7,510,524	6,656,604	88.63	10,591,397	8,007,642	75.61
CN39656_S42	11,693,387	10,459,567	89.45	15,650,248	11,783,176	75.29
CN39658_S43	9,018,654	8,087,087	89.67	12,589,165	9,409,301	74.74
CN39666_S44	12,151,965	10,930,592	89.95	15,852,803	12,057,316	76.06
CN40468_S45	8,388,917	7,530,447	89.77	11,424,471	8,746,665	76.56
CN40469_S46	8,059,812	7,178,161	89.06	11,101,077	8,404,003	75.70
CN42228_S47	10,032,597	8,923,028	88.94	13,962,864	10,512,784	75.29
CN33285x1_S48	9,605,182	8,528,585	88.79	13,641,408	10,326,843	75.70
CN42238_S49	10,737,384	9,524,121	88.70	12,717,644	9,660,158	75.96
CN42239_S50	8,559,990	7,472,598	87.30	12,322,472	9,209,703	74.74
CN42248_S51	9,581,048	8,450,979	88.21	12,942,383	9,464,717	73.13
CN42249_S52	11,346,558	9,940,339	87.61	14,437,543	10,684,564	74.01
CN42253_S53	10,371,931	9,115,147	87.88	13,972,773	10,544,486	75.46
CN42259_S54	8,734,039	7,674,361	87.87	10,421,954	7,823,138	75.06
CN42273_S55	9,531,408	8,403,179	88.16	12,997,688	9,665,625	74.36
CN42279_S56	10,588,588	9,342,433	88.23	14,772,096	11,149,422	75.48
CN42280_S57	13,032,161	11,541,512	88.56	16,422,276	12,307,271	74.94
CN42282_S58	9,497,111	8,367,419	88.10	12,841,472	9,560,504	74.45
CN42284_S59	7,843,059	6,924,330	88.29	10,877,217	8,072,046	74.21
CN42285_S60	12,851,744	11,429,385	88.93	15,349,305	11,109,415	72.38
CN42307_S61	8,982,801	7,928,055	88.26	11,057,069	8,431,750	76.26
CN42333_S62	11,647,537	10,265,521	88.13	13,997,717	10,697,238	76.42
CN42335_S63	11,412,292	9,913,342	86.87	11,386,974	8,645,772	75.93
CN42342_S64	11,051,062	9,755,033	88.27	12,360,582	9,241,662	74.77
CN42754_S65	10,384,069	9,224,740	88.84	14,234,801	10,583,924	74.35
CN42765_S66	12,405,337	11,050,869	89.08	16,056,771	11,970,991	74.55
CN51712_S67	11,638,800	10,334,772	88.80	14,326,720	10,516,831	73.41
CN51717_S68	7,220,188	6,334,427	87.73	9,943,936	7,366,832	74.08
CN51816_S69	11,673,650	10,228,998	87.62	14,694,852	11,135,936	75.78
CN51817_S70	10,194,760	8,916,172	87.46	12,919,237	9,760,338	75.55
CN52559_S71	8,569,656	7,271,235	84.85	11,047,794	8,203,710	74.26
CN33285x2_S72	10,713,680	8,993,044	83.94	14,217,590	10,535,388	74.10

Table S4a. Summary of SNP discovery, SNP annotation, deleterious SNP identification, expressed deleterious genes, deleterious mutation burdens, and adaptive mutation inference in the barley samples.

Barley		SY		RF		GD	
Variants and other estimates	All	10Y	27Y	RF1	RF2	68G	91G
Sample size	72	16	16	24	24	17	17
SNP calling							
Total SNPs	969779	486617	419284	588701	591156	473878	480222
SNP annotation with VEP (most severe consequences)							
Missence_variant (MV)	137450	78808	70836	92689	96404	78994	78222
Proportion of MV in total SNPs	0.1417	0.1620	0.1689	0.1574	0.1631	0.1667	0.1629
Synonymous_variant (SV)	121893	75475	68372	87260	90173	76035	75129
Proportion of SV in total SNPs	0.1257	0.1551	0.1631	0.1482	0.1525	0.1605	0.1564
Splice_acceptor_variant	11428	5730	6876	8562	8472	6980	6758
Splice_donor_variant	14118	7009	5636	6626	6625	5553	5544
Stop_gained	2327	1270	1097	1534	1548	1208	1223
Stop_lost	826	514	450	571	590	495	505
Start_lost	1595	917	806	1049	1072	899	881
Splice_region_variant	20188	10038	9543	12206	12212	9891	9897
Stop_retained_variant	202	118	104	142	156	126	123
Coding_sequence_variant	13	11	9	12	11	11	8
5_prime_UTR_variant	67293	36264	30847	43326	43464	34761	35344
3_prime_UTR_variant	94312	52246	46529	62462	63962	53554	53145
Non_coding_transcript_exon_variant	22534	12874	11252	15331	15695	12654	13027
Intron_variant	135503	57804	46266	73322	71536	53797	56824
Upstream_gene_variant	50850	22141	18474	27766	27673	21344	21512
Downstream_gene_variant	49408	20978	17292	26376	26404	20611	20350
Intergenic_variant	239839	104420	84895	129467	125159	96965	101730
Loss-of-function variant*							
Total count	50684	25596	24512	30690	30675	25152	24931
Proportion	0.0523	0.0526	0.0585	0.0521	0.0519	0.0531	0.0519
SIFT analysis with CT**							
SIFT-deleterious SNPs (SDS)	122250	60137	52089	72850	74510	59349	58485
Proportion of SDS in total SNPs	0.1261	0.1236	0.1242	0.1237	0.1260	0.1252	0.1218
Deleterious_low_confidence SNPs	52746	29692	25464	35371	37242	29905	29908
Tolerated SNPs	515935	305582	269028	359256	373187	305959	304336
Tolerated_low_confidence SNPs	100189	63794	55194	73563	77999	62903	62945
Deleterious SNPs by SIFT+RS							
SDS+RS-filtered SNPs (RSD)	1146	547	493	679	697	543	556
Proportion of RSD in total SNPs	0.00118	0.00112	0.00118	0.00115	0.00118	0.00115	0.00116
Fixed RSD	37	31	42	34	26	37	35
Proportion of fixed RSD in total SNPs	0.000038	0.000064	0.000100	0.000058	0.000044	0.000078	0.000073
Expressed deleterious genes (EDGs)							
Total EDGs	686	359	338	432	448	381	365
Proportion of dSNPs	0.599	0.656	0.686	0.636	0.643	0.702	0.656
Mean TPM/gene/sample***	12.738	13.246	14.791	14.859	12.801	15.687	12.289
Individual mutation burden per dSNP							
Heterozygous (HET)	0.0617	0.0788	0.0885	0.0767	0.0747	0.0733	0.0834
Standard deviation	0.0049	0.0088	0.0066	0.0064	0.0058	0.0057	0.0052
Homozygous (HOM)	0.1143	0.2213	0.2117	0.1787	0.1604	0.2073	0.2169
Standard deviation	0.0105	0.0240	0.0254	0.0185	0.0152	0.0226	0.0167
Total	0.1760	0.3001	0.3002	0.2553	0.2351	0.2806	0.3003
Standard deviation	0.0110	0.0210	0.0245	0.0185	0.0139	0.0229	0.0154
Inference of adaptive mutations							
Mean alpha-dfe	0.9628	0.8275	0.7863	0.869	0.878	0.867	0.794
Standard deviation	0.0026	0.0025	0.0563	0.034	0.008	0.009	0.079

* Loss-of-function variants consist of those variants from the annotation classes of three "Stop_", three "Splice_" and one "Start_lost"

** SIFT-filtered with canonical transcripts

*** TPM for transcripts per million

Table S4b. Summary of SNP discovery, SNP annotation, deleterious SNP identification, expressed deleterious genes, deleterious mutation burdens, and adaptive mutation inference in the wheat samples.

Wheat		SY		RF		GD	
Variants and other estimates	All	20Y	25Y	RF1	RF2	26G	72G
Sample size	72	12	12	18	18	17	17
SNP calling							
Total SNPs	1684050	574519	592005	800988	775282	760633	782540
SNP annotation with VEP (most severe consequences)							
Missence_variant (MV)	213248	81977	87,716	116754	108759	112235	110797
Proportion of MV in total SNPs	0.1266	0.1427	0.1482	0.1458	0.1403	0.1476	0.1416
Synonymous_variant (SV)	239149	100225	105,196	138967	128724	132334	128565
Proportion of SV in total SNPs	0.1420	0.1745	0.1777	0.1735	0.1660	0.1740	0.1643
Splice_acceptor_variant	12677	4531	3,958	5181	5303	4994	5311
Splice_donor_variant	13186	3951	4,584	5822	5938	5522	5899
Stop_gained	1756	560	597	868	798	826	816
Stop_lost	411	169	178	232	230	222	231
Start_lost	242	106	114	144	128	133	137
Splice_region_variant	23101	7062	7132	9476	9595	9018	9522
Stop_retained_variant	251	114	108	145	140	139	132
Coding_sequence_variant	10	5	4	8	7	8	8
5_prime_UTR_variant	49269	22318	37525	28299	27381	26948	26531
3_prime_UTR_variant	87393	36094	22021	50331	47006	48762	47060
Non_coding_transcript_exon_variant	1993	698	672	937	935	870	907
Intron_variant	150759	36541	38070	58068	54902	56476	55744
Upstream_gene_variant	121092	38966	40026	53920	53135	51016	53498
Downstream_gene_variant	108523	32365	34037	47661	45608	45728	46654
Intergenic_variant	660988	208837	210057	284175	286692	265402	290728
Loss-of-function variant*							
Total count	51624	16493	16671	21868	22132	20854	22048
Proportion	0.0307	0.0287	0.0282	0.0273	0.0285	0.0274	0.0282
SIFT analysis with CT*							
SIFT-deleterious SNPs (SDS)	38216	12644	10498	18928	17203	18177	17976
Proportion of SDS in total SNPs	0.0227	0.0220	0.0177	0.0236	0.0222	0.0239	0.0230
Deleterious_low_confidence SNPs	10967	4447	3241	5987	5634	5752	5695
Tolerated SNPs	181720	70733	57856	101907	94444	97687	96282
Tolerated_low_confidence SNPs	35107	16190	11880	20900	20246	20254	20142
Deleterious SNPs by SIFT+RS							
SDS+RS-filtered SNPs (RSD)	5646	1891	2116	2744	2518	2647	2711
Proportion of RSD in total SNPs	0.003353	0.003291	0.003574	0.00343	0.003248	0.00348	0.003464
Fixed RSD	68	89	84	107	96	138	75
Proportion of fixed RSD in total SNPs	0.000040	0.000155	0.000142	0.000134	0.000124	0.000181	0.000096
Expressed deleterious genes (EDGs)							
Total EDGs	915	307	351	458	414	444	433
Proportion of dSNPs	0.162	0.162	0.166	0.167	0.164	0.168	0.160
Mean TPM/gene/sample***	7.516	12.143	6.947	7.142	7.831	9.515	6.913
Individual mutation burden per dSNP							
Heterozygous (HET)	0.0713	0.0866	0.0849	0.0899	0.0838	0.0892	0.0785
Standard deviation	0.0049	0.0053	0.0060	0.0105	0.0048	0.0101	0.0027
Homozygous (HOM)	0.0992	0.2444	0.2355	0.2269	0.2107	0.2659	0.1831
Standard deviation	0.0154	0.0265	0.0209	0.0465	0.0224	0.0577	0.0218
Total	0.1705	0.3309	0.3205	0.3168	0.2945	0.3552	0.2616
Standard deviation	0.0159	0.0286	0.0165	0.0417	0.0202	0.0524	0.0211
Inference of adaptive mutations							
Mean alpha-dfe	0.9480	0.9452	0.9444	0.9296	0.9327	0.9085	0.9170
Standard deviation	0.0027	0.0021	0.0092	0.0046	0.0013	0.0039	0.0045

* Loss-of-function variants consist of those variants from the annotation classes of three "Stop_", three "Splice_" and one "Start_lost"

** SIFT-filtered with canonical transcripts

*** TPM for transcripts per million

Table S4c. Summary of SNP discovery, SNP annotation, deleterious SNP identification, expressed deleterious genes, deleterious mutation burdens, and adaptive mutation inference in the oat samples.

Oat		SY		RF		GD	
Variants and other estimates	All	16Y	21Y	RF1	RF2	84G	96G
Sample size	72	10	10	9	9	16	16
SNP calling							
Total SNPs	2937759	723648	943550	525985	634513	1015427	921113
SNP annotation with VEP (most severe consequences)							
Missence_variant (MV)	604085	155,703	181380	115770	135,069	213996	197,713
Proportion of MV in total SNPs	0.2056	0.2152	0.1922	0.2201	0.2129	0.2107	0.2146
Synonymous_variant (SV)	493824	156,552	216479	114582	136,394	204437	188,361
Proportion of SV in total SNPs	0.1681	0.2163	0.2294	0.2178	0.2150	0.2013	0.2045
Splice_acceptor_variant	18188	5,244	5402	5031	4,873	7169	7,456
Splice_donor_variant	19509	5,346	5727	5408	5,196	7614	7,761
Stop_gained	12587	2,851	2860	2116	2,366	3990	3,746
Stop_lost	2201	696	719	512	604	896	855
Start_lost	1687	374	428	290	358	566	519
Splice_region_variant	36645	9,614	10441	8723	8,630	13298	13,266
Stop_retained_variant	708	203	318	144	179	285	258
Coding_sequence_variant	12	3	2	3	3	4	4
5_prime_UTR_variant	184127	44,219	57791	35121	39,872	64012	58,145
3_prime_UTR_variant	335401	80,385	108835	56206	71,754	110418	102,136
Non_coding_transcript_exon_variant							
Intron_variant	352707	69,293	91281	49302	60,227	105445	92,556
Upstream_gene_variant	306527	70,669	89780	51124	62,311	103135	91,405
Downstream_gene_variant	199899	44,701	58282	30723	39,633	64771	58,315
Intergenic_variant	369651	77,795	113825	50925	67,044	115391	98,617
Loss-of-function variant*							
Total count	91525	24328	25895	22224	22206	33818	33861
Proportion	0.0312	0.0336	0.0274	0.0423	0.0350	0.0333	0.0368
SIFT analysis with CT**							
SIFT-deleterious SNPs (SDS)	165801	32779	33320	25435	28025	47548	45042
Proportion of SDS in total SNPs	0.0564	0.0453	0.0353	0.0484	0.0442	0.0468	0.0489
Deleterious_low_confidence SNPs	49791	11946	13692	9152	10376	16846	15281
Tolerated SNPs	772358	237731	314671	174539	206804	312320	288547
Tolerated_low_confidence SNPs***	NA	NA	NA	NA	NA	NA	NA
Deleterious SNPs by SIFT+RS							
SDS+RS-filtered SNPs (RSD)	83695	15132	14634	12041	12963	22632	21559
Proportion of RSD in total SNPs	0.028489	0.020911	0.015510	0.022892	0.020430	0.022288	0.023405
Fixed RSD	215	320	287	233	307	312	251
Proportion of fixed RSD in total SNPs	0.000073	0.000442	0.000304	0.000443	0.000484	0.000307	0.000272
Expressed deleterious genes (EDGs)							
Total EDGs	5978	1770	1790	1437	1529	2433	2396
Proportion of dSNPs	0.071	0.117	0.122	0.119	0.118	0.108	0.111
Mean TPM/gene/sample****	3.797	4.679	5.180	4.625	4.314	4.254	4.617
Individual mutation burden per dSNP							
Heterozygous (HET)	0.0577	0.1407	0.1355	0.1575	0.1489	0.1202	0.1217
Standard deviation	0.0044	0.0088	0.0075	0.0123	0.0119	0.0091	0.0068
Homozygous (HOM)	0.0117	0.0858	0.0692	0.0805	0.0884	0.0522	0.0474
Standard deviation	0.0026	0.0100	0.0152	0.0161	0.0299	0.0135	0.0108
Total	0.0694	0.2266	0.2047	0.2380	0.2373	0.1723	0.1691
Standard deviation	0.0058	0.0157	0.0194	0.0221	0.0408	0.0209	0.0154
Inference of adaptive mutations							
Mean alpha-dfe	0.95580	0.9013	0.3577	0.8885	0.8832	0.9268	0.9143
Standard deviation	0.01220	0.0052	0.0785	0.0042	0.0102	0.0031	0.0043

* Loss-of-function variants consist of those variants from the annotation classes of three "Stop_", three "Splice_" and one "Start_lost"

** SIFT-filtered with canonical transcripts

*** NA=not available

**** TPM for transcripts per million

Table S4d. Summary of SNP discovery, SNP annotation, deleterious SNP identification, expressed deleterious genes, deleterious mutation burdens, and adaptive mutation inference in the soybean samples.

Soybean		SY		RF		GD	
Variants and other estimates	All	10Y	20Y	RF1	RF2	49G	86G
Sample size	72	15	15	14	14	15	15
SNP calling							
Total SNPs	509404	252867	239457	251558	254385	256696	253910
SNP annotation with VEP (most severe consequences)							
Missence_variant (MV)	88628	51041	47433	51030	54008	51373	52037
Proportion of MV in total SNPs	0.1740	0.2018	0.1981	0.2029	0.2123	0.2001	0.2049
Synonymous_variant (SV)	87298	54128	51459	54849	58143	55046	55199
Proportion of SV in total SNPs	0.1714	0.2141	0.2149	0.2180	0.2286	0.2144	0.2174
Splice_acceptor_variant	11720	5283	4889	4764	4614	5249	5119
Splice_donor_variant	9582	4576	4223	4172	4068	4548	4427
Stop_gained	1002	488	456	483	501	504	496
Stop_lost	205	120	115	123	128	123	121
Start_lost	137	76	70	77	88	80	78
Splice_region_variant	20047	8816	7975	7907	7796	8700	8610
Stop_retained_variant	119	71	58	64	75	67	78
Coding_sequence_variant	2	1	1	1	1	1	1
5_prime_UTR_variant	32575	15287	14437	15427	15948	15711	15303
3_prime_UTR_variant	38905	20656	17963	21260	22494	20580	21180
Non_coding_transcript_exon_variant	393	210	228	250	226	214	224
Intron_variant	90788	35945	33513	35320	33717	36353	35900
Upstream_gene_variant	37956	17668	17512	17651	16799	18106	17339
Downstream_gene_variant	24633	11000	10855	10857	10622	11365	10919
Intergenic_variant	65414	27501	28270	27323	25157	28676	26879
Loss-of-function variant*							
Total count	42812	19430	17786	17590	17270	19271	18929
Proportion	0.0840	0.0768	0.0743	0.069924	0.067889	0.0751	0.07455
SIFT analysis with CT**							
SIFT-deleterious SNPs (SDS)	14482	7035	6350	6963	7503	7073	7135
Proportion of SDS in total SNPs	0.0284	0.0278	0.0265	0.0277	0.0295	0.0276	0.0281
Deleterious_low_confidence SNPs	4052	2124	1911	2081	2259	2115	2171
Tolerated SNPs	158321	96330	90875	96537	102159	97552	98243
Tolerated_low_confidence SNPs***	NA	NA	NA	NA	NA	NA	NA
Deleterious SNPs (dSNPs) by SIFT+RS							
SDS+RS-filtered SNPs (RSD)	749	349	319	336	382	338	350
Proportion of RSD in total SNPs	0.00147	0.00138	0.00133	0.00134	0.00150	0.00132	0.00138
Fixed RSD	12	19	18	16	22	20	16
Proportion of fixed RSD in total SNPs	0.000024	0.000075	0.000075	0.000064	0.000086	0.000078	0.000063
Expressed deleterious genes (EDGs)							
Total EDGs	195	122	117	98	112	96	105
Proportion of dSNPs	0.260	0.350	0.367	0.292	0.293	0.284	0.300
Mean TPM/gene/sample****	8.881	10.603	10.909	11.440	12.213	11.269	11.977
Individual mutation burden per dSNP							
Heterozygous (HET)	0.1297	0.1671	0.1880	0.1607	0.1581	0.1711	0.1565
Standard deviation	0.0088	0.0071	0.0166	0.0127	0.0107	0.0132	0.0110
Homozygous (HOM)	0.1030	0.2108	0.2219	0.2145	0.2139	0.2321	0.2194
Standard deviation	0.0123	0.0316	0.0234	0.0241	0.0260	0.0287	0.0281
Total	0.2326	0.3778	0.4100	0.3752	0.3720	0.4032	0.3758
Standard deviation	0.0155	0.0325	0.0223	0.0274	0.0275	0.0316	0.0269
Inference of adaptive mutations							
Mean alpha-dfe	0.9417	0.9356	0.9309	0.9328	0.9332	0.9466	0.9416
Standard deviation	0.0056	0.0058	0.0054	0.0049	0.0051	0.0036	0.0049

* Loss-of-function variants consist of those variants from the annotation classes of three "Stop_", three "Splice_" and one "Start_lost"

** SIFT-filtered with canonical transcripts

*** NA=not available

**** TPM for transcripts per million

Table S4e. Summary of SNP discovery, SNP annotation, deleterious SNP identification, expressed deleterious genes, deleterious mutation burdens, and adaptive mutation inference in the maize samples.

Maize		SY		RF		GD	
Variants and other estimates	All	7Y	29Y	RF1	RF2	70G	94G
Sample size	72	18	18	18	18	15	15
SNP calling							
Total SNPs	2271978	1313000	1285019	1280451	1273786	1119720	1251884
SNP annotation with VEP (most severe consequences)							
Missence_variant (MV)	301092	202469	202300	198721	198880	181735	193231
Proportion of MV in total SNPs	0.1325	0.1542	0.1574	0.1552	0.1561	0.1623	0.1544
Synonymous_variant (SV)	322549	234581	235109	230933	230579	214879	223854
Proportion of SV in total SNPs	0.1420	0.1787	0.1830	0.1804	0.1810	0.1919	0.1788
Splice_acceptor_variant	12769	6401	6642	6648	6547	5757	6240
Splice_donor_variant	10418	5642	5677	5712	5700	5050	5484
Stop_gained	4750	2736	2639	2612	2607	2247	2643
Stop_lost	1106	729	722	713	729	654	690
Start_lost	1110	739	707	714	738	636	692
Splice_region_variant	41202	21528	21149	21342	21114	18355	20829
Stop_retained_variant	530	366	361	355	351	329	343
Coding_sequence_variant	NA	NA	NA	NA	NA	NA	NA
5_prime_UTR_variant	159755	95356	93225	92997	91932	81274	92232
3_prime_UTR_variant	203428	132371	128252	127617	126314	113556	125715
Non_coding_transcript_exon_variant	8430	4672	4424	4557	4586	3897	4529
Intron_variant	425272	208333	199022	202654	197408	167176	200435
Upstream_gene_variant	256597	131318	125772	126296	126711	107072	123667
Downstream_gene_variant	175515	88410	86722	86684	85313	73175	83994
Intergenic_variant	347443	177341	172287	171891	174268	143925	167302
Loss-of-function variant*							
Total count	71885	38141	37897	38096	37786	33028	36921
Proportion	0.0316	0.0290	0.0295	0.0298	0.0297	0.0295	0.0295
SIFT analysis with CT**							
SIFT-deleterious SNPs (SDS)	3614	2159	2121	2099	2121	1894	2061
Proportion of SDS in total SNPs	0.0016	0.0016	0.0017	0.0016	0.0017	0.0017	0.0016
Deleterious_low_confidence SNPs	1241	723	725	718	708	635	684
Tolerated SNPs	8867	5154	5040	5028	5020	4410	4886
Tolerated_low_confidence SNPs***	NA	NA	NA	NA	NA	NA	NA
Deleterious SNPs by SIFT+RS							
SDS+RS-filtered SNPs (RSD)	125	82	91	88	86	83	82
Proportion of RSD in total SNPs	0.000055	0.000062	0.000071	0.000069	0.000068	0.000074	0.000066
Fixed RSD	5	5	2	3	8	5	6
Proportion of fixed RSD in total SNPs	0.000002	0.000004	0.000002	0.000002	0.000006	0.000004	0.000005
Expressed deleterious genes (EDGs)							
Total EDGs	21	14	20	14	14	13	11
Proportion of dSNPs	0.168	0.171	0.220	0.159	0.163	0.157	0.134
Mean TPM/gene/sample****	9.868	5.843	14.567	16.149	5.898	20.459	8.224
Individual mutation burden per dSNP							
Heterozygous (HET)	0.1630	0.1913	0.1919	0.1909	0.1873	0.2045	0.1975
Standard deviation	0.0174	0.0245	0.0163	0.0207	0.0246	0.0181	0.0223
Homozygous (HOM)	0.1940	0.2340	0.2516	0.2661	0.2758	0.2878	0.2486
Standard deviation	0.0222	0.0332	0.0295	0.0389	0.0314	0.0341	0.0342
Total	0.3569	0.4253	0.4435	0.4570	0.4632	0.4923	0.4461
Standard deviation	0.0200	0.0327	0.0259	0.0290	0.0301	0.0314	0.0196
Inference of adaptive mutations							
Mean alpha-dfe	0.9610	0.8293	0.8080	0.8533	0.8502	0.8138	0.7645
Standard deviation	0.0010	0.0171	0.0247	0.0125	0.0054	0.0279	0.0351

* Loss-of-function variants consist of those variants from the annotation classes of three "Stop_", three "Splice_" and one "Start_lost"

** SIFT-filtered with canonical transcripts

*** NA=not available

**** TPM for transcripts per million

Table S4f. Summary of SNP discovery, SNP annotation, deleterious SNP identification, expressed deleterious genes, deleterious mutation burdens, and adaptive mutation inference in the rapa samples.

Rapa		SY		RF		GD	
Variants and other estimates	All	5Y	16Y	RF1	RF2	69G	96G
Sample size	72	20	20	15	15	12	12
SNP calling							
Total SNPs	1662592	1067846	1124953	910825	955119	908587	842310
SNP annotation with VEP (most severe consequences)							
Missence_variant (MV)	348304	234180	249634	200506	210691	206843	188940
Proportion of MV in total SNPs	0.2095	0.2193	0.2219	0.2201	0.2206	0.2277	0.2243
Synonymous_variant (SV)	667283	491681	527411	429511	450461	459824	417271
Proportion of SV in total SNPs	0.4014	0.4604	0.4688	0.4716	0.4716	0.5061	0.4954
Splice_acceptor_variant	13080	6528	6730	5651	5256	4472	4766
Splice_donor_variant	11316	5858	6127	5224	4901	4391	4539
Stop_gained	2405	1420	1458	1133	1210	1062	1017
Stop_lost	354	195	226	170	175	182	152
Start_lost	295	162	180	140	141	134	124
Splice_region_variant	37940	17999	18580	15290	14902	11786	12354
Stop_retained_variant	637	444	459	387	401	392	360
Coding_sequence_variant	5	4	5	4	4	5	4
5_prime_UTR_variant*	NA	NA	NA	NA	NA	NA	NA
3_prime_UTR_variant*	NA	NA	NA	NA	NA	NA	NA
Non_coding_transcript_exon_variant	608	319	338	239	261	187	232
Intron_variant	175604	80982	81256	65181	68191	49430	50689
Upstream_gene_variant	270292	154459	158062	127887	134758	117925	112474
Downstream_gene_variant	91152	51949	52518	42539	45115	38648	36109
Intergenic_variant	43317	21666	21969	16963	18652	13306	13279
Loss-of-function variant*							
Total count	66027	32606	33760	27995	26986	22419	23312
Proportion	0.0397	0.0305	0.0300	0.0307	0.0283	0.0247	0.0277
SIFT analysis with CT**							
SIFT-deleterious SNPs (SDS)	35860	21747	22884	17993	19049	18019	16643
Proportion of SDS in total SNPs	0.0216	0.0204	0.0203	0.0198	0.0199	0.0198	0.0198
Deleterious_low_confidence SNPs	39603	24110	24947	20007	21065	18959	18062
Tolerated SNPs	830203	603153	648319	526145	551981	561995	509077
Tolerated_low_confidence SNPs*	NA	NA	NA	NA	NA	NA	NA
Deleterious SNPs by SIFT+RS							
SDS+RS-filtered SNPs (RSD)	2219	1277	1420	1095	1137	1109	1007
Proportion of RSD in total SNPs	0.001335	0.001196	0.001262	0.001202	0.001190	0.001221	0.001196
Fixed RSD	42	45	35	33	43	39	40
Proportion of fixed RSD in total SNPs	0.000025	0.000042	0.000031	0.000036	0.000045	0.000043	0.000047
Expressed deleterious genes (EDGs)							
Total EDGs	728	491	531	360	365	373	327
Proportion of dSNPs	0.328	0.384	0.374	0.329	0.321	0.336	0.325
Mean TPM/gene/sample****	25.313	24.907	21.158	31.508	30.662	35.549	32.156
Individual mutation burden per dSNP							
Heterozygous (HET)	0.0770	0.1037	0.1025	0.1225	0.1005	0.1061	0.1267
Standard deviation	0.0137	0.0255	0.0185	0.0280	0.0305	0.0254	0.0152
Homozygous (HOM)	0.1007	0.1600	0.1491	0.1889	0.2022	0.1802	0.1917
Standard deviation	0.0204	0.0398	0.0311	0.0500	0.0462	0.0342	0.0308
Total	0.1778	0.2637	0.2516	0.3114	0.3026	0.2863	0.3184
Standard deviation	0.0139	0.0241	0.0184	0.0329	0.0201	0.0189	0.0312
Inference of adaptive mutations							
Mean alpha-dfe	0.9696	0.8204	0.7891	0.7922	0.8292	0.7724	0.8057
Standard deviation	0.0265	0.0068	0.0184	0.0048	0.0052	0.0197	0.0243

* NA=not available

** Loss-of-function variants consist of those variants from the annotation classes of three "Stop_", three "Splice_" and one "Start_lost"

*** SIFT-filtered with canonical transcripts

**** TPM for transcripts per million

Table S4g. Summary of SNP discovery, SNP annotation, deleterious SNP identification, expressed deleterious genes, deleterious mutation burdens, and adaptive mutation inference in the sunflower samples.

Sunflower		SY		RF		GD	
Variants and other estimates	All	6Y	27Y	RF1	RF2	50G	86G
Sample size	72	15	15	27	27	14	14
SNP calling							
Total SNPs	1708775	799806	812395	1096036	1099504	796036	767825
SNP annotation with VEP (most severe consequences)							
Missence_variant (MV)	208707	122503	121717	154867	161793	123886	119656
Proportion of MV in total SNPs	0.1221	0.1532	0.1498	0.1413	0.1472	0.1556	0.1558
Synonymous_variant (SV)	342665	220348	217891	272666	290266	225512	217202
Proportion of SV in total SNPs	0.2005	0.2755	0.2682	0.2488	0.2640	0.2833	0.2829
Splice_acceptor_variant	14557	6599	6716	9487	8578	6114	5879
Splice_donor_variant	10545	5082	5150	6894	6212	4739	4483
Stop_gained	2402	1270	1265	1579	1559	1275	1225
Stop_lost	559	326	307	325	299	309	295
Start_lost	432	218	231	295	286	220	213
Splice_region_variant	31360	13601	13939	19738	18199	12756	12123
Stop_retained_variant	589	339	360	316	332	360	332
Coding_sequence_variant	115	61	61	90	88	67	66
5_prime_UTR_variant	81915	41428	41980	47939	49247	41790	40237
3_prime_UTR_variant	132718	71108	72901	86404	86377	70422	67624
Non_coding_transcript_exon_variant	394	261	245	302	290	217	260
Intron_variant	273195	97334	101824	153655	147561	94739	91630
Upstream_gene_variant	124693	48461	49863	72417	70674	47076	45086
Downstream_gene_variant	100736	37216	37975	56847	54898	35486	35160
Intergenic_variant	383193	133651	139970	212215	202845	131068	126354
Loss-of-function variant*							
Total count	60444	27435	27968	38634	35465	25773	24550
Proportion	0.0354	0.0343	0.0344	0.0352	0.0323	0.0324	0.0320
SIFT analysis with CT**							
SIFT-deleterious SNPs (SDS)	26182	13864	13869	17873	18589	14013	13627
Proportion of SDS in total SNPs	0.0153	0.0173	0.0171	0.0163	0.0169	0.0176	0.0177
Deleterious_low_confidence SNPs	31550	17588	17485	22343	22978	17713	17010
Tolerated SNPs	493143	309590	306044	371788	394039	315352	304250
Tolerated_low_confidence SNPs***	NA	NA	NA	NA	NA	NA	NA
Deleterious SNPs by SIFT+RS							
SDS+RS-filtered SNPs (RSD)	1180	608	625	775	834	629	597
Proportion of RSD in total SNPs	0.000691	0.000760	0.000769	0.000707	0.000759	0.000790	0.000778
Fixed RSD	27	31	42	31	27	33	24
Proportion of fixed RSD in total SNPs	0.000016	0.000039	0.000052	0.000028	0.000025	0.000041	0.000031
Expressed deleterious genes (EDGs)							
Total EDGs	203	108	97	95	92	76	65
Proportion of dSNPs	0.172	0.178	0.155	0.123	0.110	0.121	0.109
Mean TPM/gene/sample****	5.457	6.087	6.282	7.054	8.042	7.719	7.469
Individual mutation burden per dSNP							
Heterozygous (HET)	0.0923	0.1295	0.1379	0.1255	0.0990	0.1224	0.1198
Standard deviation	0.0110	0.0186	0.0203	0.0169	0.0117	0.0120	0.0119
Homozygous (HOM)	0.1231	0.1967	0.2247	0.1877	0.1663	0.2202	0.2086
Standard deviation	0.0118	0.0169	0.0186	0.0170	0.0177	0.0211	0.0242
Total	0.2154	0.3262	0.3626	0.3132	0.2654	0.3425	0.3284
Standard deviation	0.0106	0.0168	0.0153	0.0133	0.0155	0.0168	0.0214
Inference of adaptive mutations							
Mean alpha-dfe	0.9709	0.8989	0.8886	0.8973	0.8781	0.8841	0.8729
Standard deviation	0.0010	0.0026	0.0056	0.0050	0.0062	0.0045	0.0057

* Loss-of-function variants consist of those variants from the annotation classes of three "Stop_", three "Splice_" and one "Start_lost"

** SIFT-filtered with canonical transcripts

*** NA=not available

**** TPM for transcripts per million

Table S5a. Composition of 12 reference genome sequences used for GERP++ 'rejected substitution' (RS) scoring for identification of deleterious SNPs in the samples of seven germplasm collections and outgroups used for ancestral sequence reconstruction for each crop.

					Composition of 12 reference genome sequences for GERP++ RS scoring for a collection of						
Species	Reference genome sequence file name	Size[a]	Source[b]	Ref.[c]	Barley	Wheat	Soybean	Maize	Rapa	Sunflower	Oat
Barley	Hordeum_vulgare.Hv_IBSC_PGSB_v2.dna_rm.toplev	4,833,791,107	EP 40	1		●	●	●	●	●	●
Wheat	Triticum_aestivum.IWGSC.dna_rm.toplevel.fa.gz	14,547,261,565	EP 41	2	●		●	●	●	●	●
Soybean	Glycine_max.Glycine_max_v2.0.dna_rm.toplevel.fa.	978,416,860	EP 41	3	●	●		●	●	●	●
Maize	Zea_mays.B73_RefGen_v4.dna_rm.toplevel.fa.gz	2,134,373,047	EP 42	4	●	●	●		●	●	●
Rapa	Brassica_rapa.Brapa_1.0.dna_rm.toplevel.fa.gz	283,823,632	EP 42	5	●	●	●	●		●	●
Sunflower	Helianthus_annuus.HanXRQr1.0.dna_rm.toplevel.fa.	3,027,844,945	EP 42	6	●	●	●	●	●		
Banana	Musa_acuminata.MA1.dna_rm.toplevel.fa.gz	472,960,417	EP 40	7	●	●	●	●	●	●	●
Bean	Phaseolus_vulgaris.PhaVulg1_0.dna_rm.toplevel.fa.	521,076,696	EP 40	8	●	●	●	●	●	●	●
Brome	Brachypodium_distachyon.v1.0.dna_rm.toplevel.fa.g	271,923,306	EP 39	9	●	●	●	●	●	●	●
Grape	Vitis_vinifera.IGGP_12x.dna_rm.toplevel.fa.gz	486,265,422	EP 40	10	●	●	●	●	●	●	●
Indica	Oryza_indica.ASM465v1.dna_rm.toplevel.fa.gz	427,004,890	EP 40	11	●	●	●	●	●	●	●
Millet	Setaria_italica.JGIv2.0.dna_rm.toplevel.fa.gz	405,737,341	EP 39	12	●	●	●	●	●	●	●
Thale	Arabidopsis_thaliana.TAIR10.dna_rm.toplevel.fa.gz	119,146,348	EP 40	13	●	●	●	●	●	●	●
Oat	Asativa_sang_pseudomolecules_0.zip	11,195,919,165	GG	14							
Outgroup1[d]					Brome	Brome	Bean	Millet	Thale	Soybean	Brome
Outgroup2[d]					Indica	Indica	Thale	Indica	Grape	Thale	Indica

[a]Genome size, in basepair, from the published and updated version of the sequence available in the host database at the time of analysis.
[b]Host database and sequence release verison. EP = Ensembl Plants (https:// http://plants.ensembl.org). Also, barley-split reference geome sequence used for barley analysis was obtained from https://doi.ipk-gatersleben.de/DOI/a056fc05-2530-4122-8cc1-98cf265b2f06/ef4f19fc-8f1a-477c-a4b5-332d2eee2a14/2. GG for https://wheat.pw.usda.gov/GG3/content/avena-sang-download.
[c]References: (1) Mascher et al. 2017. Nature 544:427-433; (2) Schmutz et al. 2010. Nature 463:178-183; (3) Badouin et al. 2017. Nature 546:148-152; (4) BRGSPC 2011. Nature Genetics 43:1032-1039; (5) IWGSC 2018. Science 361:eaar7191; (6) Schnable et al. 2009. Science 326:1112-1115; (7) D'Hont et al. 2012. Nature 488:213-217; (8) Vlasova et al. 2016. Genome Biology.17:32; (9) IBI 2010. Nature 463:763-768; (10) Jaillon et al. 2007. Nature 449:463-467; (11) Yu et al. 2002. Science 296:79-92; (12) Bennetzen et al. 2012. Nat. Biotechnol. 30:555-561; (13) AGI 2000. Nature 408:796-815; and (14) Kamal et al. 2022. Nature 606:113–119.
[d]Two outgroups were used for ancestral sequence reconstruction for each crop

Table S5b. Frequency distributions of GERP++ RS values for seven crop species inferred from the alignments of 12 other reference genome sequences (Table S5a).

RS value	Barley	Wheat	Oat	Soybean	Maize	Rapa	Sunflower
0.3	15009	63178	818579	86588	15009	79420	198361
0.6	2166117	5417782	1137694	140272	2166117	104041	768209
0.9	1579172	14869241	11144300	180505	1579172	104933	955423
1.2	257235	7720284	7588222	114418	257235	104572	357044
1.5	91301	145511	1693521	127475	91301	172643	1648
1.8	24443	887255	2947359	309799	24443	364810	0
2.1	8444	70506	3773374	639677	8444	396709	0
2.4	1881	69426	2673724	564976	1881	294756	0
2.7	0	39212	3357173	279543	0	527954	0
3.0	0	2257	2314824	232967	0	275267	0
3.3	0	3181	2054879	94141	0	153113	0
3.6	0	1595	1613985	66740	0	83293	0
3.9	0	109	1280742	21383	0	44996	0
4.2	0	639	769447	14572	0	17252	0
4.5	0	262	702251	4776	0	8572	0
4.8	0	277	367317	1272	0	1959	0
5.1	0	134	383879	243	0	1202	0
>5.1	0	590364	0	0	0	0	0

Table S5c. The distributions of GERP++ RS sites and dSNP counts per chromosome detected in the assayed samples of the seven collections.

Barley			Wheat			Oat			Soybean			Maize			Rapa			Sunflower		
Chr	RS	dSNP	Chr	RS	dSNP	Chr	RS	dSNP	Chr	RS	dSNP	Chr	RS	dSNP	Chr	RS	dSNP	Chr	RS	dSNP
chr1H	553784	136	1A	1276956	321	chr1A	1904915	3337	1	148273	34	1	794711	18	A01	296246	228	1	144915	34
chr2H	578942	162	1B	1294854	360	chr1C	1121197	2486	2	239307	66	2	694544	24	A02	308031	244	2	113559	46
chr3H	618997	192	1D	1292327	134	chr1D	2576904	4666	3	173272	38	3	751595	22	A03	358414	302	3	155878	90
chr4H	620878	142	2A	1626462	372	chr2A	2139701	3307	4	246292	64	4	665414	12	A04	152560	100	4	158941	95
chr5H	626727	182	2B	1608477	454	chr2C	2177962	5105	5	191529	42	5	669467	5	A05	231020	211	5	160493	80
chr6H	543694	134	2D	1620722	279	chr2D	2579453	4505	6	132016	36	6	469962	10	A06	361824	314	6	107752	68
chr7H	600580	198	3A	1525878	274	chr3A	1511942	2331	7	174839	57	7	248526	5	A07	276066	243	7	84318	43
			3B	1543941	360	chr3C	2048308	4722	8	184474	41	8	300235	10	A08	188521	165	8	117075	86
			3D	1539300	173	chr3D	1822210	3083	9	160168	44	9	291234	4	A09	352915	256	9	173746	93
			4A	1450736	264	chr4A	2694711	4317	10	232463	77	10	303731	15	A10	209895	156	10	166986	90
			4B	1390720	290	chr4C	2584597	5879	11	146185	22							11	106202	57
			4D	1365387	149	chr4D	2126228	3763	12	120576	27							12	128644	51
			5A	1566919	322	chr5A	2403742	3820	13	223808	55							13	124746	53
			5B	1508440	316	chr5C	2333645	5700	14	78148	15							14	154694	92
			5D	1520784	170	chr5D	2395533	4199	15	82173	30							15	132204	77
			6A	1174089	242	chr6A	2351464	3716	16	58796	13							16	118411	56
			6B	1191169	291	chr6C	2354385	5617	17	86959	24							17	132121	69
			6D	1152141	143	chr6D	1352664	2019	18	54650	20									
			7A	1337369	326	chr7A	1991125	3016	19	72445	25									
			7B	1245255	259	chr7C	1956456	4663	20	72974	19									
			7D	1354365	147	chr7D	2194128	3444												

Table S6. Estimates of deleterious base-substitution mutations per sample (dBSMs) for the samples of the seven collections. The standard deviation (SD) estimates for dSNPs and fixed dSNPs were generated from 10 jackknife sub-samples of the 70 assayed collection samples.

Collection	Genome size in basepair	dSNPs(SD)	fixed dSNPs(SD)	dBSMs (x 10^{-8}) dSNPs(SD)	dBSMs (x 10^{-8}) fixed dSNPs(SD)
Barley	4,833,791,107	1146(5.2)	37(0.5)	0.3293(0.0015)	0.0107(0.0002)
Wheat	14,547,261,565	5646(25.3)	68(2.2)	0.5391(0.0025)	0.0065(0.0003)
Oat	11,195,919,165	83695(82.1)	215(3.1)	10.3826(0.0267)	0.0102(0.0004)
Soybean	978,416,860	749(2.4)	12(0.3)	1.0633(0.0035)	0.0171(0.0005)
Maize	2,134,373,047	125(0.8)	5(0.3)	0.0814(0.0006)	0.0033(0.0003)
Rapa	283,823,632	2219(6.0)	42(1.1)	10.8587(0.0292)	0.2056(0.0052)
Sunflower	3,027,844,945	1180(2.8)	27(0.6)	0.5413(0.0013)	0.0124(0.0003)

Table S7. Inferences of the changes in deleterious base-substitution mutations per sample per year (dBSMsy) based on the estimation of dSNPs (dS) and fixed dSNPs (fdS) from 10 jackknife sub-samples of the SY groups for each collection (see Table S2). Each SY group pair reflects the difference in the years of storage (YS) in the genebank since the last regeneration, while SY2 samples have more years of storage than SY1 samples. The standard deviations (SD) of the estimates are presented in parenthesis, and the results for significant tests of differences for each SY group pair are given in Table S9 below. Sample size (SZ) for each SY group pair is also shown. The results show some heterogeneity of the changes in dBSMsy with respect to dS and fdS under variable years of storage among the seven collections.

	Genome size	dS		fdS		Difference				Change in dBSMsy (x 10^{-10})	
Collection	in basepair	SY1	SY2	SY1	SY2	YS	dS	fdS	SZ	dS	fdS
Barley	4,833,791,107	529.9(6.6)	473.6(13.5)	40(1.9)	48.3(2.5)	17	-56	8.3	16	-0.428	0.063
Wheat	14,547,261,565	1802.4(34.6)	2559.1(17.9)	124.8(7.2)	109.4(2.8)	5	757	-15.4	12	8.669	-0.176
Oat	11,195,919,165	13974.5(309.7)	13467.1(408.7)	334.3(10.2)	295.6(19.4)	5	-507	-38.7	10	-9.064	-0.691
Soybean	978,416,860	336.4(6.0)	307.6(5.3)	19.4(1.2)	17.9(0.6)	21	-29	-1.5	15	-0.934	-0.049
Maize	2,134,373,047	81.0(0.9)	89.7(0.7)	5.2(0.5)	2.5(0.8)	22	8.7	-2.7	18	0.103	-0.032
Rapa	283,823,632	1243.2(10.7)	1394.5(7.9)	44.9(1.5)	35.7(1.5)	11	151	-9.2	20	24.231	-1.473
Sunflower	3,027,844,945	590.6(5.8)	607.6(4.7)	30.8(1.3)	40.1(2.7)	21	17	9.3	15	0.178	0.098

Table S8a. Summary of gene ontological (GO) analyses of all dSNPs detected in each storage year (SY) group of the seven collections. Biological processes in bold represent the most common ones across seven SY group pairs and biological processes in italics represent the ones unique within each SY group pair. Several major biological processes such as protein phosphorylation, organic substance metabolism, responses to chemical, stress, and stimulus were identified across seven SY group pairs and some distinct biological processes for each SY group pair.

Group	No. of dSNPs	No. of unique genes	No. of GO terms	No. of Bio_Pro	Top 10 Major Bio_Pro
barley 10Y	547	241	1003	168	*Response to chemical, anatomical structure development, cellular lipid metabolism, macromolecule localization,* regulation of biological quality, *nucleic acid phosphodiester bond hydrolysis, cellular metabolism, developmental process, multicellular organismal process, reproduction/reproductive process*
barley 27Y	493	195	447	148	***Protein phosphorylation*, *organic substance metabolism*,** *carbohydrate derivative biosynthesis, organic substance transport,* regulation of biological quality, ***metabolism*, *cellular process*,** *response to stress,* ***nitrogen compound metabolism***, *amino acid activation*
wheat 20Y	1891	839	350	125	**Protein phosphorylation**, **organic substance metabolism**, *lipid metabolism*, organic substance transport, **metabolism**, **cellular process**, organic substance catabolism, response to stress, **nitrogen compound metabolism**, regulation of biological quality
wheat 25Y	2630	1750	458	157	**Protein phosphorylation**, **organic substance metabolism, metabolism**, organic substance catabolism, **cellular process**, *carbohydrate derivative biosynthesis*, organic substance transport, regulation of biological quality, response to stress, **nitrogen compound metabolism**
oat 16Y	15132	10835	2501	1610	**Nitrogen compound metabolism, metabolism**, biosynthesis, **cellular process**, protein metabolism, cellular nitrogen compound metabolism, macromolecule metabolism, cellular metabolism, **organic substance metabolism**, organonitrogen compound metabolism
oat 21Y	14634	11133	2552	1658	**Nitrogen compound metabolism**, **metabolism**, biosynthesis, **cellular process**, protein metabolism, cellular nitrogen compound metabolism, macromolecule metabolism, cellular metabolism, **organic substance metabolism**, organonitrogen compound metabolism
soybean 10Y	349	189	139	53	***Protein phosphorylation***, **organic substance metabolism**, **metabolism**, **cellular process**, **nitrogen compound metabolism**, *negative regulation of biological process, response to stress, biosynthesis,* biological regulation, *cell cycle*
soybean 20Y	319	157	110	39	**Organic substance metabolism**, *dephosphorylation,* **metabolism**, **cellular process**, *RNA phosphodiester bond hydrolysis,* **nitrogen compound metabolism**, *transport,* biological regulation, *response to chemical, response to stimulus*
maize 7Y	82	6	7	5	**Organic substance metabolism**, ***cellular process***, **metabolism**
maize 29Y	91	23	14	7	*Organonitrogen compound metabolism,* **organic substance metabolism**, **metabolism**, ***nitrogen compound metabolism***
rapa 5Y	1277	859	405	142	**Protein phosphorylation**, cellular metabolism, response to chemical, carbohydrate derivative biosynthesis, organic substance transport, **cellular process**, organic substance catabolism, **metabolism**, regulation of biological quality, lipid metabolism
rapa 16Y	1420	1097	343	122	**Protein phosphorylation**, cellular metabolism, response to chemical, carbohydrate derivative biosynthesis, **cellular process**, organic substance catabolism, **metabolism**, organic substance transport, regulation of biological quality, lipid metabolism
sunflower 6Y	608	313	71	25	***Protein phosphorylation***, **organic substance metabolism**, **cellular process**, **metabolism**, **nitrogen compound metabolism**, localization, transport, response to stimulus, biosynthesis, *cellular component organization*
sunflower 27Y	625	318	88	37	**Organic substance metabolism**, *macromolecule metabolism*, **metabolism**, **cellular process**, **nitrogen compound metabolism**, transport, biosynthesis, localization, response to stimulus, *oxidation-reduction process*

Table S8b. Summary of gene ontological (GO) analysis of all fixed dSNPs for each storage year (SY) group of the seven collections. Biological processes in bold represent the most common ones across seven SY group pairs, and biological processes in italics represent the ones unique within each SY group pair. Several major biological processes such as cellular process, macromolecule metabolism, nitrogen compound metabolism, and metabolism were identified across seven SY group pairs and some distinct biological processes for each SY group pair.

Group	No. of fixed dSNPs	No. of uniq genes	No. of GO terms	No. of Bio_Pro	Major Bio_Processes
barley 10Y	31	27	31	7	***Cellular process**, **macromolecule metabolism**, **nitrogen compound metabolism**, **organic substance metabolism***
barley 27Y	42	37	2	1	**Metabolism**
wheat 20Y	89	114	43	21	Biological regulation, cellular component organization or biogenesis, **cellular metabolism**, **cellular process**, **macromolecule metabolism**, **metabolism**, **nitrogen compound metabolism**
wheat 25Y	68	77	41	13	Biological regulation, cellular component organization or biogenesis, **cellular metabolism**, **cellular process**, **macromolecule metabolism**, **metabolism**, **nitrogen compound metabolism**
oat 16Y	320	334	184	109	**Cellular metabolism**, **cellular process**, **macromolecule metabolism**, **metabolism**, **nitrogen compound metabolism**, **organic substance metabolism**, primary metabolism
oat 21Y	287	266	183	106	**Cellular metabolism**, **cellular process**, **macromolecule metabolism**, **metabolism**, **nitrogen compound metabolism**, **organic substance metabolism**, *organonitrogen compound*
soybean 10Y	19	20	0	0	No significant GO terms
soybean 20Y	18	20	0	0	No significant GO terms
maize 7Y	5	5	3	1	***Cellular process***
maize 29Y	2	1	0	0	No significant GO terms
rapa 5Y	45	88	29	9	***Cellular metabolism***, **cellular process**, **metabolism**, **nitrogen compound metabolism**, *organonitrogen compound metabolism*
rapa 16Y	35	75	31	11	**Cellular process**, ***macromolecule metabolism***, **metabolism**, **nitrogen compound metabolism**, ***organic substance metabolism***
sunflower 6Y	41	33	0	0	No significant GO terms
sunflower 27Y	42	43	13	7	***Cellular process**, **macromolecule metabolism**, **metabolism**, **nitrogen compound metabolism**, **organic substance metabolism***

Table S9. List of the Kruskal-Wallis tests for the six estimates inferred from each SY (or storage year) group pair of the seven collections. The estimates and their standard deviations are 1) dSNP proportion, 2) fixed dSNP proportion, 3) individual total mutation burden, 4) individual homozygous mutation burden, 5) individual heterozygous mutation burden, and 6) alpha-dfe for adaptive mutation. The standard deviations of dSNP and fixed dSNP proportions were obtained from 10 jackknife sub-samples of the SY group in each collection. The standard deviations of alpha-dfe were obtained from 30 bootstrapped samples of each SY group. The statistical significance is shown with *,**, or *** for *P* <0.05, 0.01, or 0.001, respectively.

Variants and	Barley		Wheat		Oat		Soybean		Maize		Rapa		Sunflower	
other estimates	10Y	27Y	20Y	25Y	16Y	21Y	10Y	20Y	7Y	29Y	5Y	16Y	6Y	27Y
Sample size	16	16	12	12	10	10	15	15	18	18	20	20	15	15
dSNP proportion														
Estimate	0.001125	0.001169	0.003318	0.003587	0.020834	0.015865	0.001386	0.001340	0.000065	0.000071	0.001157	0.001368	0.000753	0.000788
Standard deviation	0.000013	0.000017	0.000031	0.000052	0.000364	0.002648	0.000021	0.000020	0.000001	0.000001	0.000015	0.000014	0.000006	0.000008
Difference	0.000044		0.000269		-0.004969		-0.000047		0.000006		0.000212		0.000035	
P value	***		***		**		***		***		***		***	
Fixed dSNP proportion														
Estimate	0.000085	0.000119	0.000230	0.000233	0.000499	0.000346	0.000080	0.000078	0.000004	0.000002	0.000042	0.000035	0.000039	0.000052
Standard deviation	0.000005	0.000006	0.000012	0.000008	0.000024	0.000042	0.000005	0.000003	0.000000	0.000001	0.000001	0.000001	0.000002	0.000003
Difference	0.000034		0.000003		-0.000153		-0.000002		-0.000002		-0.000007		0.000013	
P value	***				***				***		***		***	
Total Burden														
Estimate	0.3001	0.3002	0.3309	0.3205	0.2266	0.2047	0.3778	0.4100	0.4253	0.4435	0.2637	0.2516	0.3262	0.3626
Standard deviation	0.0210	0.0245	0.0286	0.0165	0.0157	0.0194	0.0325	0.0223	0.0327	0.0259	0.0241	0.0184	0.0168	0.0153
Difference	0.0001		-0.0104		-0.0219		0.0321		0.0181		-0.0121		0.0363	
P value					*		**		*		*		***	
Homozygous Burden														
Estimate	0.2213	0.2117	0.2444	0.2355	0.0858	0.0692	0.2108	0.2219	0.2340	0.2516	0.1600	0.1491	0.1967	0.2247
Standard deviation	0.0240	0.0254	0.0265	0.0209	0.01	0.0152	0.0316	0.0234	0.0332	0.0295	0.0398	0.0311	0.0169	0.0186
Difference	-0.0096		-0.0089		-0.0166		0.0112		0.0176		-0.0109		0.0279	
P value					*								***	
Heterzygous Burden														
Estimate	0.0788	0.0885	0.0866	0.0849	0.1407	0.1355	0.1671	0.1880	0.1913	0.1919	0.1037	0.1025	0.1295	0.1379
Standard deviation	0.0088	0.0066	0.0053	0.0060	0.0088	0.0075	0.0071	0.0166	0.0245	0.0163	0.0255	0.0185	0.0186	0.0203
Difference	0.0097		-0.0017		-0.0052		0.0210		0.0006		-0.0011		0.0084	
P value	**						**							
Adaptive mutation														
Estimate of alpha-df	0.8275	0.7863	0.9452	0.9444	0.9013	0.3577	0.9356	0.9309	0.8293	0.8080	0.8204	0.7891	0.8989	0.8886
Standard deviation	0.0236	0.0563	0.0021	0.0092	0.0052	0.0785	0.0058	0.0054	0.0171	0.0247	0.0068	0.0184	0.0026	0.0056
Difference	-0.0412		-0.0008		-0.5436		-0.0047		-0.0212		-0.0313		-0.0103	
P value	***				***		**		***		***		***	

Table S10. List of the Kruskal-Wallis tests for the six estimates inferred from each RF (or regeneration frequency) group pair of the seven collections. The estimates and their standard deviations are 1) dSNP proportion, 2) fixed dSNP proportion, 3) individual total mutation burden, 4) individual homozygous mutation burden, 5) individual heterozygous mutation burden, and 6) alpha-dfe for adaptive mutation. The standard deviations of dSNP and fixed dSNP proportions were obtained from 10 jackknife sub-samples of the RF group in each collection. The standard deviations of alpha-dfe were obtained from 30 bootstrapped samples of each RF group. The statistical significance is shown with *,**, or *** for *P* <0.05, 0.01, or 0.001, respectively.

Variants and	Barley		Wheat		Oat		Soybean		Maize		Rapa		Sunflower	
other estimates	RF1	RF2	RF1	RF2	RF1	RF2	RF1	RF2	RF1	RF2	RF1	RF2	RF1	RF2
Sample size	24	24	18	18	9	9	14	14	18	18	15	15	27	27
dSNP proportion														
Estimate	0.001155	0.001178	0.003439	0.003249	0.022757	0.020438	0.001367	0.001505	0.000070	0.000069	0.001229	0.001214	0.000709	0.000761
Standard deviation	0.000009	0.000010	0.000026	0.000035	0.000560	0.000819	0.000014	0.000031	0.000001	0.000001	0.000010	0.000007	0.000005	0.000005
Difference	0.000022		-0.000190		-0.002319		0.000138		-0.000002		-0.000015		0.000052	
P value	***		***		***		***		*		**		***	
Fixed dSNP proportion														
Estimate	0.000070	0.000061	0.000181	0.000172	0.000507	0.000535	0.000073	0.000096	0.000002	0.000007	0.000048	0.000055	0.000032	0.000027
Standard deviation	0.000003	0.000002	0.000007	0.000010	0.000034	0.000035	0.000005	0.000005	0.000000	0.000001	0.000002	0.000002	0.000001	0.000001
Difference	-0.000008		-0.000008		0.000028		0.000023		0.000004		0.000006		-0.000005	
P value	***		*				***		***		***		***	
Total Burden														
Estimate	0.2553	0.2351	0.3168	0.2945	0.2380	0.2373	0.3752	0.3720	0.4570	0.4632	0.3114	0.3026	0.3132	0.2654
Standard deviation	0.0185	0.0139	0.0417	0.0202	0.0221	0.0408	0.0274	0.0275	0.0290	0.0301	0.0329	0.0201	0.0133	0.0155
Difference	-0.0203		-0.0223		-0.0007		-0.0031		0.0061		-0.0087		-0.0478	
P value	***												***	
Homozygous Burden														
Estimate	0.1787	0.1604	0.2269	0.2107	0.0805	0.0884	0.2145	0.2139	0.2661	0.2758	0.1889	0.2022	0.1877	0.1663
Standard deviation	0.0185	0.0152	0.0465	0.0224	0.0161	0.0299	0.0241	0.0260	0.0389	0.0314	0.0500	0.0462	0.0170	0.0177
Difference	-0.0183		-0.0162		0.0079		-0.0006		0.0097		0.0133		-0.0214	
P value	***												***	
Heterzygous Burden														
Estimate	0.0767	0.0747	0.0899	0.0838	0.1575	0.1489	0.1607	0.1581	0.1909	0.1873	0.1225	0.1005	0.1255	0.0990
Standard deviation	0.0064	0.0058	0.0105	0.0048	0.0123	0.0119	0.0127	0.0107	0.0207	0.0246	0.0280	0.0305	0.0169	0.0117
Difference	-0.0020		-0.0061		-0.0086		-0.0025		-0.0036		-0.0220		-0.0265	
P value			*								*		***	
Adaptive mutation														
Estimate of alpha-df	0.8687	0.8775	0.9296	0.9327	0.8885	0.8832	0.9328	0.9332	0.8533	0.8502	0.7922	0.8292	0.8973	0.8781
Standard deviation	0.0335	0.0082	0.0046	0.0013	0.0042	0.0102	0.0049	0.0051	0.0125	0.0054	0.0048	0.0052	0.0050	0.0062
Difference	0.0088		0.0032		-0.0053		0.0004		-0.0030		0.0370		-0.0192	
P value			**						*		***		***	

Table S11. List of the Kruskal-Wallis tests for the six estimates inferred from each GD (or germination difference) group pair of the seven collections. The estimates and their standard deviations are 1) dSNP proportion, 2) fixed dSNP proportion, 3) individual total mutation burden, 4) individual homozygous mutation burden, 5) individual heterozygous mutation burden, and 6) alpha-dfe for adaptive mutation. The standard deviations of dSNP and fixed dSNP proportions were obtained from 10 jackknife sub-samples of the GD group in each collection. The standard deviations of alpha-dfe were obtained from 30 bootstrapped samples of each GD group. The statistical significance is shown with *,**, or *** for P <0.05, 0.01, or 0.001, respectively.

Variants and	Barley		Wheat		Oat		Soybean		Maize		Rapa		Sunflower	
other estimates	68G	91G	26G	72G	84G	96G	49G	86G	70G	94G	69G	96G	50G	86G
Sample size	17	17	17	17	16	16	15	15	15	15	12	12	14	14
dSNP proportion														
Estimate	0.001150	0.001160	0.003479	0.003471	0.022225	0.023473	0.001335	0.001390	0.000076	0.000067	0.001241	0.001220	0.000793	0.000786
Standard deviation	0.000012	0.000010	0.000035	0.000022	0.000309	0.000367	0.000014	0.000021	0.000001	0.000001	0.000011	0.000008	0.000006	0.000010
Difference	0.000010		-0.000008		0.0012477		0.000055		-0.000009		-0.000021		-0.000006	
P value					***		***		***		***			
Fixed dSNP proportion														
Estimate	0.000094	0.000092	0.000235	0.000141	0.000329	0.000300	0.000087	0.000081	0.000005	0.000005	0.000047	0.000055	0.000050	0.000042
Standard deviation	0.000004	0.000004	0.000013	0.000003	0.000008	0.000007	0.000003	0.000004	0.000001	0.000000	0.000002	0.000002	0.000002	0.000002
Difference	-0.000002		-0.000094		-0.000030		-0.000006		0.000000		0.000008		-0.000007	
P value			***		***		**				***		***	
Total Burden														
Estimate	0.2806	0.3003	0.3552	0.2616	0.1723	0.1691	0.4032	0.3758	0.4923	0.4461	0.2863	0.3184	0.3425	0.3284
Standard deviation	0.0229	0.0154	0.0524	0.0211	0.0209	0.0154	0.0316	0.0269	0.0314	0.0196	0.0189	0.0312	0.0168	0.0214
Difference	0.0198		-0.0935		-0.0032		-0.0273		-0.0462		0.0321		-0.0142	
P value	*		***				*		***		**			
Homozygous Burden														
Estimate	0.2073	0.2169	0.2659	0.1831	0.0522	0.0473	0.2321	0.2194	0.2878	0.2486	0.1802	0.1917	0.2202	0.2086
Standard deviation	0.0226	0.0167	0.0577	0.0218	0.0135	0.0108	0.0287	0.0281	0.0341	0.0342	0.0342	0.0308	0.0211	0.0242
Difference	0.0096		-0.0828		-0.0049		-0.0127		-0.0392		0.0115		-0.0116	
P value			***						**					
Heterzygous Burden														
Estimate	0.0733	0.0834	0.0892	0.0785	0.1202	0.1217	0.1711	0.1565	0.2045	0.1975	0.1061	0.1267	0.1224	0.1198
Standard deviation	0.0057	0.0052	0.0101	0.0027	0.0091	0.0068	0.0132	0.0110	0.0181	0.0223	0.0254	0.0152	0.0120	0.0119
Difference	0.0101		-0.0107		0.0015		-0.0146		-0.0069		0.0206		-0.0026	
P value	***		***				**				*			
Adaptive mutation														
Estimate of alpha-dfe	0.8671	0.7942	0.9085	0.9170	0.9268	0.9143	0.9466	0.9416	0.8138	0.7645	0.7724	0.8057	0.8841	0.8729
Standard deviation	0.0091	0.0790	0.0039	0.0045	0.0031	0.0043	0.0036	0.0049	0.0279	0.0351	0.0197	0.0243	0.0045	0.0057
Difference	-0.0729		0.0085		-0.0125		-0.0050		-0.0494		0.0333		-0.0112	
P value	***		***		***		***		***		***		***	

E: Figure S1 to S6

Fig. S1. The flowchart showing the major steps used to identify putative deleterious SNPs in this study. The whole process required a SIFT database and GERP++ RS score data for one species. Our research effort was largely spent on the development of SIFT databases for five plant species (oat, soybean, maize, rapa, and sunflower) and of GERP++ RS scores for seven species by performing multiple sequence alignments of each species genome against 12 other species genomes (Table S5a).

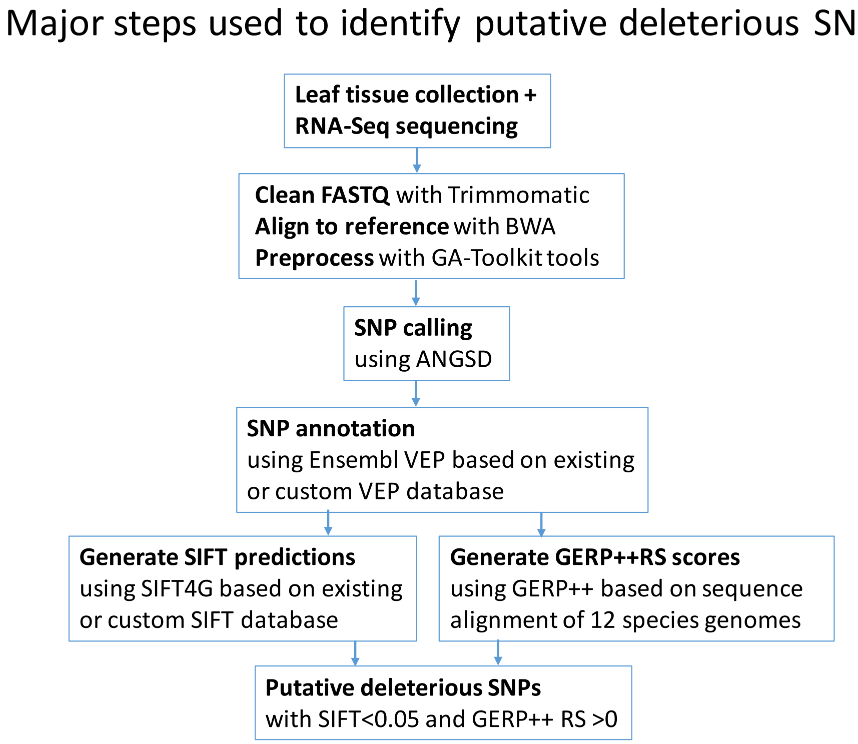

Fig. S2. The frequency distributions of deleterious SNPs (dSNPs) identified in seven collections each with 70 individual samples. The four left panels are the selfing plants and the three right panels are the outcrossing plants. In each panel, the total number of dSNPs and their mean and median allelic frequencies are also presented. Clearly, a majority of the identified dSNPs had a low allelic frequency, but fixed dSNPs were also observed in each collection. These patterns are more obvious in the barley, rapa, and wheat collections.

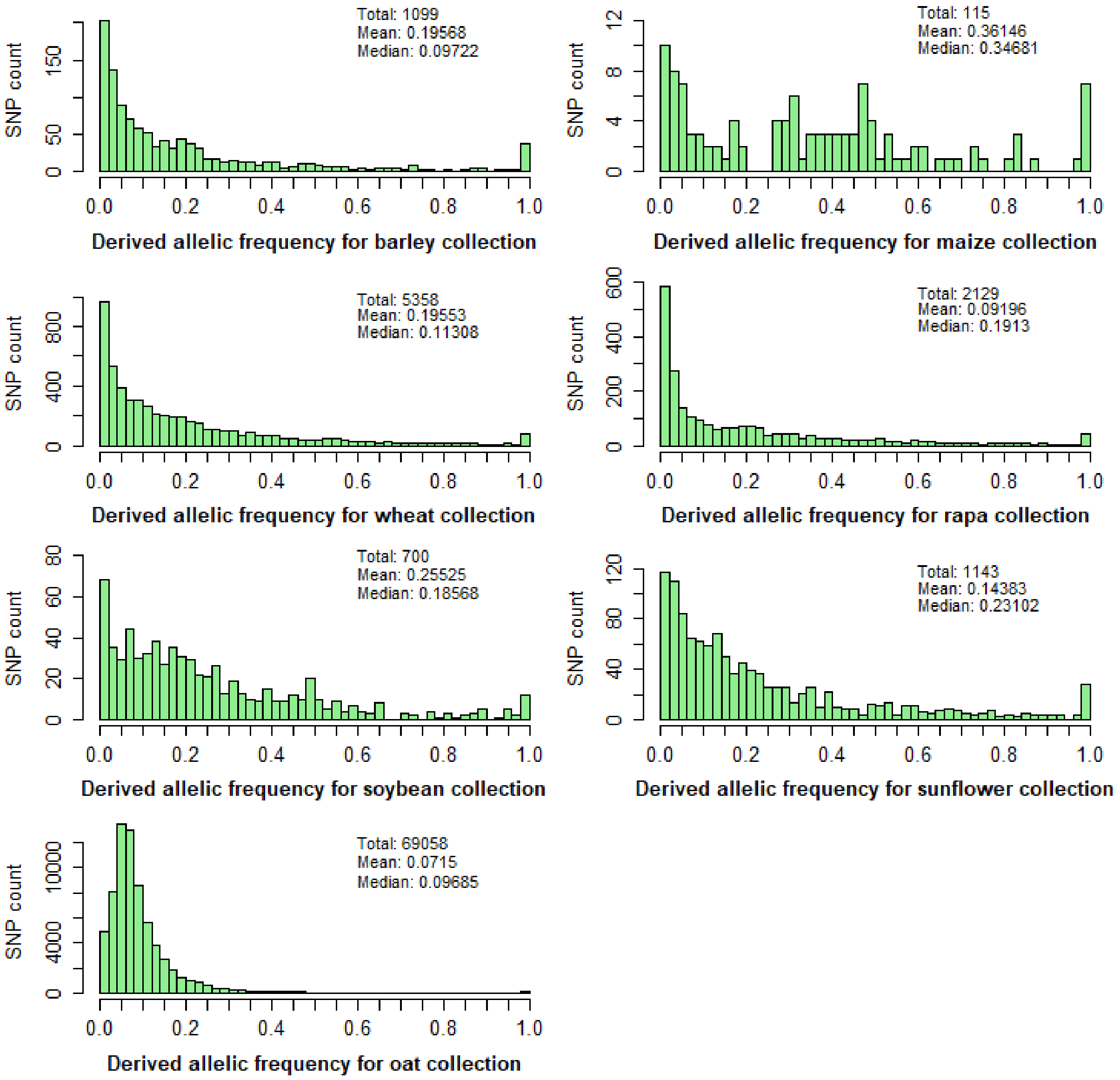

Fig. S3a. No marked associations between the mutation burdens per deleterious locus and the storage years since the last regeneration for the assayed barley and wheat samples. Three mutation burdens per deleterious locus (total, heterozygous, homozygous) were presented for each sample in each collection. For each panel, the samples were ordered for the burden extent and shown in their storage years since the last regeneration on the X-axis.

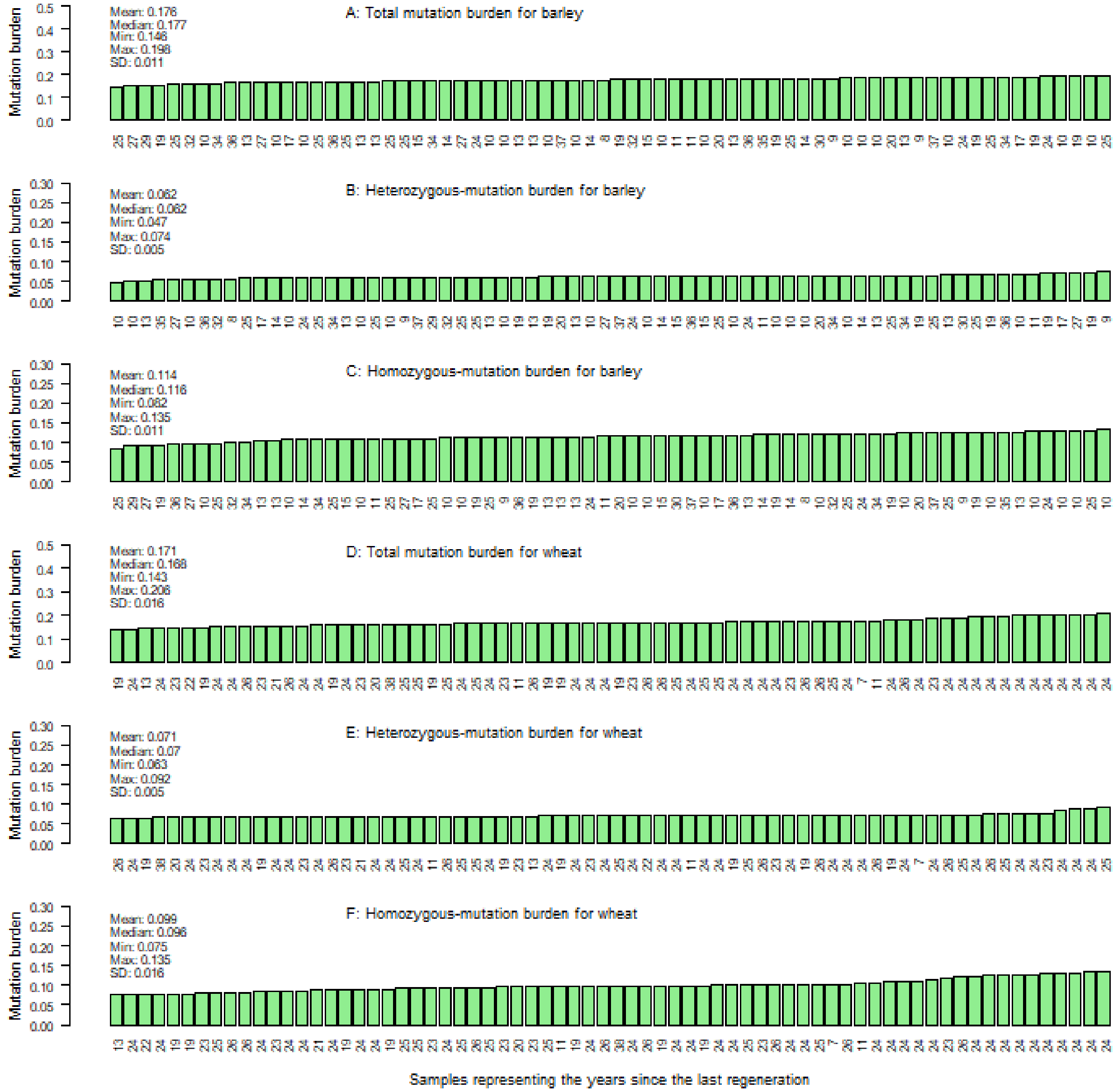

Fig. S3b. No marked associations between the mutation burdens per deleterious locus and the storage years since the last regeneration for the assayed soybean and maize samples. Three mutation burdens per deleterious locus (total, heterozygous, homozygous) were presented for each sample in each collection. For each panel, the samples were ordered for the burden extent and shown in their storage years since the last regeneration on the X-axis.

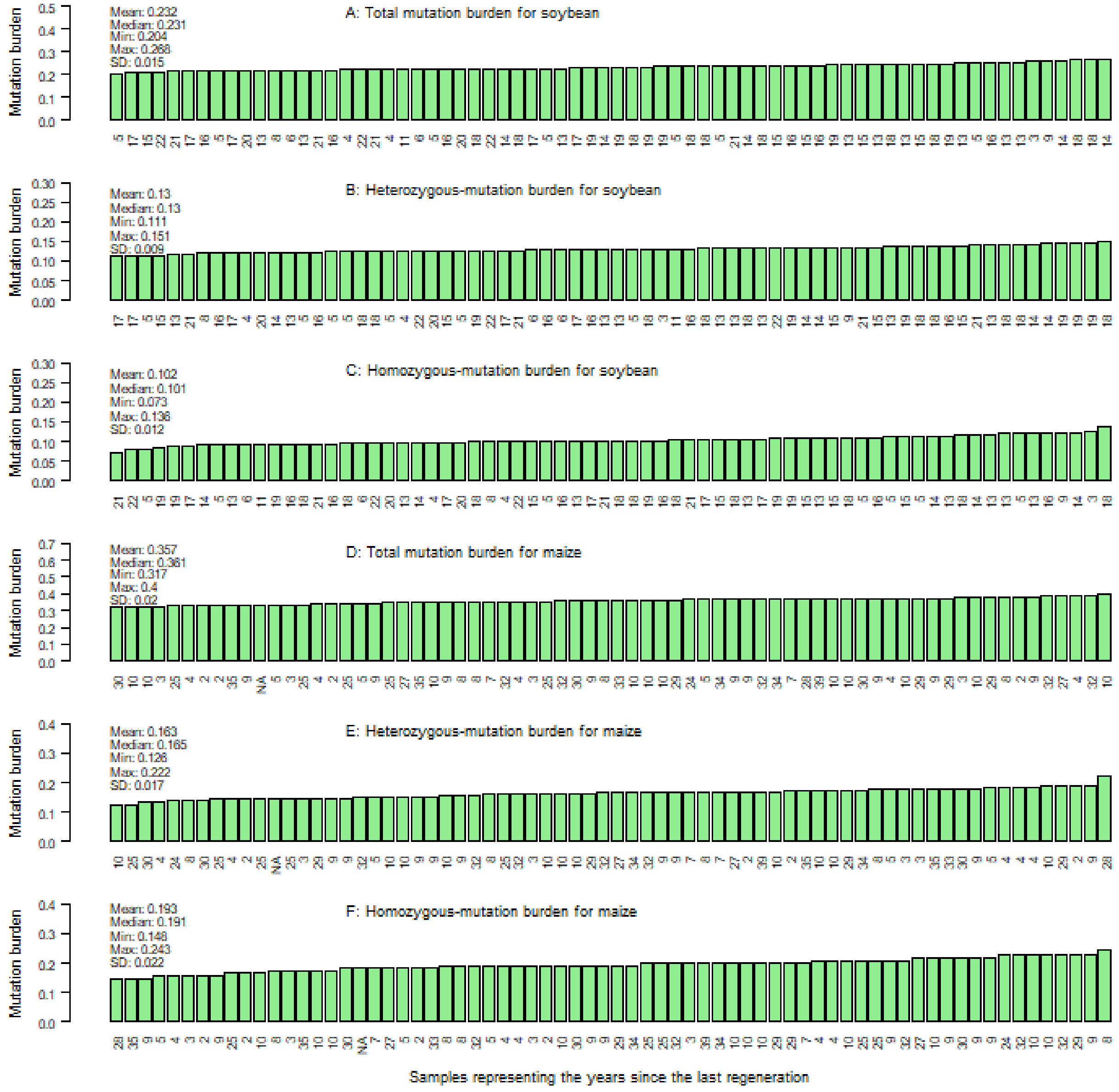

Fig. S3c. No marked associations between the mutation burdens per deleterious locus and the storage years since the last regeneration for the assayed rapa and sunflower samples. Three mutation burdens per deleterious locus (total, heterozygous, homozygous) were presented for each sample in each collection. For each panel, the samples were ordered for the burden extent and shown in their storage years since the last regeneration on the X-axis..

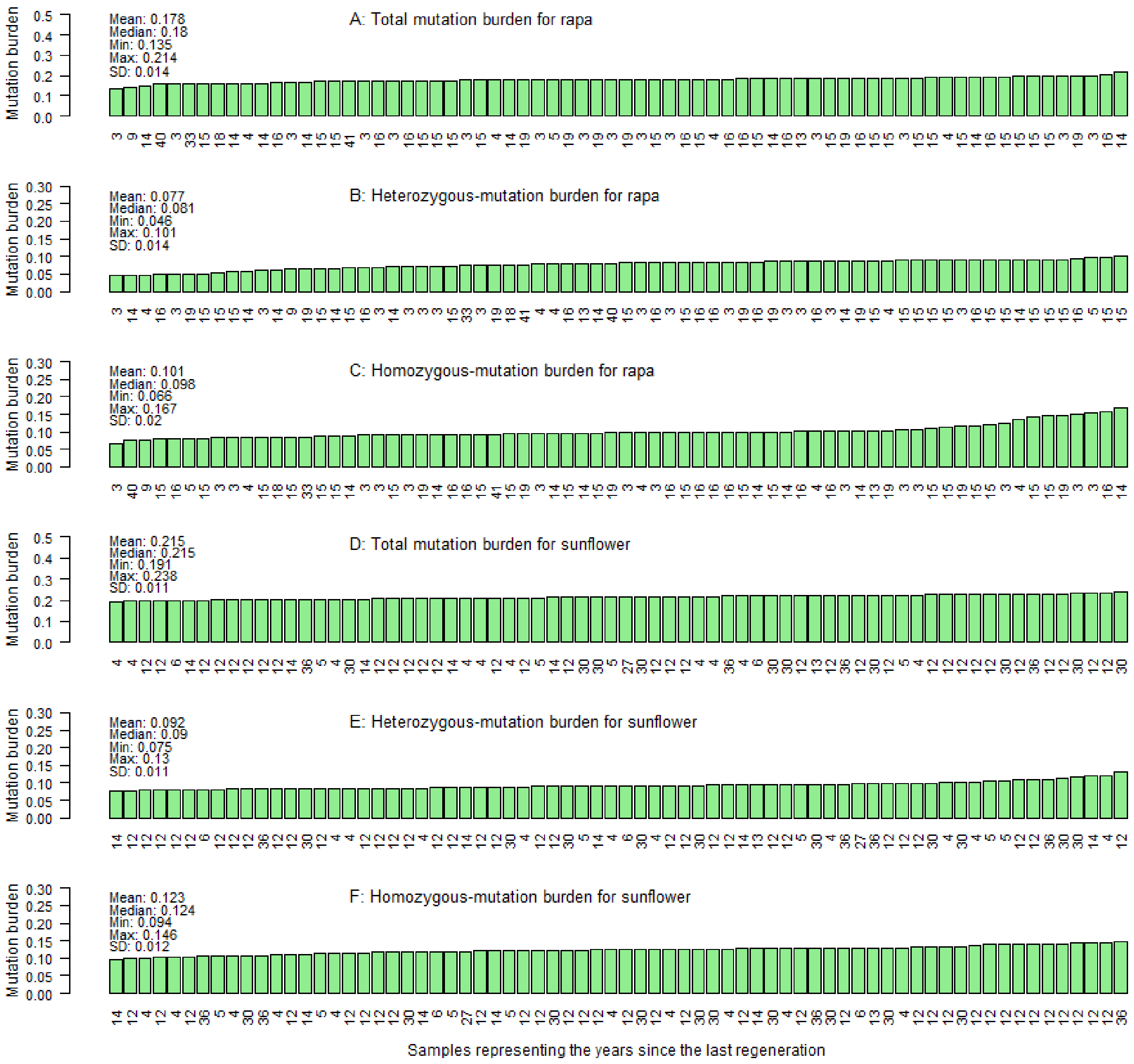

Fig. S3d. No marked associations between the mutation burdens per deleterious locus and the storage years since the last regeneration for the assayed oat samples. Three mutation burdens per deleterious locus (total, heterozygous, homozygous) were presented for each sample in each collection. For each panel, the samples were ordered for the burden extent and shown in their storage years since the last regeneration on the X-axis.

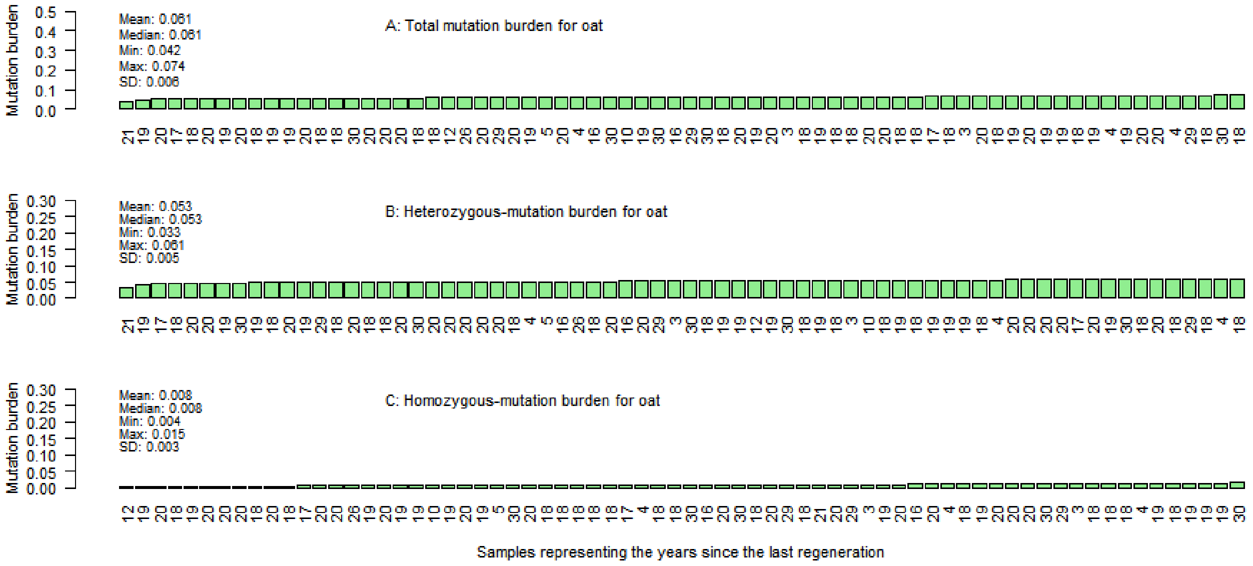

Fig. S4a. Regressions of individual mutation burdens per deleterious locus (total, heterozygous, homozygous) over the years of the accession acquisition in the seven collections. Four collections (barley, soybean, rapa, and sunflower) displayed trends of larger total mutation burdens for the samples acquired earlier, while three collections (wheat, maize, and oat) showed trends of lower total mutation burden in the samples acquired earlier. The regression panels highlighted in pink were statistically significant at $p<0.05$.

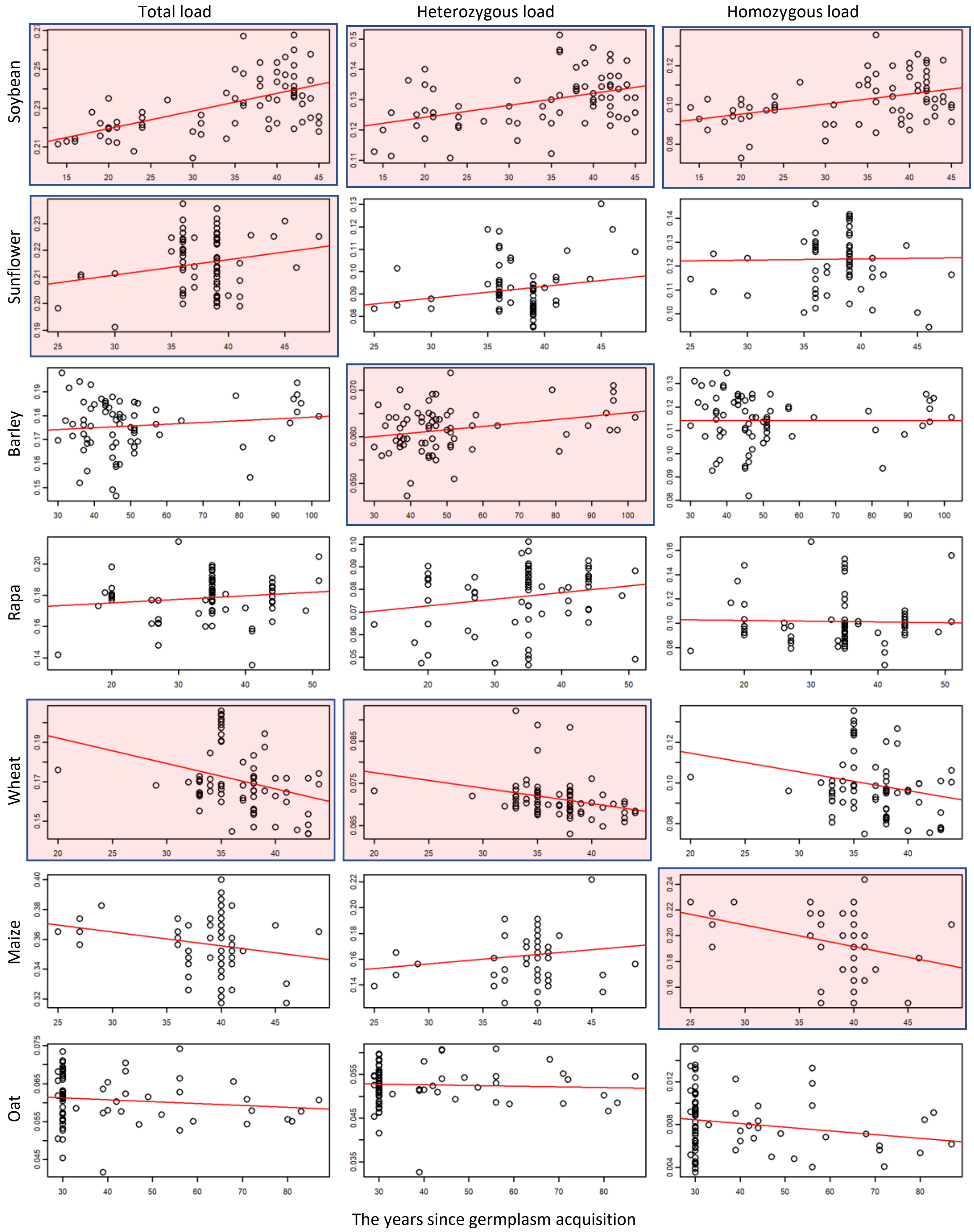

Fig. S4b. Regressions of individual mutation burdens per deleterious locus (total, heterozygous, homozygous) over the years of storage since the last field regeneration in the seven collections. Three collections (wheat, maize, and sunflower) showed trends of larger total mutation burden in the samples stored longer, while four collections (barley, oat soybean, and rapa) displayed trends of lower total mutation burdens for the samples stored longer after the last regeneration. The regression panels highlighted in pink were statistically significant at $p<0.05$.

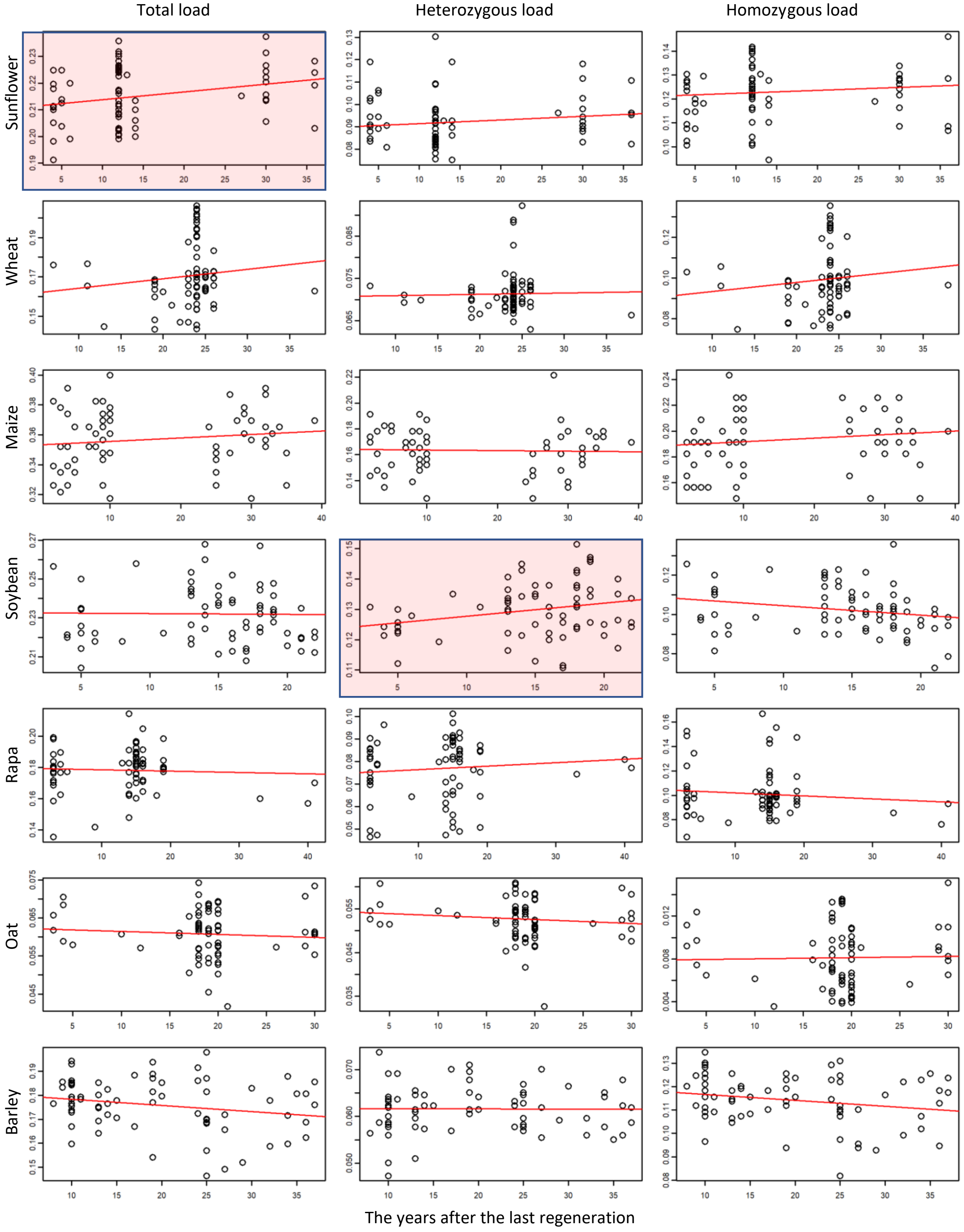

Fig. S4c. Regressions of individual mutation burdens per deleterious locus (total, heterozygous, homozygous) over the accession germination rates in the seven collections. Four collections (wheat, soybean, maize, and sunflower) displayed trends of larger total mutation burden for the samples with lower germinations, while three collections (barley, oat, and rapa) showed trends of larger total mutation burden in the samples with higher germinations. The regression panels highlighted in pink were statistically significant at $p<0.05$.

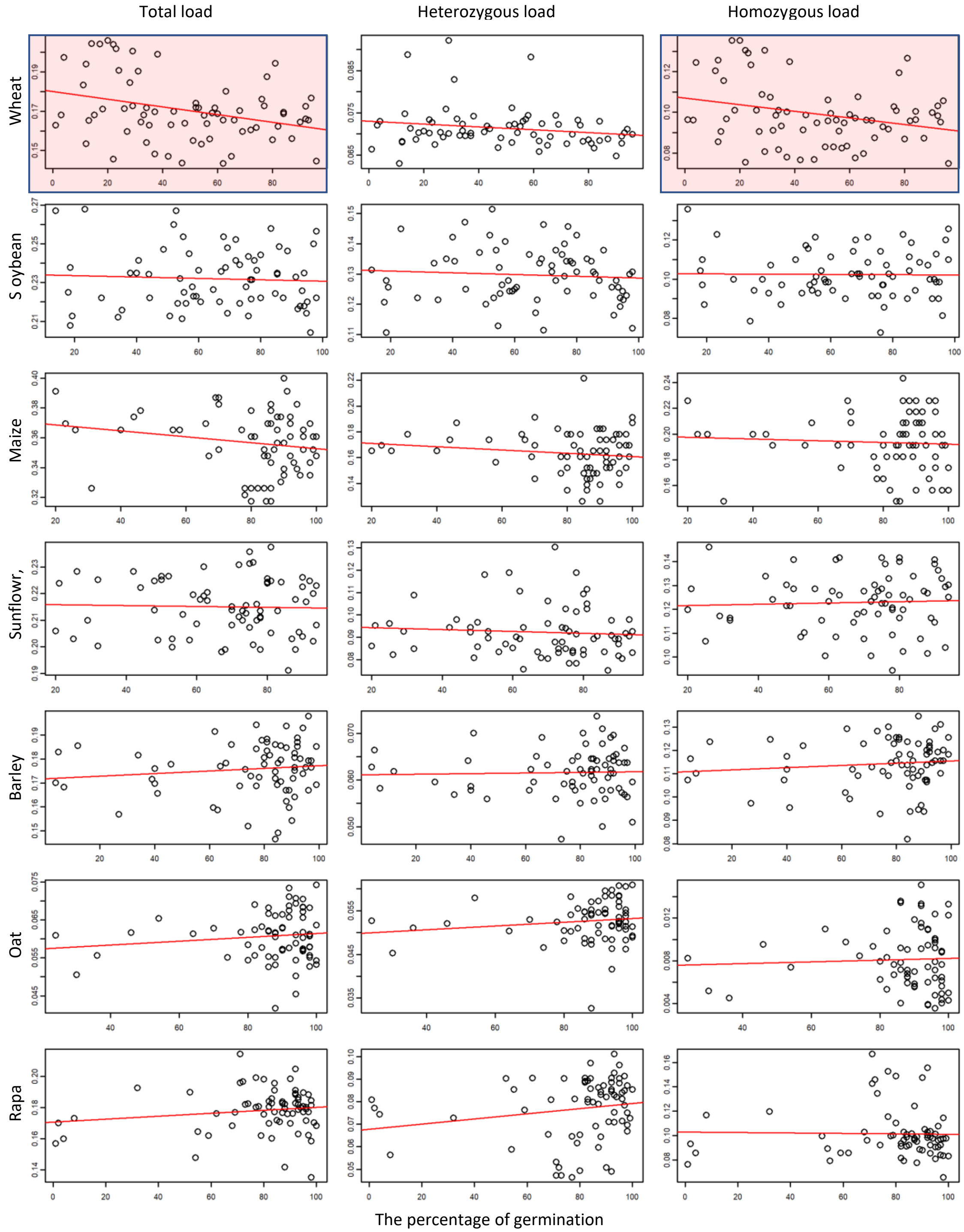

Fig. S5. Venn diagrams showing the comparative counts of deleterious SNPs (dSNPs) that are shared and unique among three groups of samples (two SY group pairs and all of the assayed samples) in each collection. The four left panels are the selfing plants, while the three right panels are the outcrossing plants. Four collections (wheat, maize, rapa, and sunflower) displayed more dSNPs (and unique dSNPs) in the SY2 group of longer storage than the SY1 group. These results indicate that deleterious mutations generally increased with more years of storage in the genebank since the last field regeneration in the seven collections.

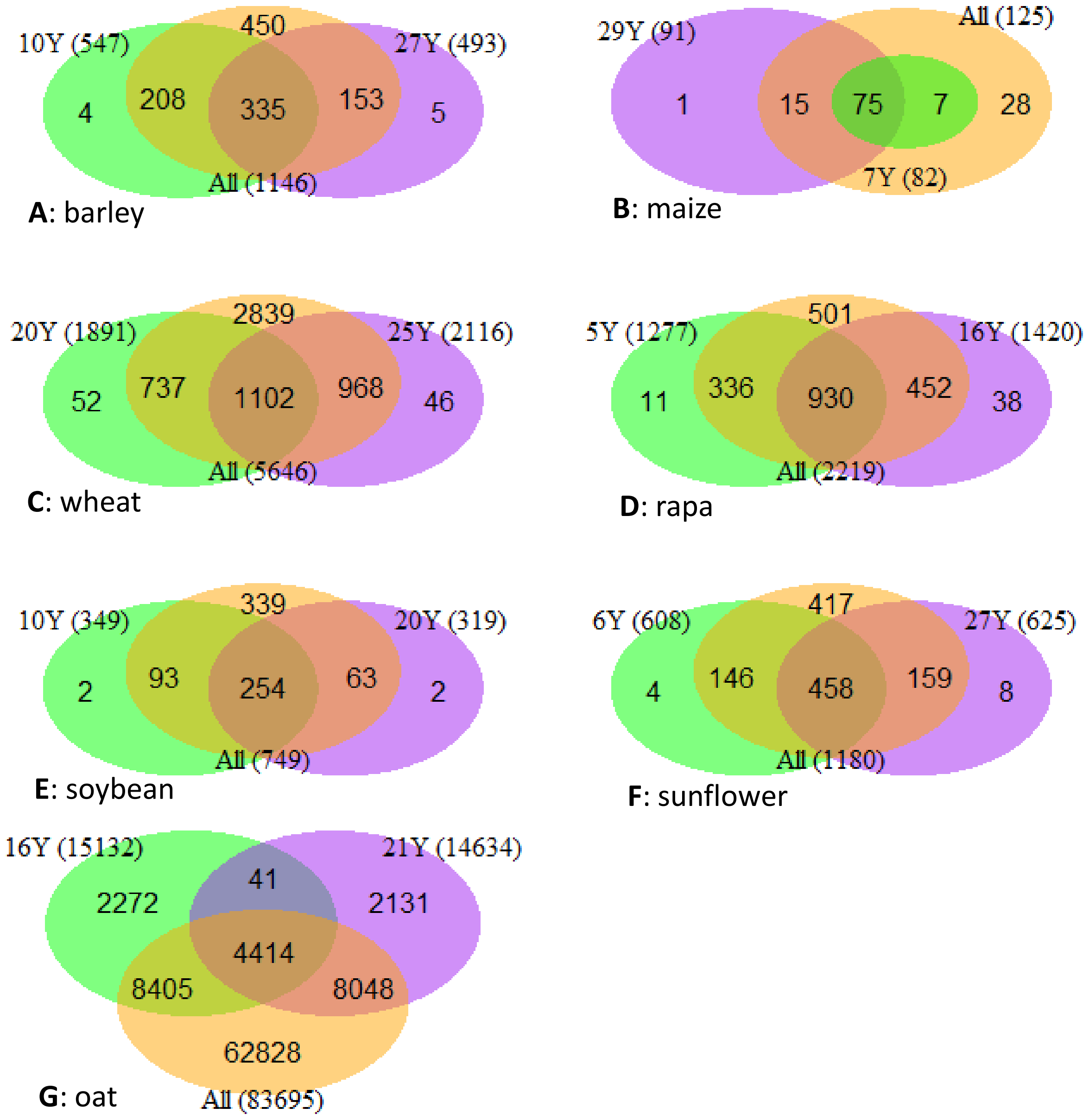

Fig. S6. Comparative allele frequency distributions for deleterious SNPs (dSNPs) identified in the samples of the SY1 (in blue) and SY2 (in red) groups in the seven collections. For example, the blue line and dots in the barley collection represent the allelic frequency distribution for the barley 10Y samples, while the red line and dots are those for the barley 27Y samples. The four left panels are the selfing plants and the three right panels are the outcrossing plants. There were no marked differences in deleterious allelic frequency distribution with respect to the years of storage between SY1 and SY2 groups in each collection. The rapa 16Y (or SY2) samples seemed to have more low allelic frequencies and fixation of dSNPs than the rapa 5Y (or SY1) samples.

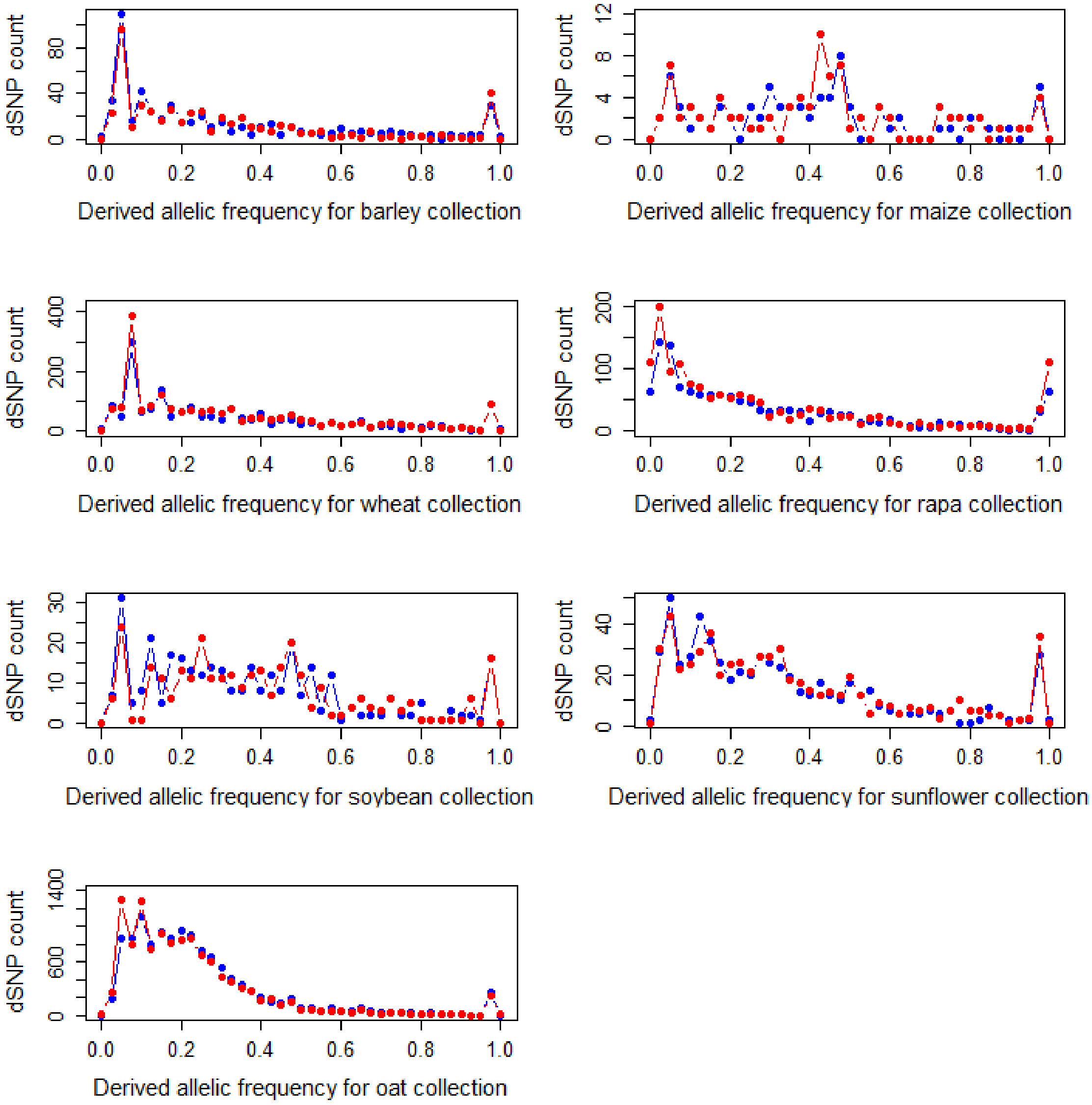

Supplement: msad238_Supplementary_Data [file msad238_supplementary_data.pdf]
